# Targeting m^6^A Modifications Regulating Ferroptosis Offers Novel Therapy in Diseases

**DOI:** 10.1111/cpr.70258

**Published:** 2026-07-17

**Authors:** Lida Du, Joshua S. Fleishman, Shuang Wu, Yuan Zhou, Clara X. Wang, Haitong Wang, Yumin Wang, Hongquan Wang

**Affiliations:** ^1^ Division of Neurobiology Johns Hopkins University Baltimore Maryland USA; ^2^ Department of Pharmaceutical Sciences College of Pharmacy and Health Sciences, St. John's University Queens New York USA; ^3^ Department of Neurology Zhongnan Hospital of Wuhan University Wuhan China; ^4^ School of Arts and Sciences, University of Pennsylvania Philadelphia Pennsylvania USA; ^5^ Department of Respiratory and Critical Care Medicine Aerospace Center Hospital, Peking University Aerospace School of Clinical Medicine Beijing China; ^6^ Department of Geriatrics Aerospace Center Hospital, Peking University Aerospace School of Clinical Medicine Beijing China; ^7^ Aerospace Medical Center, Aerospace Center Hospital Beijing China

**Keywords:** diseases, epigenetic modifications, ferroptosis, N_6_‐methyladenosine

## Abstract

Ferroptosis, a regulated form of cell death driven by iron‐dependent lipid peroxidation, emerged as a critical process in the pathogenesis of various diseases, including neurological, cardiovascular, hepatic, pulmonary, and renal disorders. Simultaneously, N6‐methyladenosine (m^6^A), the most abundant reversible RNA modification in eukaryotes, plays a pivotal role in gene regulation by modulating RNA stability, translation, and degradation. Evidence increasingly links m^6^A modifications to the transcriptional and post‐transcriptional regulation of ferroptosis, highlighting their importance in disease mechanisms. This review explores the molecular mechanisms underpinning m^6^A modifications and ferroptosis, detailing their interactions in disease progression. Additionally, it evaluates the therapeutic promise of targeting m^6^A regulators to modulate ferroptosis, offering novel approaches for managing diseases with diverse etiologies. By clarifying the role of m^6^A in ferroptosis, this review underscores the promise of m^6^A‐focused therapeutic strategies in advancing treatment for diseases where ferroptosis plays a key pathological role.

## Graphical abstract

m^6^A RNA modification regulates ferroptosis by balancing iron metabolism, lipid peroxidation, and antioxidant defenses. Dysregulated m^6^A signaling disrupts pro‐ and anti‐ferroptotic factors, leading to excess ROS, Fe^3+^ accumulation, and lipid peroxidation–driven cell death. Targeting m^6^A‐mediated ferroptotic regulation represents a promising therapeutic approach for diverse diseases.

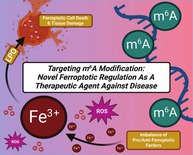

## 1 Introduction

Ferroptosis is a regulated form of cell death characterised by iron‐dependent lipid peroxidation and the accumulation of reactive oxygen species, leading to oxidative damage of cellular membranes. Unlike apoptosis, necrosis, or autophagy, ferroptosis is primarily driven by metabolic disturbances in iron homeostasis, lipid metabolism, and antioxidant defence systems [1, 2, 3]. Since its formal definition, ferroptosis has been increasingly recognised as a key mechanism contributing to the pathogenesis of multiple diseases.

Recent evidence indicates that dysregulated ferroptosis contributes to tissue injury and disease progression across different biological systems. In neurodegenerative disorders, excessive ferroptotic signalling promotes neuronal loss and oxidative damage, whereas in cancer, ferroptosis represents a therapeutic vulnerability that can be exploited to eliminate tumour cells [4, 5, 6].

At the molecular level, ferroptosis is governed by several interconnected metabolic pathways. Iron metabolism determines the availability of redox‐active iron that drives lipid peroxidation, while enzymes such as ACSL4 and LPCAT3 facilitate the synthesis of polyunsaturated phospholipids that serve as substrates for oxidative damage [7, 8, 9]. In parallel, antioxidant systems including the glutathione peroxidase 4 (GPX4)–glutathione axis and the cystine/glutamate antiporter system Xc− act as major protective mechanisms that suppress lipid peroxide accumulation and ferroptotic cell death [10, 11, 12].

In recent years, increasing attention has been directed toward epitranscriptomic regulation of ferroptosis, particularly through N6‐methyladenosine (m^6^A) RNA modification. m^6^A is the most abundant internal RNA modification in eukaryotic mRNA and plays a fundamental role in controlling RNA metabolism, including mRNA stability, splicing, nuclear export, and translation efficiency [13, 14, 15]. The dynamic regulation of m^6^A is mediated by three classes of proteins: methyltransferases (‘writers’), demethylases (‘erasers’), and m^6^A‐binding proteins (‘readers’), which collectively determine the fate of m^6^A‐modified transcripts [16, 17, 18].

Accumulating studies demonstrate that m^6^A modification regulates ferroptosis by modulating the expression of key ferroptotic genes, including SLC7A11, GPX4, ACSL4, and NRF2, as well as genes involved in iron metabolism [19, 20, 21, 22]. For example, m^6^A writers such as METTL3 and METTL14 can either promote or inhibit ferroptosis depending on cellular context, whereas demethylases such as FTO and ALKBH5 regulate the stability and translation of transcripts involved in oxidative stress responses and lipid metabolism [23, 24, 25, 26].

Importantly, the regulatory effects of m^6^A on ferroptosis are highly context dependent. The same m^6^A regulator may exert opposite effects across different tissues or disease states, reflecting differences in metabolic environment, downstream targets, and the expression of specific reader proteins. This context specificity represents a major challenge in interpreting current findings and highlights the need for a unified mechanistic framework [27, 28, 29].

In this review, we synthesise current evidence to define how m^6^A RNA modification regulates ferroptosis through key molecular pathways, including iron metabolism, lipid peroxidation, antioxidant defense, and associated signalling pathways. By integrating findings across diverse biological contexts, we aim to provide a mechanism‐centred perspective that clarifies both shared and distinct regulatory patterns and highlights potential opportunities for therapeutic intervention.

## 2 Molecular Mechanisms of Ferroptosis

**FIGURE 1:**
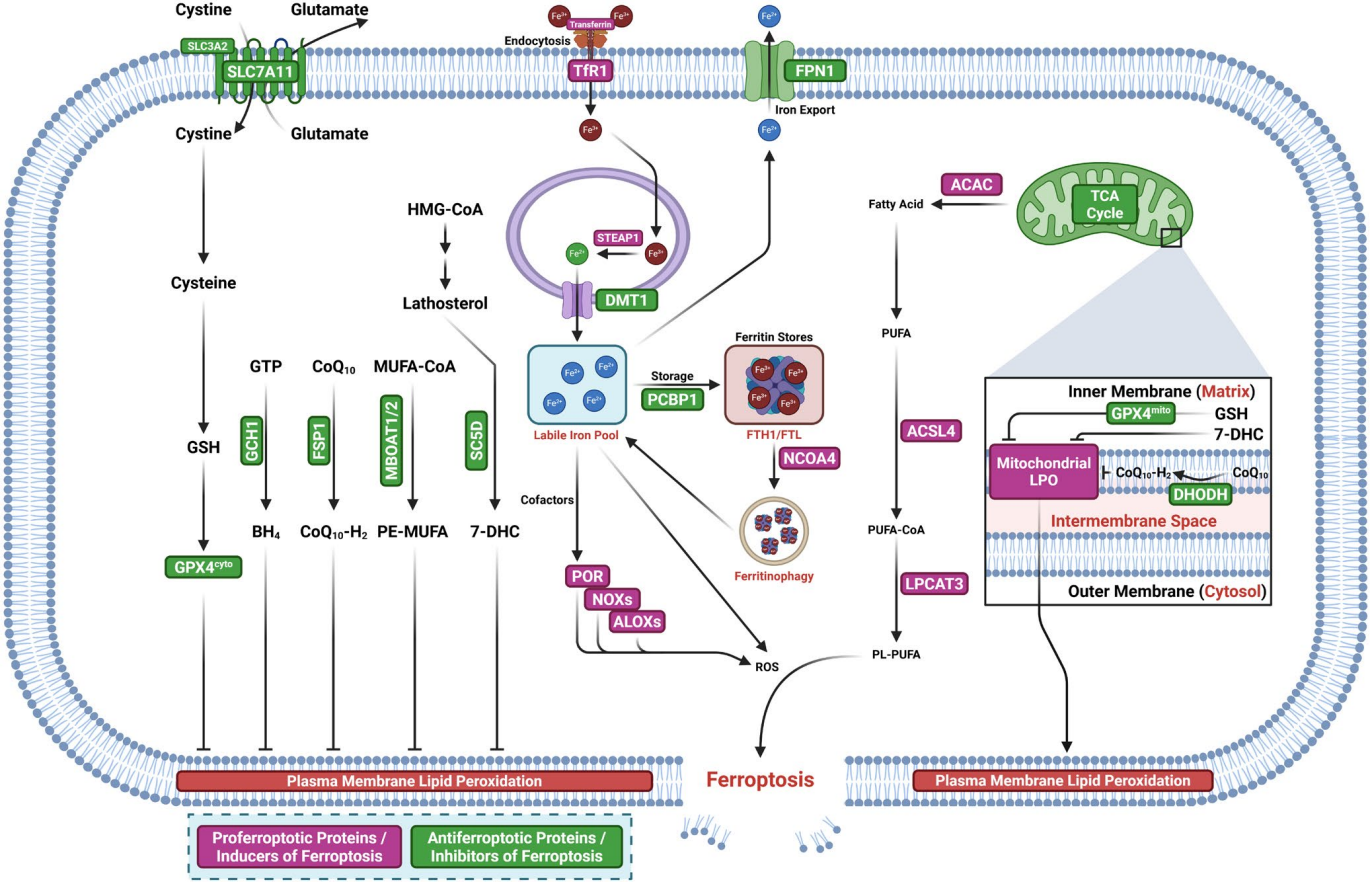
Core mechanisms of ferroptosis. Ferroptosis is induced by an imbalance of pro‐ferroptotic factors and anti‐ferroptotic defence mechanisms. The SLC7A11/SLC3A2 system imports cystine for GSH synthesis to quench lipid peroxides, along with other endogenous antioxidant mechanisms: GCH1, FSP1, MBOAT1/2, and SC5D. Iron import and mobilisation through POR/NOXs/ALOXs catalyses ROS synthesis, which may, alongside the Fenton reaction, induce the synthesis of lipid peroxides. Lipid peroxides accumulate in the plasma membrane and lead to cell lysis.

Ferroptosis is a regulated form of cell death driven by the coordinated disruption of iron homeostasis, lipid peroxidation, and antioxidant defence systems, which together determine cellular susceptibility to oxidative membrane damage. Rather than acting independently, these processes form an integrated regulatory framework in which iron availability fuels lipid oxidation, while antioxidant systems counterbalance this process to control ferroptotic cell fate (Figure 1).

### 2.1 Iron Metabolism and Redox‐Active Iron Pool

Iron metabolism is a central determinant of ferroptosis, as intracellular labile iron promotes lipid peroxidation through redox cycling and reactive oxygen species generation. Early studies demonstrated that ferroptosis requires iron‐dependent metabolic activity, including glutaminolysis and transferrin‐mediated iron uptake, both of which enhance intracellular iron availability and ferroptotic sensitivity [30].

Subsequent work established that intracellular iron storage is dynamically regulated by ferritin and its autophagic degradation. Specifically, ferritinophagy mediated by NCOA4 promotes ferroptosis by releasing stored iron into the cytosol, thereby expanding the labile iron pool and amplifying oxidative stress [31, 32]. This process links autophagic flux to ferroptotic susceptibility and highlights the importance of iron mobilisation in ferroptosis execution.

Conversely, limiting iron availability suppresses ferroptosis by reducing the pool of redox‐active iron. Together, these findings establish intracellular iron homeostasis as a key upstream determinant that modulates the initiation and amplification of ferroptotic signalling.

### 2.2 Lipid Peroxidation and Membrane Vulnerability

Lipid peroxidation represents the execution phase of ferroptosis. The accumulation of peroxidised polyunsaturated fatty acid (PUFA)‐containing phospholipids is a defining biochemical feature that directly drives membrane damage and cell death [33].

The susceptibility of cells to ferroptosis is largely determined by lipid composition. In particular, ACSL4 promotes ferroptosis by facilitating the incorporation of PUFAs into membrane phospholipids, thereby generating substrates that are highly prone to oxidation [34]. These PUFA‐containing phospholipids are subsequently oxidised through iron‐dependent reactions and enzymatic pathways, including lipoxygenase‐mediated processes [35, 36].

Mechanistically, recent studies have identified membrane‐associated oxidoreductases as key drivers of lipid peroxidation. For example, cytochrome P450 oxidoreductase (POR) contributes to ferroptosis by catalysing phospholipid oxidation, while additional oxidoreductases further amplify membrane damage [37, 38]. These findings indicate that ferroptotic membrane injury is not solely a passive consequence of oxidative stress but is actively driven by enzymatic lipid oxidation.

Collectively, lipid metabolic remodelling and enzymatic oxidation determine membrane vulnerability and represent the core execution machinery of ferroptosis.

### 2.3 Antioxidant Defence Systems and Ferroptosis Suppression

Ferroptosis is counterbalanced by multiple antioxidant systems that limit lipid peroxide accumulation. Among these, the glutathione peroxidase 4 (GPX4) pathway is the central protective mechanism. Genetic and pharmacological studies have demonstrated that GPX4 is essential for preventing ferroptosis by reducing lipid hydroperoxides, and its inactivation is sufficient to trigger ferroptotic cell death in vivo and in vitro [39, 40, 41].

The activity of GPX4 depends on intracellular cysteine availability, which is maintained by the system Xc− cystine/glutamate antiporter, thereby linking amino acid metabolism to ferroptosis regulation [42]. Disruption of this pathway reduces glutathione synthesis, impairs GPX4 function, and sensitises cells to ferroptosis.

In addition to GPX4, several GPX4‐independent antioxidant systems have been identified. The FSP1–CoQ10 pathway functions in parallel to GPX4 to suppress ferroptosis by regenerating lipid antioxidants at the plasma membrane [43, 44]. Similarly, the GCH1–BH4 pathway limits ferroptosis through lipid remodelling and redox regulation [45], while mitochondrial DHODH provides an additional defence mechanism by maintaining CoQ‐dependent redox balance [46].

Recent studies further revealed alternative lipid‐protective mechanisms, including sex hormone–regulated MBOAT1/2 pathways and sterol metabolism–dependent suppression of ferroptosis, highlighting the complexity of ferroptosis defense networks [47, 48, 49].

Taken together, ferroptosis is governed by three interdependent processes: iron availability, lipid peroxidation, and antioxidant capacity, which collectively determine cellular susceptibility to oxidative damage (Figure 1). These mechanistic processes provide the molecular basis through which upstream regulatory mechanisms, including epitranscriptomic modifications such as m^6^A, can modulate ferroptotic cell fate.

## 3 m^6^A Regulator Proteins: m^6^A Writers, Erasers and Readers

**FIGURE 2:**
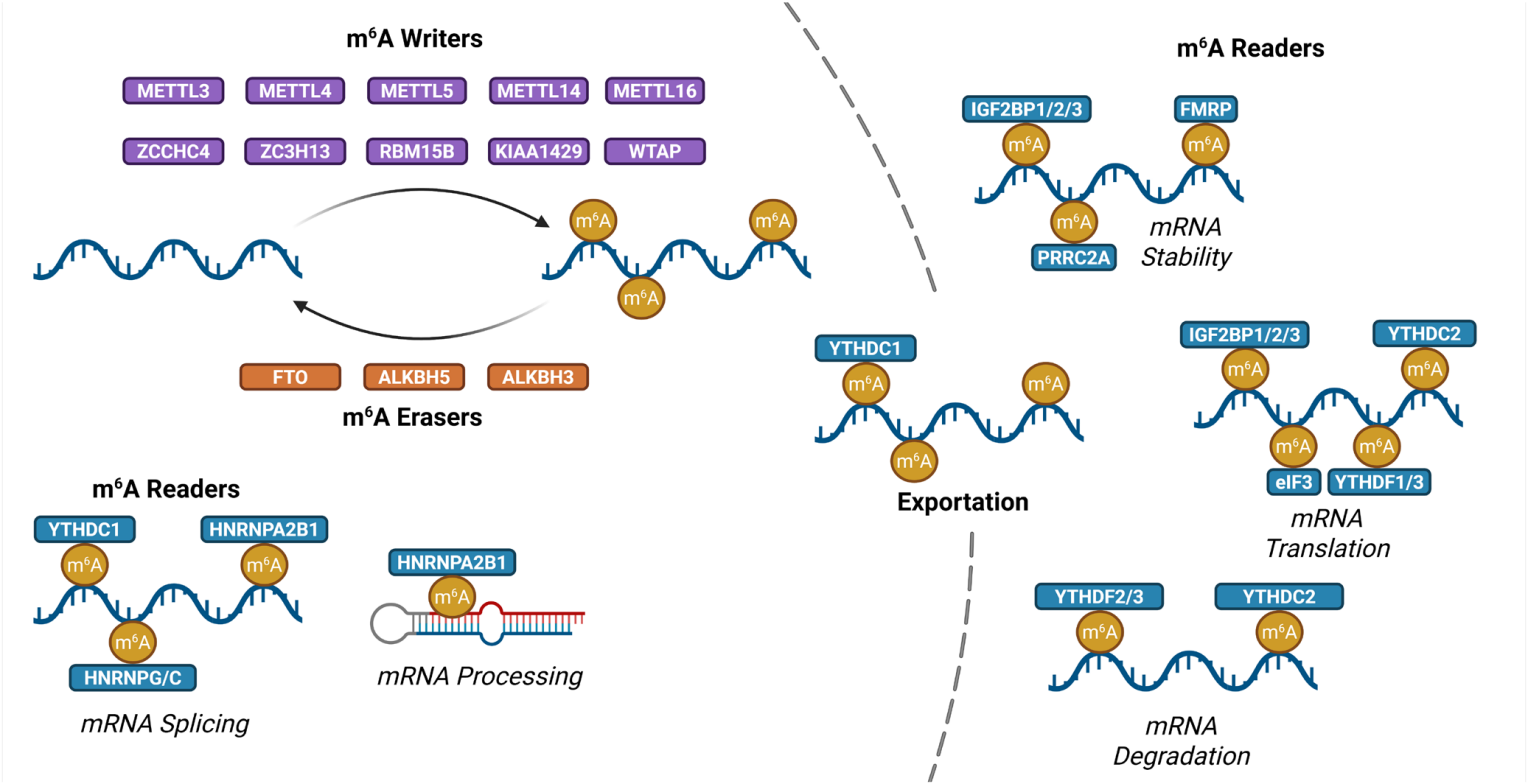
m^6^A regulator proteins and the underlying mechanisms of m^6^A modification. m^6^A modification is primarily driven by the core methylation complex METTL3‐WTAP‐METTL14. Alongside the core complex, RBM15/15B, VIRMA/KIAA1429, and ZC3H13 assist in writing, METTL4 and METTL16 are snRNA modification writers, while METTL5/ZCCHC4 are rRNA m^6^A writers. m^6^A modifications are removed by ALKBH5, ALKBH3, and FTO. m^6^A readers include those of the YTH domain‐containing family, those of the HNRNP family, those of the IGF2BP family, FMPR, PRRC2A, and eIF3.

N6‐methyladenosine (m^6^A) is the most prevalent internal modification in eukaryotic messenger RNA and plays a central role in post‐transcriptional gene regulation. Transcriptome‐wide mapping studies revealed that m^6^A is selectively enriched near stop codons and within 3′ untranslated regions, indicating a regulated distribution across transcripts [50, 51]. Functionally, m^6^A modification modulates multiple aspects of RNA metabolism, including mRNA stability, translation, and decay, through coordinated interactions with RNA‐binding proteins [52, 53]. The m^6^A landscape is dynamically controlled by methyltransferases, demethylases, and m^6^A‐binding proteins, which together determine the fate of modified transcripts (Figure 2).

### 3.1 m^6^A Methyltransferases (‘Writers’)

m^6^A deposition is catalysed by a core methyltransferase complex composed of METTL3 and METTL14, in which METTL3 provides catalytic activity while METTL14 stabilises RNA binding. This functional division was established by biochemical and structural studies demonstrating that the METTL3–METTL14 heterodimer is required for efficient m^6^A installation on target transcripts [54].

Transcriptome‐wide mapping further revealed that m^6^A sites are enriched near stop codons and within 3′ untranslated regions, indicating that m^6^A deposition is selective and position‐dependent rather than stochastic [50, 51].

The activity of the writer complex is regulated by adaptor proteins. For example, WTAP recruits the methyltransferase complex to nuclear speckles, thereby facilitating co‐transcriptional m^6^A deposition [55], whereas VIRMA directs preferential methylation toward the 3′UTR region, shaping the regional distribution of m^6^A marks [56]. In addition, RBM15/15B mediates site‐specific recruitment of the complex to defined RNA regions [57].

Together, these studies demonstrate that m^6^A installation is tightly regulated at both the enzymatic and transcript‐selection levels, enabling context‐dependent modulation of gene expression.

### 3.2 m^6^A Demethylases (‘Erasers’)

The reversibility of m^6^A modification is mediated by Fe(II)/α‐ketoglutarate–dependent dioxygenases. FTO was the first enzyme identified to demethylate m^6^A in nuclear RNA, establishing that RNA methylation is dynamically regulated [58].

Functional studies showed that FTO‐mediated demethylation affects mRNA stability and translation efficiency, thereby modulating gene expression programs linked to metabolism and cellular stress responses [59, 60].

In contrast, ALKBH5 regulates m^6^A levels primarily in the nucleus and influences RNA export and transcript stability, highlighting functional divergence between demethylases [61].

These findings establish that m^6^A removal is not merely a passive process but provides an active mechanism for remodelling RNA fate in response to cellular conditions.

### 3.3 m^6^A‐Binding Proteins (‘Readers’)

The downstream effects of m^6^A are executed by reader proteins that selectively bind methylated RNA. Among these, YTHDF1 enhances translation of m^6^A‐modified transcripts by promoting ribosome loading, thereby increasing protein output [52].

In contrast, YTHDF2 facilitates mRNA decay by recruiting the degradation machinery to m^6^A‐modified transcripts, thereby reducing transcript stability [53]. These complementary functions establish a regulatory balance between translation and degradation.

YTHDF3 can coordinate these processes by interacting with both translation‐promoting and decay‐promoting complexes, thereby fine‐tuning mRNA fate in response to cellular context [62].

In addition to YTH domain proteins, IGF2BP family members recognise m^6^A‐modified transcripts and enhance their stability, thereby promoting sustained gene expression, particularly in metabolic and stress‐related pathways [63].

Further studies have identified additional m^6^A‐binding proteins that regulate RNA processing and localisation, expanding the functional diversity of m^6^A signalling [64, 65, 66, 67, 68].

To facilitate comparison of key m^6^A regulators and their mechanisms, a summary is provided in Table 1. In sum, m^6^A writers, erasers, and readers form a dynamic regulatory system that controls RNA fate at the post‐transcriptional level (Figure 2). By integrating selective methylation, reversible demethylation, and reader‐mediated interpretation, this system enables precise control of gene expression programs, providing a mechanistic basis for how m^6^A modification can influence processes such as ferroptosis.

**TABLE 1:** The function of m^6^A modification regulators in RNA metabolism.

Types	Full names	m^6^A regulator	Cellular localisation	Function	References
Writers	Methyltransferase‐like 3	METTL3	Nucleus	Catalyses m^6^A modification/catalyses methylation reaction.	[50, 51, 54]
	Wilms tumour 1‐associated protein	WTAP	Nucleus	Enhances the localisation of the METTL3‐METTL14 heterodimer within nuclear speckles.	[69, 70]
	Methyltransferase‐like 14	METTL14	Nucleus	Assists METTL3 in identifying the substrate RNA.	[50, 69]
	Vir‐like m^6^A methyltransferase associated	VIRMA (KIAA1429)	Nucleus	Recruits the m^6^A complex to specific RNA regions by binding to polyadenylation cleavage factors CPSF5 and CPSF6.	[71, 72]
	RNA binding motif protein 15	RBM15	Nucleus	Guides the METTL3‐METTL14 heterodimer to targeted RNA sites.	[55, 71]
	RNA binding motif protein 15B	RBM15B	Nucleus	Directs METTL3‐METTL14 heterodimer to specifc RNA sites.	
	Methyltransferase‐like 16	METTL16	Nucleus	Promotes m^6^A modification, specifically influencing the methylation of noncoding RNAs, U6 snRNA, and precursor mRNAs (pre‐mRNAs).	[57, 73, 74]
	Zinc finger CCCH‐type containing 13	ZC3H13	Nucleus	Bridges WTAP to the mRNA‐binding protein Nito, anchoring WTAP in the nucleus and enhancing m^6^A modification efficiency.	[75, 76]
	Methyltransferase‐like 5	METTL5	Nucleus	Enhances m^6^A methylation on 18S rRNA.	[77]
	Zinc finger CCHC‐type containing 4	ZCCHC4	Nucleus	Acts as an m^6^A methyltransferase for 28S rRNA, mediating ribosomal RNA methylation.	[78, 79, 80]
	Methyltransferase‐like 4	METTL4	Nucleus	Promotes the m^6^A methylation of U2 snRNA to modulate pre‐mRNA splicing.	[81]
Erasers	Fat mass and obesity‐associated protein	FTO	Nucleus	Removes m^6^A modification; Works as a m^6^A demethylase to enhance mRNA splicing and translation.	[58]
	AlkB homologue 5	ALKBH5	Nucleus	Removes m^6^A modifcation to enhance mRNA nuclear processing and mRNA export.	[59]
	AlkB homologue 3	ALKBH3	Nucleus	Remove m^6^A modification.	[60]
Readers	YTH N6‐methyladenosine RNA binding protein 2	YTHDF2	Cytosol	Facilitates mRNA degradation.	[82, 83]
	YTH N6‐methyladenosine RNA binding protein 1	YTHDF1	Cytosol	Facilitates mRNA translation initiation.	[84]
	Eukaryotic translation initiation factor 3 subunit A	eIF3	Cytosol	Facilitates mRNA translation.	[85]
	Heterogeneous nuclear ribonucleoprotein A2/B1	HNRNPA2B1	Nucleus	Facilitates mRNA splicing and primary miRNA processing; facilitates primary miRNA processing and mediates nuclear accumulation.	[86]
	Heterogeneous nuclear ribonucleoprotein C	HNRNPC	Nucleus	Facilitates mRNA splicing and maturity; generates a phenomenon termed the ‘m^6^A switch’ by interacting with m^6^A‐modifed mRNA to affect its enrichment and splicing.	[52, 87]
	Heterogeneous nuclear ribonucleoprotein G	HNRNPG	Nucleus	Facilitates mRNA splicing and maturity.	[52, 87]
	YTH domain containing 1	YTHDC1	Nucleus	Regulates RNA nuclear export and splicing; facilitates mRNA splicing and transcriptional silencing.	[53, 62]
	YTH N6‐methyladenosine RNA binding protein 3	YTHDF3	Cytosol	Interacts with YTHDF1 or YTHDF2 to enhance mRNA translation or mRNA degradation, respectively.	[88, 89]
	YTH domain containing 2	YTHDC2	Nucleus; cytosol	Enhances the translation efficiency of target mRNA.	[90]
	Insulin‐like growth factor 2 mRNA binding protein 1	IGF2BP1	Nucleus; cytosol	Facilitates the stability and translation of mRNA.	[91]
	Insulin‐like growth factor 2 mRNA binding protein 2	IGF2BP2	Nucleus; cytosol	Facilitates the stability and translation of mRNA.	[91]
	Insulin‐like growth factor 2 mRNA binding protein 3	IGF2BP3	Nucleus; cytosol	Facilitates the stability and translation of mRNA.	[91]
	Fragile X mental retardation protein	FMRP	Nucleus; cytosol	Facilitates the nuclear export and stability of m^6^A‐modifed RNAs.	[63, 92]
	Proline rich coiled‐coil 2A	PRRC2A	Cytosol	Stabilises Olig2 mRNA by binding to a consensus GGACU motif in the Olig2 coding sequence.	[52]
	RNA‐binding motif protein 33	RBM33	Nucleus	Mediates m^6^A demethylation of selected transcripts by regulating ALKBH5 substrate accessibility and activity through forming a complex with ALKBH5.	[93]

## 4 Epigenetic Regulation of Ferroptosis by m^6^A in Diseases

Recent studies have underscored the key [94, 95] role of epigenetic modifications in disease development and progression through alterations in gene expression and protein homeostasis [96, 97, 98]. These modifications include DNA methylation, histone modification, ncRNA regulation, and m^6^A methylation, collectively regulating gene expression at transcriptional and post‐transcriptional levels [53, 54, 55, 56, 94, 95, 99, 100]. Among them, m^6^A is a reversible and widespread RNA modification that dynamically controls RNA fate [101]. Present in both mRNAs and ncRNAs, m^6^A regulates RNA stability and translation, thereby influencing diverse biological processes, including cancer [101, 102, 103, 104]. Recent evidence indicates that m^6^A modulates ferroptosis‐related genes primarily through post‐transcriptional mechanisms, affecting ferroptosis sensitivity in both cancer and non‐cancer diseases [105]. Notably, m^6^A‐mediated regulation of ferroptosis is highly context‐dependent. Although multiple studies converge on key targets such as SLC7A11, GPX4, ACSL4, and iron metabolism–related genes, the direction of regulation varies across diseases, reflecting differences in oxidative stress and tissue‐specific programs.

### 4.1 Regulating Ferroptosis by m^6^A in Neurological Diseases

**FIGURE 3:**
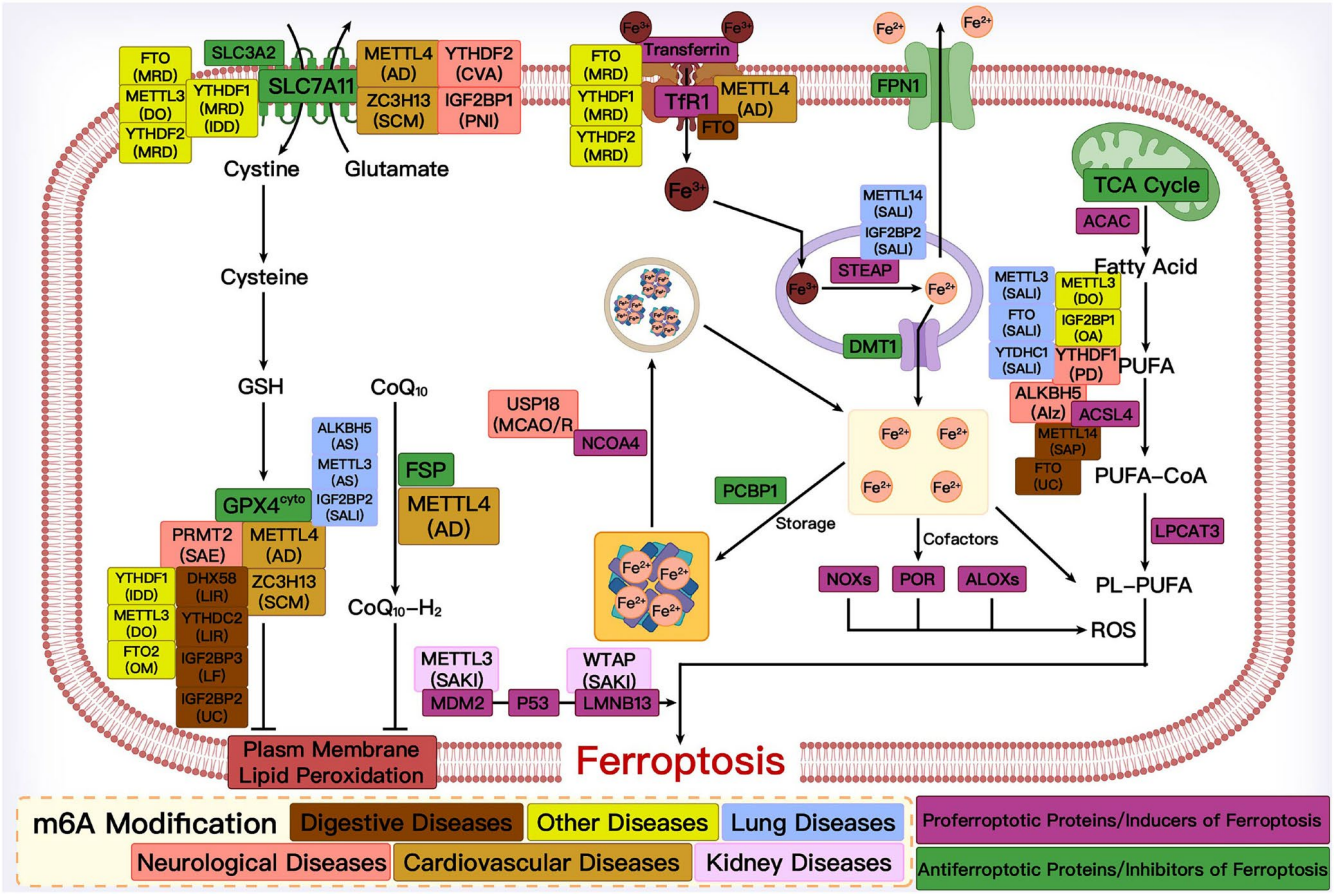
Schematic overview of m^6^A epigenetic modification‐mediated regulation of ferroptosis in multiple human diseases. This diagram illustrates that m^6^A epigenetic modification plays a critical role in the pathogenesis and progression of diverse diseases by targeting key molecules in core ferroptotic pathways (including the cystine/glutamate antiporter system, antioxidant defense, iron metabolism, and lipid peroxidation), thereby reshaping the balance between pro‐ferroptotic and anti‐ferroptotic signalling. The diseases covered in this schematic include neurological diseases, cardiovascular diseases, digestive system diseases, lung diseases, kidney diseases, other systemic diseases, and malignant tumours.

Ferroptosis contributes to neuronal injury across neurological disorders, where high oxidative stress and lipid‐rich membranes increase susceptibility to lipid peroxidation. In this setting, m^6^A regulates ferroptosis mainly through antioxidant defence, iron metabolism, and RNA processing, with disease‐specific variations (Figure 3 and Table 2).

**TABLE 2:** m^6^A modification of ferroptosis in neurological diseases.

Diseases	Writers	Erasers	Readers	Ferroptosis targets	Effect on ferroptosis	Biological functions	References
AD	—	ALKBH5	hnRNPM	HO‐1	↓	ALKBH5 deficiency may worsen cobalt‐induced ferroptosis by boosting lipid peroxidation through the hnRNPM‐dependent downregulation of HO‐1. Consequently, APP production increases, and the activity of its hydrolases, BACE1 and PSEN1, becomes altered.	[106]
AD	METTL14	—	—	Nrf2	↓	METTL14 ameliorates AD pathological development by inhibiting ferroptosis through m^6^A modification of TUG1 to activate GDF15/Nrf2 axis.	[107]
Parkinson's disease	—	FTO	—	Nrf2	↑	FTO promotess MPP+–induced ferroptosis by demethylating the m^6^A modification from Nrf2 mRNA, impairing the Nrf2 mRNA stability in SH‐SY5Y cells.	[108]
Parkinson's disease	METTL3		YTHDF1	ACSL4	↑	Soot nanoparticles exacerbates development of PD by inducing ferroptosis via upregulating ACSL4 expression through METTL3/YTHDF1‐mediated m^6^A modulation.	[109]
Ischemic stroke	METTL3	—	—	TFRC	↓	Overexpression of METTL3 alleviates brain ischemic injury by inhibiting oxidative damage and ferroptosis through inhibiting TFRC by upregulating the E3 ubiquitin ligase NEDD4L.	[110]
Ischemic stroke	—	FTO	—	FYN	↓	FTO alleviates MCAO/R cerebral injury by decreasing ferroptosis through downregulating FYN expression through m^6^A modification, thereby subduing Drp1 activity.	[111]
Ischemic stroke	—	—	YTHDF1	BECN1	↑	HIF‐1α‐mediated upregulation of YTHDF1 promotes ischemic stroke by inducing ferroptosis through by enhancing the stability of BECN1 mRNA.	[112]
Ischemic stroke	—	FTO	—	NCOA4	↓	USP18‐mediated stabilisation of FTO suppresses ferritinophagy‐mediated ferroptosis to alleviate cerebral I/R injury by removing NCOA4 m^6^A modification to inhibit NCOA4 translation.	[113]
Ischemic stroke	—	FTO	—	SLC7A11	↓	Decreased FTO promotes maturation of miR‐320‐3p, which increased miR‐320‐3p enhances cerebral ischemia/reperfusion injury by promoting ferroptosis through inhibiting SLC7A11.	[114]
Cerebrovascular atherosclerosis	—	—	YTHDF2	SLC7A11	↑	YTHDF2 cerebrovascular atherosclerosis by facilitating endothelial cells ferroptosis through binding to promote degradation of SLC7A11 mRNA via m^6^A‐dependent manner.	[115]
ICH	METTL3	—	—	TFRC	↑	Increased METTL3 and m^6^A levels were observed in hemin‐treated PC12 cells. Silencing METTL3 enhanced cell viability and suppressed ferroptosis in these cells. However, this protective effect was reversed by TFRC overexpression. Hemin‐induced lactylation stabilised METTL3 protein, elevating m^6^A levels and TFRC mRNA expression, ultimately promoting ferroptosis in PC12 cells.	[116]
SAE	—	ALKBH5	—	GPX4	↑	ALKBH5 upregulation of PRMT2 in SAE facilitated ferroptosis by promoting methylation‐mediated β‐catenin degradation, subsequently suppressing GPX4 expression and exacerbating ferroptosis.	[117]
HIE	—	FTO	—	FTH1	↓	FTO reduced neonatal HIE‐related damage by attenuating ferroptosis through inhibition of FTH1.	[118]
PNI	—	—	IGF2BP1	SLC7A11	↓	HIF‐1α overexpression in DRG neurons elevated overall m^6^A methylation levels by upregulating IGF2BP1. This process increased the expression and stability of neurotrophic factors (NGF, BDNF, and GDNF) and SLC7A11 mRNA, which could be reversed by silencing IGF2BP1.	[119]
Bilirubin‐induced brain damage	—	ALKBH5	—	ACSL4	↑	ALKBH5 contributed to hyperbilirubinemia‐induced ferroptosis by stabilising ACSL4 mRNA via m^6^A modification, further enhancing ferroptosis.	[120]

Abbreviations: BDNF, brain‐derived neurotrophic factor; GDNF, glial‐derived neurotrophic factor; HO‐1, heme oxygenase‐1; ICH, intracerebral haemorrhage; NGF, nerve growth factor; PNI, peripheral nerve injury; SAE, sepsis‐associated encephalopathy.

#### 4.1.1 Alzheimer's Disease (AD)

Research has demonstrated that cobalt induces ferroptosis in H4 cells, as indicated by reduced expression of SLC7A11, GPX4, and FSP1, alongside elevated levels of ACSL4, HO‐1, and transferrin receptor protein 1 (TFRC) [106]. These molecular changes reflect coordinated disruption of antioxidant systems and iron metabolism, consistent with activation of ferroptotic pathways. Cobalt exposure also upregulates the expression of key Alzheimer's‐related proteins, including APP, BACE1, and PSEN1. These changes are reversed by ferroptosis inhibitors, implicating ferroptosis in cobalt‐induced neurodegenerative processes [106].

Cobalt induces ferroptosis in H4 cells, characterised by reduced SLC7A11, GPX4, and FSP1, and increased ACSL4, HO‐1, and TFRC, alongside elevated APP, BACE1, and PSEN1 levels [106]. These effects are reversed by ferroptosis inhibitors, indicating a ferroptosis‐dependent neurodegenerative process.

Cobalt reduces ALKBH5 expression and impairs its interaction with HO‐1 mRNA, identifying HO‐1 as a downstream target. ALKBH5 silencing increases lipid peroxidation, suppresses HO‐1, and enhances APP, BACE1, and PSEN1 expression, thereby aggravating neurodegeneration [106]. This suggests that ALKBH5 limits ferroptosis by maintaining HO‐1–mediated antioxidant capacity.

hnRNPM binds HO‐1 mRNA and regulates its splicing. Silencing hnRNPM reduces HO‐1 expression and enhances ferroptosis, while co‐silencing ALKBH5 further amplifies these effects, indicating that m^6^A‐dependent regulation of HO‐1 splicing links RNA processing to ferroptosis [106].

METTL14 is decreased, whereas lncRNA TUG1 is increased in AD models [107]. METTL14 overexpression or TUG1 silencing suppresses ferroptosis and neurotoxicity. Mechanistically, METTL14 reduces TUG1 stability, while TUG1 promotes SMURF1‐mediated GDF15 degradation and suppresses Nrf2 signalling [107]. Loss of GDF15 abolishes the protective effects, whereas METTL14 overexpression restores antioxidant signalling and improves cognitive function.

Together, these findings show that METTL14 suppresses ferroptosis via the TUG1/GDF15/Nrf2 signalling pathway, whereas ALKBH5 limits ferroptosis through HO‐1 regulation, indicating regulator‐specific mechanisms converging on oxidative stress control. Compared with other systems, m^6^A regulation in AD is more closely linked to antioxidant signalling and RNA processing rather than direct lipid metabolic regulation.

#### 4.1.2 Parkinson's Disease

In Parkinson's disease models, reduced m^6^A modification and increased FTO expression have been observed in MPP+–treated SH‐SY5Y cells and MPTP‐treated mice [108]. Silencing FTO suppresses ferroptosis, indicating that FTO promotes ferroptotic susceptibility. Mechanistically, FTO demethylates Nrf2 mRNA, reducing its stability and weakening antioxidant defence, thereby facilitating ferroptosis [108].

In addition to antioxidant regulation, m^6^A also influences lipid metabolism in PD. Soot nanoparticles induce ferroptosis by increasing ACSL4 expression through METTL3‐dependent m^6^A modification and YTHDF1‐mediated translation enhancement in dopaminergic neurons [109].

Together, these findings indicate that m^6^A regulates ferroptosis in PD through two principal mechanisms: destabilisation of antioxidant pathways via FTO–Nrf2 regulation and enhancement of lipid peroxidation through METTL3/YTHDF1‐dependent ACSL4 upregulation. This dual regulation highlights a disease‐specific pattern in which both oxidative defense and lipid metabolism are coordinately controlled.

#### 4.1.3 Ischemic Stroke

Ferroptosis is a key contributor to ischemic stroke pathology [9, 121, 122, 123], and multiple studies demonstrate that m^6^A regulates ferroptosis through diverse molecular pathways. Reduced METTL3 expression has been observed in MCAO models, while METTL3 overexpression alleviates ischemic injury by suppressing oxidative stress and ferroptosis [110]. Mechanistically, METTL3 stabilises NEDD4L mRNA via m^6^A modification, leading to TFRC degradation, reduced iron accumulation, and decreased ferroptotic activity [110].

FTO also plays a protective role in ischemic stroke. In tMCAO/R and OGD/R models, reduced FTO expression is associated with increased FYN levels and enhanced ferroptosis [111]. FTO overexpression suppresses ferroptosis by inhibiting FYN expression, thereby reducing Drp1‐mediated mitochondrial fission and oxidative stress [111].

m^6^A readers further contribute to ferroptosis regulation in ischemic injury. YTHDF1 promotes ferroptosis by stabilising BECN1 mRNA, and its expression is transcriptionally induced by HIF‐1α, linking hypoxic signalling to ferroptotic regulation [112].

Additional regulatory layers involve RNA stability and ferritinophagy. USP18 stabilises FTO, reducing m^6^A modification of NCOA4 mRNA and suppressing its translation, thereby limiting ferritinophagy and ferroptosis [124]. In parallel, reduced FTO enhances miR‐320‐3p maturation, which suppresses SLC7A11 and promotes ferroptosis, further aggravating ischemic injury [114].

Together, these findings demonstrate that m^6^A regulates ferroptosis in ischemic stroke through multiple interconnected mechanisms, including iron metabolism (TFRC, NCOA4), antioxidant defence (SLC7A11), mitochondrial dynamics (FYN/Drp1), and autophagy‐related pathways (BECN1). Compared with neurodegenerative diseases, ischemic stroke exhibits a broader and more heterogeneous regulatory network involving both transcriptional and post‐transcriptional processes.

##### 4.1.3.1 Cerebrovascular Atherosclerosis

In ox‐LDL‐treated endothelial cells, YTHDF2 expression is upregulated and promotes ferroptosis by binding to and degrading SLC7A11 mRNA in an m^6^A‐dependent manner [115]. Silencing YTHDF2 reduces ferroptosis and improves cell viability, indicating that m^6^A‐mediated RNA decay contributes to endothelial dysfunction in vascular pathology.

##### 4.1.3.2 Intracerebral Haemorrhage (ICH)

In hemin‐treated PC12 cells, increased lactylation stabilises METTL3 and enhances its expression, leading to elevated m^6^A levels and increased TFRC mRNA expression [116]. Silencing METTL3 suppresses ferroptosis, indicating that METTL3 promotes ferroptosis in ICH by enhancing iron uptake through TFRC regulation.

##### 4.1.3.3 Sepsis‐Associated Encephalopathy (SAE)

In LPS‐induced models, PRMT2 expression is elevated while ALKBH5 expression is reduced [117]. ALKBH5‐mediated m^6^A modification stabilises PRMT2 mRNA, promoting arginine methylation and proteasomal degradation of β‐catenin. This suppresses GPX4 expression and enhances ferroptosis [117]. Loss of PRMT2 reduces ferroptosis and improves behavioural outcomes, indicating that ALKBH5‐dependent PRMT2 regulation contributes to ferroptosis and neuroinflammation in SAE.

##### 4.1.3.4 Neonatal Hypoxic–Ischemic Brain Injury (HIE)

Reduced FTO expression has been observed in neonatal HIE patients and in OGD‐treated neurons, and is further decreased upon exposure to ferroptosis inducers such as FAC [118]. Restoration of FTO suppresses ferroptosis, indicating a protective role. Mechanistically, FTO regulates m^6^A modification of FTH1 mRNA, reducing its stability and protein expression. Reintroduction of FTH1 abolishes the anti‐ferroptotic effect of FTO, demonstrating that FTO limits ferroptosis through modulation of iron storage. These findings indicate that m^6^A‐dependent control of ferritin metabolism is a central mechanism in HIE, distinguishing it from other neurological disorders that primarily involve antioxidant or lipid metabolic pathways [118].

##### 4.1.3.5 Peripheral Nerve Injury (PNI)

Following peripheral nerve injury, hypoxia‐induced HIF‐1α upregulates IGF2BP1, which enhances m^6^A‐dependent stabilisation of SLC7A11 mRNA in dorsal root ganglion neurons [119]. This leads to increased antioxidant capacity, reduced ferroptosis, and improved neuronal recovery. In contrast to HIE, where m^6^A regulates iron storage, PNI is characterised by m^6^A‐mediated stabilisation of cystine transport and redox balance, highlighting a distinct regulatory mechanism centred on antioxidant defense [119].

##### 4.1.3.6 Bilirubin‐Induced Brain Damage

Ferroptosis contributes to bilirubin‐induced neurotoxicity, where increased m^6^A modification and reduced ALKBH5 expression have been observed in UCB‐treated neurons [125]. ALKBH5 suppresses ferroptosis, and its loss enhances oxidative damage. Mechanistically, ACSL4 is a downstream target of ALKBH5, and m^6^A‐dependent regulation stabilises ACSL4 mRNA, promoting lipid peroxidation and ferroptosis [120]. Genetic or pharmacological inhibition of ACSL4 reverses these effects, confirming its central role. These findings indicate that, unlike HIE and PNI, m^6^A regulation in bilirubin‐induced brain injury primarily targets lipid metabolic pathways, emphasising enhanced membrane lipid peroxidation as the dominant mechanism [120].

### 4.2 Regulating Ferroptosis by m^6^A in Cardiovascular Diseases

Ferroptosis contributes to cardiovascular injury through oxidative stress, iron dysregulation, and lipid peroxidation (Figure 3 and Table 3) [127, 128, 129, 130, 131, 132, 142]. m^6^A modification regulates these processes by controlling RNA stability and translation of ferroptosis‐related genes [127, 128, 129, 130, 131, 132, 142, 143]. However, the dominant regulatory mechanisms differ across cardiovascular diseases, with distinct m^6^A targets engaged in different pathological contexts [127, 128, 129, 130, 131, 132, 134, 135, 136, 137, 138, 139, 142, 143]. In ischemic injury, m^6^A‐dependent ferroptosis is mainly linked to lipid peroxidation and iron metabolism [127, 128, 129, 142]. In metabolic cardiomyopathy, regulation is more closely associated with epigenetic activation of ferroptosis‐related genes [130]. In inflammatory cardiac injury, m^6^A primarily affects antioxidant defense pathways [134, 135, 136]. In vascular disorders, m^6^A can either suppress or promote ferroptosis depending on the specific transcripts involved [138, 139].

**TABLE 3:** m^6^A modification of ferroptosis in cardiovascular diseases.

Diseases	Writers	Erasers	Readers	Ferroptosis targets	Effect on ferroptosis	Biological functions	References
AMI	ZC3H13	—	—	ACSL4; PTGS2; GPX4	↓	ZC3H13 suppresses AMI by inhibiting ferroptosis through targeting to downregulate lncRNA93358 in m^6^A dependant manner	[126]
AMI	WTAP	—	—	ACSL4; FTH1; GPX4	↑	WTAP facilitates hypoxia/reoxygenation‐induced human cardiomyocyte injury by inducing ferroptosis through decreasing KLF6 in m^6^A modification manner	[127]
AMI	METTL14	—	—	ACSL4; GPX4	↑	METTL14 facilitates hypoxia/reoxygenation (H/R)‐induced ferroptosis by enhancing the expression of miR‐146a‐5p. This occurs through METTL14‐mediated promotion of DGCR8's recognition and processing of pri‐miR‐146a‐5p. Subsequently, miR‐146a‐5p inhibits APPL1 transcription, contributing to the induction of ferroptosis under H/R conditions.	[128]
AMI	METTL3	—	IGF2BP2	ACSL4	↑	IGF2BP2 recognised METTL3‐mediated increased KLF6 promotes myocardial ischemia/reperfusion injury by activating ACSL4‐mediated ferroptosis.	[129]
Diabetic cardiomyopathy	—	ALKBH5	YTHDF2	TFRC; HO‐1	↑	ALKBH5‐mediated upregulation of KAT2a promotes DCM by inducing ferroptosis through epigenetic activation of TFRC and HMOX1 transcription	[130]
Doxorubicin‐induced cardiotoxicity	—	FTO	—	P53; Nrf2	↓	FTO reduces doxorubicin (DOX)‐induced cardiotoxicity by preventing ferroptosis through the activation of the p21/Nrf2 axis, either with or without p53 involvement. This regulation is achieved via FTO‐mediated m^6^A demethylation. HuR plays an essential role in supporting FTO's ferroptosis‐inhibiting effects by stabilising the p53‐p21/Nrf2 pathway. Furthermore, FTO establishes a positive feedback loop with HuR and the p53‐p21/Nrf2 axis, amplifying its cardioprotective effects and reinforcing the suppression of ferroptosis in DOX‐induced cardiac injury.	[131]
Doxorubicin‐induced cardiotoxicity	METTL3	—	IGF2BP2	TFRC	↑	METTL3 contributes to DOX‐induced cardiotoxicity by inducing ferroptosis through TFRC m^6^A modification in IGF2BP2 dependant manner.	[132]
Doxorubicin‐induced cardiotoxicity	METTL14	—	IGF2BP1	TFRC	↑	METTL14 facilitates DOX‐induced ferroptosis by upregulating TFRC through increasing lncRNA KCNQ1OT1 to absorbs miR‐7‐5p, which works as a ceRNA and increases the mRNA level of TFRC.	[133]
Septic cardiomyopathy	ZC3H13	—	—	SLC7A11; GPX4	↑	ZC3H13 promotes sepsis‐induced cardiomyopathy by inducing ferroptosis through downregulating Pnn, GPX4, and SLC7A11 and upregulating Rbm25 and Caspase 3.	[134]
Septic cardiomyopathy	—	FTO	—	BACH1	↓	FTO alleviates ferroptosis in septic cardiomyopathy via mediating the m^6^A modification of BACH1.	[135]
Septic cardiomyopathy	METTL3	—	—	SLC7A11	↑	METTL3 promotes SCM by inducing ferroptosis through increased m^6^A methylation on SLC7A11 and ensued mRNA degradation in YTHDF2‐dependent manner	[136]
Septic cardiomyopathy	METTL14	—	—	TRPM7	↑	METTL14 promotes SCM by inducing ferroptosis via increase the stability of TRPM7 mRNA via m^6^A methylation.	[137]
Aortic dissection	METTL3	—	YTHDF2	HUR/GPX4	↓	METTL3‐mediated lncRNA NORAD alleviates aortic dissection by suppressing ferroptosis of VSMCs via the HUR/GPX4 axis, highlighting lncRNA NORAD as an aortic dissection therapeutic target	[138]
Aortic dissection	METTL3	—	—	SLC7A11; FSP1	↑	In patients with thoracic aortic aneurysm and dissection (TAAD), high METTL3 levels in the aorta promote ferroptosis in human aortic smooth muscle cells (HASMCs) through enhancing the mRNA degradation of SLC7A11 and FSP1, lowering their protein levels.	[139]
Aortic dissection	METTL14	—	IGF2BP2	ACSL4	↑	METTL14 enhances ferroptosis in smooth muscle cells during thoracic aortic aneurysm by stabilising the m^6^A modification of ACSL4.	[140]
Cardiac and vascular toxicity	—	—	YTHDF2	GCH1	↓	Cardiomyocyte ferroptosis is triggered by the suppression of YTHDF2‐mediated translation of m^6^A‐modified GCH1, further contributing to ferroptotic pathways in cardiovascular pathology.	[141]

Abbreviations: AMI, acute myocardial infarction; DOX, doxorubicin; PTGS2, prostaglandin‐endoperoxide synthase 2; SCM, septic cardiomyopathy.

#### 4.2.1 Acute Myocardial Infarction (AMI)

In AMI and ischemia/reperfusion models, multiple m^6^A regulators modulate ferroptosis through distinct mechanisms. ZC3H13 suppresses ferroptosis by downregulating lncRNA93358, leading to reduced ACSL4 and PTGS2 and increased GPX4, thereby limiting myocardial injury [142]. In contrast, WTAP promotes ferroptosis by stabilising KLF6 mRNA through m^6^A modification, enhancing cardiomyocyte injury under hypoxia/reoxygenation [127]. METTL14 facilitates DGCR8‐dependent processing of pri‐miR‐146a‐5p, increasing miR‐146a‐5p and suppressing APPL1, which promotes ferroptosis [128]. METTL3, together with IGF2BP2, stabilises KLF6 and enhances ACSL4 transcription, further driving lipid peroxidation and ferroptosis [129].

These findings indicate that m^6^A regulates ferroptosis in AMI through coordinated control of lncRNA expression, miRNA maturation, and transcriptional activation of lipid metabolism genes, with ACSL4‐mediated lipid peroxidation as a central downstream outcome [127, 128, 129, 142].

#### 4.2.2 Diabetic Cardiomyopathy (DCM)

In DCM, m^6^A‐mediated ferroptosis is linked to transcriptional activation of iron metabolism and oxidative stress genes. ALKBH5 reduces m^6^A modification of KAT2a mRNA, while decreased YTHDF2 limits its degradation, resulting in increased KAT2a expression [130]. KAT2a promotes H3K27ac enrichment at TFRC and HMOX1 loci, activating their transcription and enhancing ferroptosis. This mechanism differs from AMI, as it is primarily driven by epigenetic activation rather than direct regulation of lipid peroxidation pathways [130].

#### 4.2.3 Doxorubicin‐Induced Cardiotoxicity

In DOX‐induced cardiotoxicity, m^6^A regulators exert opposing effects on ferroptosis. FTO suppresses ferroptosis by activating the p21/Nrf2 pathway through m^6^A demethylation, enhancing antioxidant capacity and improving cardiac function [131]. In contrast, METTL3 promotes ferroptosis by increasing m^6^A modification of TFRC mRNA and enhancing its stability through IGF2BP2, leading to iron accumulation [132]. METTL14 also promotes ferroptosis by stabilising lncRNA KCNQ1OT1, which relieves miR‐7‐5p‐mediated repression of TFRC and increases ferroptotic susceptibility [143].

These findings indicate that ferroptosis in DOX‐induced injury is determined by the balance between antioxidant regulation and iron metabolism, both of which are controlled by m^6^A‐dependent mechanisms [131, 132, 143].

#### 4.2.4 Septic Cardiomyopathy (SCM)

In septic cardiomyopathy, m^6^A‐mediated ferroptosis is closely associated with suppression of antioxidant defence. ZC3H13 promotes ferroptosis by reducing GPX4 and SLC7A11 and increasing pro‐apoptotic signalling [134]. FTO attenuates ferroptosis through regulation of BACH1 and improves cardiac function and survival [135]. METTL3 promotes ferroptosis by enhancing m^6^A‐dependent degradation of SLC7A11 via YTHDF2, further impairing antioxidant capacity [136].

These results indicate that m^6^A regulation in SCM primarily targets antioxidant systems rather than lipid metabolism or iron storage pathways [134, 135, 136].

#### 4.2.5 Aortic Dissection

In aortic dissection, m^6^A‐dependent ferroptosis exhibits both protective and pathogenic regulation. lncRNA NORAD suppresses ferroptosis by stabilising GPX4 mRNA through interaction with HUR, and its stability is supported by METTL3‐mediated m^6^A modification [138]. In contrast, increased METTL3 expression reduces SLC7A11 and FSP1 levels, promoting ferroptosis in vascular smooth muscle cells [139].

These findings indicate that m^6^A regulates vascular ferroptosis through distinct mechanisms depending on the targeted transcript, including preservation of GPX4 or suppression of SLC7A11 and FSP1 [138, 139].

#### 4.2.6 Cardiac and Vascular Toxicity

Environmental toxicants can also induce m^6^A‐dependent ferroptosis. Fluorene‐9‐bisphenol suppresses YTHDF2 expression and disrupts m^6^A‐dependent translation of GCH1, leading to ferroptosis in cardiomyocytes [141]. This suggests that environmental stressors can trigger ferroptotic injury by interfering with m^6^A‐regulated redox pathways [141].

### 4.3 Regulating Ferroptosis by m^6^A in Digestive System Disease

Ferroptosis contributes to digestive system disorders through oxidative stress, iron metabolism imbalance, and lipid peroxidation, and m^6^A modification regulates these processes in a context‐dependent manner (Figure 3 and Table 4) [144, 145, 147, 148, 149, 150, 151, 152]. In ischemic liver injury, m^6^A primarily affects antioxidant defense, whereas in fibrosis and pancreatitis it is more closely linked to autophagy and lipid metabolism, and in inflammatory bowel disease it mainly regulates GPX4‐dependent redox homeostasis [144, 145, 147, 148, 149, 150, 151, 152].

**TABLE 4:** m^6^A modification of ferroptosis in digestive system disease.

Diseases	Writers	Erasers	Readers	Ferroptosis targets	Effect on ferroptosis	Biological functions	References
Liver IRI	METTL3	—	YTHDC2	GPX4	↓	DHX58 protects against liver I/R injury by inhibiting ferroptosis via recruiting YTHDC2 to enhance translation of modifed‐GPX4 mRNA, thus enhancing GPX4 protein levels.	[144]
Liver IRI	—	FTO	—	ACSL4; TFRC	↓	FTO loss in older livers enhances ferroptosis by upregulating ACSL4 and TFRC during IR injury.	[145]
Liver fibrosis	METTL4	FTO	YTHDF1	ACSL4; PTGS2; GPX4	↓	Increased METTL4 and decreased FTO levels stabilise BECN1 mRNA, which helps YTHDF1 trigger autophagy. This process eventually leads to HSC ferroptosis.	[146]
Liver fibrosis	—	—	IGF2BP3	GPX4	↓	HSC‐specific loss of IGF2BP3 attenuates liver fibrosis by inducing HSC ferroptosis through downregulating GPX4 via recognising Jag1 m^6^A binding sites to inhibit Notch and inactivate HES1.	[147]
Liver fibrosis			YTHDF2	ACSL4		YTHDF2 reduces fibrosis by inducing HSC ferroptosis through activating ACSL4 in m^6^A modification manner.	[148]
Severe acute pancreatitis	METTL14	—	IGF2BP2	SAT1; ACSL4	↑	METTL14 promotes SAP by inducing ferroptosis through increasing the N6‐methyladenosine modification of ACSL4 and STA1.	[149]
Severe acute pancreatitis	METTL3	—	—	BACH1/HSPB1	↑	METTL3‐mediated stability of BACH1 promotes severe acute pancreatitis by inducing ferroptosis by suppressing HSPB1.	[150]
Ulcerative colitis	—	—	IGF2BP2	GPX4	↓	IGF2BP2 suppresses UC progression by inhibiting ferroptosis via augmenting the GPX4 expression by the m^6^A modification.	[151]

Abbreviations: DHX58, DExH‐box helicase 58; HSC, hepatic stellate cells; I/R, liver ischemia/reperfusion (I/R) injury; PTGS2, prostaglandin‐endoperoxide synthase 2; TFRC, transferrin receptor.

#### 4.3.1 Liver Ischemia/Reperfusion (I/R) Injury

In hepatic I/R injury, m^6^A regulation of ferroptosis is centred on GPX4‐dependent antioxidant defense. DHX58 expression is reduced during I/R injury, leading to increased ROS and ferroptosis [144]. DHX58 binds GPX4 mRNA and, together with YTHDC2, enhances its translation in an m^6^A‐dependent manner, whereas METTL3 silencing reduces m^6^A modification and GPX4 protein levels [144]. This indicates that DHX58 protects against ferroptosis by promoting m^6^A‐dependent translation of GPX4.

In aged livers, reduced FTO expression further enhances ferroptosis during I/R injury by increasing ACSL4 and TFRC levels through m^6^A‐dependent mRNA stabilisation [145]. These findings indicate that hepatic I/R injury is mainly regulated by m^6^A‐dependent control of antioxidant capacity and iron metabolism [144, 145].

#### 4.3.2 Liver Fibrosis

In liver fibrosis, m^6^A‐mediated ferroptosis is closely associated with activation of hepatic stellate cells (HSCs) and autophagy. Increased m^6^A modification, driven by METTL4 upregulation and FTO downregulation, stabilises BECN1 mRNA through YTHDF1, promoting autophagy and ferroptosis in HSCs [152]. Induction of ferroptosis reduces fibrosis, whereas inhibition of m^6^A modification suppresses this effect [152].

In addition, IGF2BP3 regulates ferroptosis through GPX4‐dependent mechanisms. Loss of IGF2BP3 reduces m^6^A modification of Jag1, suppresses HES1 transcription, and decreases GPX4 expression, thereby inducing ferroptosis and attenuating fibrosis [147].

These findings indicate that m^6^A‐dependent ferroptosis in liver fibrosis is primarily linked to autophagy activation and transcriptional regulation of GPX4, distinguishing it from I/R injury where GPX4 translation and iron metabolism are dominant [147, 152, 153].

#### 4.3.3 Severe Acute Pancreatitis (SAP)

In SAP, m^6^A regulation of ferroptosis is driven mainly by lipid metabolism and stress‐response pathways. METTL14 expression is increased and promotes ferroptosis by enhancing m^6^A modification of ACSL4 and SAT1 mRNAs, which are stabilised by IGF2BP2 [149].

In parallel, METTL3 promotes ferroptosis by stabilising BACH1 mRNA, which suppresses HSPB1 expression and weakens cellular stress resistance [150].

These findings indicate that ferroptosis in SAP is regulated through both lipid peroxidation pathways and stress‐response signalling, with ACSL4 and BACH1 as key downstream effectors [149, 150].

#### 4.3.4 Ulcerative Colitis (UC)

In ulcerative colitis, m^6^A‐mediated ferroptosis is primarily associated with GPX4‐dependent antioxidant defence. IGF2BP2 expression is reduced in DSS‐induced models, leading to decreased stability of m^6^A‐modified GPX4 mRNA and enhanced ferroptosis [151]. Restoration of IGF2BP2 stabilises GPX4 expression, suppresses ferroptosis, and alleviates disease severity [151].

This pattern differs from SAP and liver fibrosis, as m^6^A regulation in UC is mainly centred on maintenance of antioxidant capacity rather than lipid metabolism or autophagy [151].

### 4.4 Regulating Ferroptosis by m^6^A in Lung Diseases

Ferroptosis contributes to respiratory diseases through oxidative stress, lipid peroxidation, and iron metabolism, and m^6^A modification regulates these processes in a disease‐dependent manner (Figure 3 and Table 5) [154, 155, 156, 158, 159, 161, 162, 163, 165, 166]. In sepsis‐associated lung injury, m^6^A primarily enhances ferroptosis through lipid metabolism and inflammatory signalling. In asthma, regulation is centred on GPX4‐dependent antioxidant defence. In chronic obstructive pulmonary disease, m^6^A mainly affects iron metabolism through translational control [154, 155, 156, 158, 159, 161, 162, 163, 165, 166].

**TABLE 5:** m^6^A modification of ferroptosis in lung disease.

Diseases	Writers	Erasers	Readers	Ferroptosis targets	Effect on ferroptosis	Biological functions	References
Sepsis‐associated lung injury	METTL3	—	—	ACSL4	↑	Lactate worsens sepsis‐related lung injury by triggering ferroptosis. It does this by promoting ACSL4 through GPR81‐mediated upregulation of METTL3 in alveolar epithelial cells.	[154]
Sepsis‐associated lung injury	METTL3	—	IGF2BP2	—	↑	NETs upregulates p300, which promotes H3K27ac‐mediated METTL3 transcription and further contributes to m^6^A‐IGF2BP2‐dependent m^6^A modification of HIF‐1α, which mitochondrial contributes to metabolic reprogramming that enhances ferroptosis in alveolar epithelial cells.	[155]
Sepsis‐associated lung injury	METTL4	—	YTHDF2	Nrf2	↑	METTL4 promotes sepsis‐induced lung injury by inducing ferroptosis via m^6^A modification of Nrf2 to suppress its expression in YTHDF2‐dependant manner	[156]
Sepsis‐associated lung injury	METTL3	—	—	GPX4	↑	NETs damage AECs by triggering ferroptosis through the activation of the TLR9/MyD88/NF‐κB pathway, increasing METTL3 levels. This in turn leads to m^6^A modifications that reduce GPX4.	[157]
Sepsis‐associated lung injury	—	FTO	—	ACSL4	↓	FTO function as an ferroptosis inhibitor to attenuate inflammatory response by decreasing the stability of ACSL4 mRNA via YTHDF1 during sepsis‐associated lung injury.	[158]
Sepsis‐associated lung injury	—	—	YTHDC1	Angptl4	↑	Mir22hg drives ferritinophagy‐induced ferroptosis by recruiting the m^6^A reader YTHDC1, thereby boosting the stability of Angptl4 mRNA in sepsis‐related lung injury.	[159]
Sepsis‐associated lung injury	METTL14	—	IGF2BP2	STEAP1	↑	METTL14 contributes to septic lung injury by triggering ferroptosis via stabilising STEAP1 mRNA in IGF2BP2‐mediated m^6^A modification.	[160]
Asthma	—	ALKBH5	—	GPX4	↑	Silencing ALKBH5 inhibits asthma by suppressing ferroptosis via enhancing enrichment of m^6^A modification and expression of GPX4.	[161]
Asthma	METTL3	—	—	GPX4	↓	METTL3 suppresses asthma by inhibiting ferroptosis through stabilising GPX4 RNA via m^6^A modification.	[162]
COPD	—	—	YTHDF1	IREB2	↑	YTHDF1‐mediated recruitment of m^6^A‐modified circSAV1 results in the upregulation of IREB2, which induces ferroptosis in lung epithelial cells. In COPD, ferroptosis of airway epithelial cells drives airway remodelling, while ferroptosis of alveolar epithelial cells contributes to the development of emphysema. These findings highlight the pivotal role of m^6^A‐modified RNA interactions in regulating ferroptosis and their implications in respiratory diseases.	[163]
COPD	METTL3	—	YTHDF2	FSP1	↑	METTL3‐induced FSP1 mRNA methylation lead to decrease FSP1 in a YTHDF2‐dependent in COPD. FSP1 overexpression inhibits ferroptosis and alleviates emphysema.	[164]

Abbreviations: DHX58, DExH‐box helicase 58; I/R, liver ischemia/reperfusion (I/R) injury; PTGS2, prostaglandin‐endoperoxide synthase 2; TFRC, transferrin receptor protein 1.

#### 4.4.1 Sepsis‐Associated Lung Injury (SALI)

In SALI, m^6^A promotes ferroptosis through multiple converging mechanisms involving lipid metabolism, antioxidant suppression, and inflammatory signalling. METTL3 is upregulated by lactate signalling through GPR81 and p300‐mediated histone lactylation, leading to increased m^6^A modification and stabilisation of ACSL4 mRNA, which enhances lipid peroxidation and ferroptosis [154]. In parallel, METTL4 suppresses Nrf2 expression via m^6^A‐dependent mRNA decay, reducing SLC7A11 and GPX4 levels and weakening antioxidant defence [156].

FTO acts in an opposing manner by reducing ACSL4 mRNA stability and suppressing ferroptosis and inflammation in macrophages [158]. In addition, lncRNA Mir22hg promotes ferroptosis by stabilising Angptl4 mRNA through YTHDC1, enhancing ferritinophagy [159].

Inflammatory signalling further amplifies these effects. Neutrophil extracellular traps activate TLR9/MyD88/NF‐κB signalling and increase METTL3 expression, while also promoting m^6^A modification of HIF‐1α through IGF2BP2, leading to metabolic reprogramming and ferroptosis [155, 165]. METTL14 also contributes by stabilising STEAP1 mRNA, further enhancing ferroptotic injury [166].

Together, these findings indicate that ferroptosis in SALI is driven by coordinated activation of lipid metabolism, suppression of antioxidant defence, and inflammation‐associated m^6^A regulation, distinguishing it from other respiratory diseases where a single pathway predominates [154, 155, 156, 158, 159, 165, 166].

#### 4.4.2 Asthma

In asthma, m^6^A‐mediated ferroptosis is primarily regulated through GPX4‐dependent antioxidant pathways. ALKBH5 promotes ferroptosis by reducing GPX4 stability, whereas its silencing increases GPX4 expression and suppresses ferroptosis in bronchial epithelial cells [161]. In contrast, METTL3 stabilises m^6^A‐modified GPX4 mRNA and inhibits ferroptosis, improving cell viability [162].

These findings indicate that, unlike SALI, m^6^A regulation in asthma is mainly centred on maintenance of antioxidant capacity through GPX4 rather than lipid metabolism or inflammatory signalling [161, 162].

#### 4.4.3 Chronic Obstructive Pulmonary Disease (COPD)

In COPD, m^6^A‐mediated ferroptosis is primarily associated with iron metabolism and translational regulation. YTHDF1 enhances translation of IREB2 by recruiting m^6^A‐modified circSAV1, leading to increased iron metabolism activity and ferroptosis in lung epithelial cells [163].

This mechanism differs from both SALI and asthma, as m^6^A regulation in COPD is driven mainly by iron‐dependent ferroptosis rather than antioxidant suppression or lipid peroxidation pathways [163].

### 4.5 Regulating Ferroptosis by m^6^A in Kidney Diseases

Ferroptosis contributes to renal injury through dysregulated antioxidant defence and inflammatory signalling, and m^6^A modification regulates these processes in a context‐dependent manner [167, 168]. In sepsis‐associated acute kidney injury, ferroptosis is mainly driven by m^6^A‐dependent control of p53 signalling and inflammation rather than direct lipid metabolism (Figure 3 and Table 6).

**TABLE 6:** m^6^A modification of ferroptosis in kidney disease and other diseases.

Diseases	Writers	Erasers	Rreaders	Ferroptosis targets	Effect on ferroptosis	Biological functions	References
Septic acute kidney injury	METTL3	—	YTHDF1	SLC7A11; GPX4; FPN1	↑	Silencing METTL3 prevents sepsis‐associated acute kidney injury by inhibiting ferroptosis via the MDM2‐p53‐LMNB1 axis.	[167]
Septic acute kidney injury	WTAP	—	—	NF‐κB and JAK2/STAT3	↑	WTAP promotes sepsis‐associated acute kidney injury by inducing ferroptosis through upregulating LMNB1 to activate NF‐κB and JAK2/STAT3 pathways.	[168]
Osteoarthritis	—	—	IGF2BP1	MMP3	↑	IGF2BP1 promotes osteoarthritis by inducing ferroptosis in chondrocytes by stabilising MMP3 mRNA stability.	[169]
Osteoarthritis	METTL3	—	—	ACSL4	↓	METTL3‐mediated m^6^A modification downregulatin LPCAT3 improves osteoarthritis by inhibiting chondrocyte ferroptosis.	[170]
Osteoarthritis	METTL3	—	—	HMGB1	↑	METTL3 aggravates KOA pathogenesis by inducing ferroptosis IL‐1β‐triggered chondrocyte via upregulating HMGB1through m^6^A modification.	[171]
Osteoarthritis	METTL14	—	—	GPX4	↑	METTL14 enhances chondrocyte ferroptosis in osteoarthritis via m^6^A modification of GPX4.	[172]
Osteomyelitis	—	FTO	—	SLC7A11; GPX4	↓	FTO attenuates osteomyelitis by inhibiting ferroptosis through inhibiting the MDM2‐TLR4 axis in SA‐induced osteomyelitis.	[173]
T2DM	METTL3	—	YTHDF2	GSTK1	↑	METT14/YTHDF2 mediated m^6^A methylation of PGC‐1α facilitates ferroptosis via suppressing GSTK1 in arsenic‐induced hepatic insulin resistance.	[174]
Diabetic osteoporosis	METTL3	—	—	ASK1‐p38	↑	METTL3/ASK1‐p38 signalling pathway contributes to diabetic bone loss by inducing osteoblast ferroptosis.	[175]
Male reproductive diseases	—	FTO	YTHDF1; YTHDF2	TFRC; SLC7A11	↑	FTO contributes to bisphenol F‐induced disruption of the blood‐testis barrier by promoting ferroptosis through the YTHDF1/TFRC and YTHDF2/SLC7A11 signalling pathways.	[176]
Male testosterone deficiency	METTL3; METTL14	—	—	Plin4	↑	MELLT3‐METTL14 upregulates Plin4 in an m^6^A‐dependent manner to promotes testicular Leydig cells ferroptosis and induce decreased testosterone.	[177]
Preeclampsia	METTL3	—	—	ACSL4	↑	METTL3 promotes trophoblast ferroptosis by stabilising the ACSL4 m^6^A modification in preeclampsia.	[178]
IVDD	—	—	YTHDF1	SLC7A11	↓	HIF‐1α‐mediated upregulation of YTHDF1 reduces ferroptosis in nucleus pulposus cells (NPC) and delays intervertebral disc degeneration (IVDD) by enhancing SLC7A11 translation in a manner dependent on m^6^A modification.	[179]
Ankylosing spondylitis	—	ALKBH5	—	HMGB1	↓	Decreased ALKBH5 mediated upregulation of lncRNA DDIT3 promotes development of ankylosing spondylitis by decreasing chondrocyte survival through inducing ferroptosis, and ECM breakdown via the miR‐142‐3p.1/HMGB1 axis.	[180]

Abbreviations: DHX58, DExH‐box helicase 58; I/R, liver ischemia/reperfusion (I/R) injury; IVDD, intervertebral disc degeneration; PTGS2, prostaglandin‐endoperoxide synthase 2; T2DM, type 2 diabetes mellitus; TFRC, transferrin receptor protein 1.

#### 4.5.1 Sepsis‐Associated Acute Kidney Injury (SA‐AKI)

METTL3 promotes ferroptosis by stabilising MDM2 mRNA through m^6^A modification and YTHDF1 binding, leading to p53 suppression and activation of LMNB1 [167]. This reduces SLC7A11, GPX4, and FPN1 levels and enhances ferroptotic injury. Silencing METTL3, MDM2, or LMNB1 suppresses ferroptosis and alleviates renal damage [167].

WTAP further enhances ferroptosis by increasing m^6^A modification and expression of LMNB1 [168]. Elevated LMNB1 activates NF‐κB and JAK2/STAT3 signalling, amplifying inflammatory injury. WTAP inhibition reduces ferroptosis, whereas LMNB1 overexpression reverses this effect [168].

These findings indicate that m^6^A‐mediated ferroptosis in SA‐AKI is primarily driven by MDM2–p53 signalling and LMNB1‐dependent inflammation. This mechanism differs from other organs, where ferroptosis is more directly linked to lipid peroxidation or antioxidant pathways [167, 168].

### 4.6 Regulating Ferroptosis by m^6^A in Other Diseases (Figure 3 and Table 6)

*Degenerative musculoskeletal diseases*, including osteoarthritis, ankylosing spondylitis, and intervertebral disc degeneration, exhibit distinct m^6^A‐dependent ferroptosis patterns despite sharing structural tissue damage. In osteoarthritis, METTL3, METTL14, and IGF2BP1 enhance ferroptosis by stabilising pro‐ferroptotic transcripts such as HMGB1 and MMP3 or suppressing GPX4 expression, thereby promoting cartilage degradation [171, 172, 181]. Similarly, in ankylosing spondylitis, reduced ALKBH5 increases the stability of lncRNA DDIT3, which elevates HMGB1 expression and accelerates ferroptosis‐associated extracellular matrix damage [180]. In contrast, intervertebral disc degeneration shows an opposing regulatory direction, where HIF‐1α‐induced YTHDF1 enhances SLC7A11 translation and GPX4 expression, thereby suppressing ferroptosis and preserving nucleus pulposus cell viability [179]. These findings indicate that even within related musculoskeletal disorders, m^6^A regulation can either promote or inhibit ferroptosis depending on the dominant target transcripts.

*Metabolic disorders*, including type 2 diabetes mellitus and diabetic osteoporosis, display ferroptosis regulation linked to metabolic reprogramming. In type 2 diabetes, METTL14 promotes degradation of PGC‐1α via YTHDF2, impairing mitochondrial metabolism and enhancing oxidative stress–induced ferroptosis [182]. In diabetic osteoporosis, METTL3 stabilises ASK1 mRNA and activates p38 signalling, driving ferroptosis in osteoblasts [175]. Although both conditions involve metabolic stress, the upstream regulators and downstream pathways differ, with mitochondrial dysfunction dominating in diabetes and stress kinase signalling in bone tissue.

*Reproductive and developmental disorders* exhibit ferroptosis regulation closely associated with iron handling and lipid metabolism. In male reproductive toxicity, reduced FTO expression increases m^6^A modification of TFRC and SLC7A11 transcripts, disrupting iron homeostasis and promoting ferroptosis [176]. In cadmium‐induced testicular injury, METTL3 and METTL14 enhance Plin4 expression, leading to lipid droplet accumulation and ferroptosis in Leydig cells [177]. In preeclampsia, METTL3 stabilises ACSL4 mRNA, promoting ferroptosis in trophoblasts under hypoxic conditions [183]. These findings suggest that ferroptosis in reproductive systems is driven by distinct but converging mechanisms involving iron metabolism, lipid remodelling, and hypoxia‐responsive pathways.

*Inflammatory and infectious bone‐related conditions*, such as osteomyelitis, are characterised by m^6^A‐dependent disruption of antioxidant defenses. In osteomyelitis, decreased FTO expression increases m^6^A modification of MDM2 and TLR4 transcripts, enhancing inflammatory signalling and promoting degradation of SLC7A11 and GPX4, thereby inducing ferroptosis in bone marrow stromal cells [173]. This mechanism differs from other disease categories by linking ferroptosis more directly to inflammation‐driven signalling cascades rather than metabolic or transcriptional regulation alone.

Across these disease categories, m^6^A‐dependent ferroptosis does not follow a uniform regulatory pattern. Instead, disease‐specific differences arise from selective targeting of pro‐ or anti‐ferroptotic genes, variation in dominant m^6^A regulators, and distinct cellular metabolic states. This context‐dependent heterogeneity highlights that m^6^A‐mediated ferroptosis should be interpreted within individual disease frameworks rather than generalised across conditions.

### 4.7 Regulating Ferroptosis by m^6^A in Cancers

Emerging evidence indicates that m^6^A modification critically regulates ferroptosis in cancer through post‐transcriptional control of key metabolic and antioxidant pathways. Rather than following a uniform mechanism, m^6^A‐dependent ferroptosis exhibits marked tumour‐type heterogeneity, with distinct regulators targeting different transcripts to either suppress or promote ferroptotic cell death (Figure 3 and Table 7).

**TABLE 7:** Epigenetic modification of ferroptosis by m^6^A in cancer.

Diseases	Writer	Eraser	Reader	Targets	Effect on ferroptosis	Biological functions	References
NSCLC	METLL3	—	—	FSP1	↓	Exosomal miR‐4443 enhances resistance to cisplatin by inhibiting ferroptosis through promoting METLL3‐mediated m^6^A modification of FSP1.	[184]
NSCLC	METTL3	—	IGF2BP3	GPX4; SLC3A2; FTH1; ACSL3	↓	Increased IGF2BP3 promotes tumorigenesis by stabilising m^6^A‐methylated mRNAs encoding anti‐ferroptosis factors, mediated by METTL3, thus inhibiting ferroptosis.	[185]
NSCLC	METTL3	—	YTHDF1	SLC7A11	↓	METTL3 enhances tumour growth by stabilising SLC7A11 m^6^A modifications, suppressing ferroptosis.	[186]
NSCLC	METTL3	—	—	PROM2	↓	ATF1 promotes METTL3 transcription, thereby stabilising PROM2 mRNA and increasing ferroptosis resistance.	[187]
NSCLC	METTL3	—	—	CREB	↓	METTL3‐mediated upregulation of ZDHHC16 expression inhibits ferroptosis by inhibiting CREB ubiquitination in NSCLC.	[188]
NSCLC	METTL14	—	—	GPX4	↓	METTL14 promotes tumour malignancy by inhibiting ferroptosis through m^6^A modification‐mediated upregulation of GPX4 in IGF2BP1 dependant manner in NSCLC.	[189]
NSCLC	KIAA1429	—	—	p53	↓	KIAA1429 promotes NSCLC by inhibiting ferroptosis through inactivating p53 in NSCLC, highlighting targeting KIAA1429 as an effective strategy to kill NSCLC.	[190]
NSCLC	—	ALKBH5	—	SLC7A11	↑	ALKBH5 upregulation inhibits NSCLC progression by inducing ferroptosis through downregulating SLC7A11 transcription by decreasing m^6^A modification.	[191]
NSCLC	—	—	IGF2BP3	SLC7A11/GPX4	↓	IGF2BP3 promotes tumorigenesis by suppressing ferroptosis via m^6^A‐dependent upregulation of TFAP2A to promote the transcription of SLC7A11 and GPX4.	[192]
NSCLC	—	—	YTHDC1	FSP1	↑	YTHDC1 functions as a tumour progression suppressor by inducing ferroptosis through decreasing FSP1 mRNA stability.	[193]
NSCLC	—	—	YTHDC1	HuR	↑	YTHDC1‐mediated LINC00641 downregulation increases HuR protein level promotes ferroptosis sensitivity.	[194]
NSCLC	—	—	YTHDF1	—	↓	YTHDF1 promotes cancer by enhancing ferritin translation in an m^6^A‐dependent manner, preventing ferroptosis.	[195]
NSCLC	—	—	YTHDC2	SLC7A11	↑	YTHDC2 suppresses LUAD tumorigenesis by inhibiting SLC7A11 mRNA and promoting its decay in an m^6^A‐dependent manner.	[196]
NSCLC	—	—	YTHDC2	HOXA13	↑	YTHDC2 inhibits LUAD progression by destabilising SLC7A11 and HOXA13 mRNAs via m^6^A methylation, inducing ferroptosis.	[197]
NSCLC	—	FTO	—	Lipocalin 2		FTO reduces miR‐138‐5p maturation, downregulating lipocalin 2 (LCN2), and inhibiting ferroptosis, contributing to gefitinib resistance.	[198]
HCC	METTL3	—	IGF2BP1	SLC7A11	↓	METTL3‐mediated m^6^A modification stabilises SLC7A11 mRNA via IGF2BP1, enhancing ferroptosis resistance in hepatoblastoma (HB).	[199]
HCC	METTL16	—	IGF2BP2	Lactotransferrin	↓	METTL16 suppresses ferroptosis in HCC by stabilising SENP3 mRNA, upregulating LTF through de‐SUMOylation.	[200]
HCC	WTAP	—	YTHDC2	ATG5	↑	YTHDC2 binds WTAP‐modified ATG5 mRNA, increasing ferritinophagy and ferroptosis.	[201]
HCC	—	—	IGF2BP3	RRM2	↓	IGF2BP3 promotes HCC by repressing ferroptosis via m^6^A‐dependent regulation of RRM2 mRNA.	[202]
HCC	KIAA1429	—	—	SLC7A11	↓	KIAA1429 stabilises SLC7A11.	[203]
HCC	WTAP	—	IGF2BP1	PARK7	↓	WTAP‐mediated m^6^A modification of CircCMTM3 promotes carcinogenesis by suppressing ferroptosis through recruiting IGF2BP1 to stabilise PARK7.	[204]
HCC	WTAP	—		NOA1	↓	WTAP affect the m^6^A methylation of NOA1 and activates the GPX4‐axis to inhibit ferroptosis.	[205]
HCC	—	—	IGF2BP3	SLC7A11	↓	LINC00942 recruits IGF2BP3 to stabilise SLC7A11, suppressing ferroptosis and inducing Treg‐mediated immunosuppression.	[206]
HCC	—	—	YTHDF2	FSP1	↓	HDLBP stabilises lncFAL, preventing Trim69‐mediated FSP1 polyubiquitination degradation and ferroptosis. The splicing of lncFAL was increased by YTHDF2 in a m^6^A‐dependent manner.	[207]
HCC	—	—	IGF2BP3	Nrf2	↓	IGF2BP3 stabilises Nrf2 mRNA, resisting sorafenib‐induced ferroptosis.	[208]
HCC	METTL3; WTAP	—	YTHDF2	ACSL5	↓	METTL3 and WTAP‐mediated degradation of PPARGC1A in an m^6^A‐YTHDF2‐dependent way under normoxia and hypoxia, respectively.	[209]
HCC	METTL3	—	YTHDF3	DECR1	↑	METTL3 interacts with Lnc HNF4A‐AS1, resulting in m^6^A modification of DECR1 mRNA, which decreases DECR1 expression via YTHDF3‐dependent mRNA degradation. Decreased HNF4A‐AS1 upregulates DECR1 overexpression and decreases intracellular PUFA content, thereby promoting resistance to sorafenib‐induced ferroptosis.	[210]
Cholangiocarcinoma	METTL16	—	—	ATF4	↓	METTL16 inhibits ferroptosis by enhancing the m^6^A level and expression of ATF4 mRNA by inhibiting its decay.	[211]
Hepatoblastoma	METTL3	—	YTHDF2	ATF4/PSAT1	↓	m^6^A modification‐mediated degradation of LATS2 promotes ferroptosis resistance via the YAP1/ATF4/PSAT1 axis.	[212]
CRC	METTL3	—	—	ACSL3	↓	CAFs enhance cancer proliferation and metastasis by suppressing ferroptosis through exosomal METTL3‐induced ACSL3 m^6^A modification.	[213]
CRC	METTL3	—	IGF2BP2	FOXM1	↓	METTL3‐mediated upregulation of lncRNA ABHD11‐AS1 exacerbates tumour progression by blocking ferroptosis via IGF2BP2‐TRIM21 interactions, leading to FOXM1 overexpression.	[214]
CRC	—	FTO		SLC7A11/GPX4	↓	Elevated FTO promotes ferroptosis resistance by increasing SLC7A11 and GPX4 expressions in a YTHDF2‐dependent manner.	[215]
CRC	—	—	ALKBH5	SLC7A11	↑	ALKBH5 facilitates ferroptosis in CRC by reducing SLC7A11 expression through m^6^A demethylation.	[216]
GC	—	—	YTHDF2	CBS	↓	HIF‐1α induces lncRNA‐CBSLR, which recruits YTHDF2 and CBS mRNA, reducing CBS stability and ACSL4 methylation, leading to ACSL4 proteasomal degradation and ferroptosis suppression.	[217]
GC	METTL3	—	YTHDC1	SLC7A11	↓	METTL3/YTHDC1‐mediated FAM120A upregulation drives cisplatin resistance by enhancing SLC7A11 expression.	[218]
GC	METTL3	—	—	miR‐27a‐5p/BTF3	↓	METTL3‐induced m^6^A modification of lncRNA FAM230B promotes gastric cancer progression. It does this by controlling the miR‐27a‐5p/BTF3 pathway, inhibiting ferroptosis.	[219]
GC	METTL5	—	IGF2BP1	Nrf2	↓	METTL5 stabilises Nrf2 mRNA via IGF2BP1, reducing ferroptosis and PBMC‐mediated antitumor immunity.	[220]
Thyroid cancer	—	ALKBH5	—	TIAM1/Nrf2	↑	ALKBH5 promotes ferroptosis in thyroid cancer by reducing Nrf2 activation via TIAM1 m^6^A demethylation	
Thyroid cancer	—	FTO	—	SLC7A11	↑	FTO inhibits thyroid cancer progression by inducing ferroptosis through downregulating SLC7A11 by m^6^A methylation.	[221]
Thyroid cancer	METTL3	—	—	Hsa_Circ_0136959	↑	METTL3‐Induced m^6^A modification promotes Hsa_Circ_0136959 expression to suppress cancer via inducing ferroptosis.	[222]
BC	METTL16	—	—	GPX4	↓	METTL16 promotes breast cancer progression by surpressing ferroptosis through enhancing GPX4 expression via m^6^A modification.	[223]
BC	METTL14		YTHDC2	FGFR4	↓	Downregulated METTL14 hinder YTHDC2‐mediated FGFR4 mRNA degradation, causing FGFR4 accumulation in anti‐HER2‐resistant breast cancer cells. FGFR4 phosphorylates GSK‐3β, activating β‐catenin/TCF4 signalling to upregulate FPN1 and SLC7A11 transcription. Elevated SLC7A11 and FPN1 expression mitigates ferroptosis, contributing to anti‐HER2 resistance.	[224]
BC	METTL3	—	—	SLC7A11	↓	METTL3‐modified lncRNA DSCAM‐AS1 promotes tumour progression in breast cancer through inhibiting ferroptosis.	[225]
BC	WTAP	—	—	LCN2	↓	WTAP drives TNBC progression by inhibiting ferroptosis via upregulating LCN2 through m^6^A modification of NUPR1.	[226]
BC	—	—	IGF2BP1	GPX4	↓	RUNX1‐IT1 enhances cell proliferation, invasion, and tumour growth by suppressing ferroptosis through GPX4 upregulation in an IGF2BP1‐dependent manner.	[227]
BC	—	—	IGF2BP2	VDAC2	↑	LncRNA SH3BP5‐AS1 promotes ferroptosis by downregulating VDAC2 expression via IGF2BP2‐dependent mechanisms.	[228]
BC	METTL3	—	FMRP	SLC7A11	↓	FMRP inhibits ferroptosis by catalysing m^6^A modification of SLC7A11 mRNA, enhancing SLC7A11 translation in a METTL3‐dependent pathway.	[229]
Cervical cancer	METTL14	—	—	FTH1	↑	METTL14 induces sorafenib‐triggered ferroptosis by reducing FTH1 mRNA stability through m^6^A methylation.	[230]
Cervical cancer	METTL3	—	—	KRAS	↓	miR‐30c‐5p suppresses proliferation and metastasis by inducing ferroptosis via targeting the METTL3/KRAS axis in cervical cancer.	[231]
Cervical cancer	METTL14	—	IGF2BP1	—	↓	IGF2BP1 in m^6^A‐dependent manner mediae translation of N‐acetyltransferase 10 (NAT10) mRNA, which drives tumorigenesis by inducing N4‐acetylcytidine (ac4C) modification of ACOT7 mRNA to augment its stability and translation.	[232]
Endometrial cancer	—	—	HNRNPA2B1	LCN2	↓	HNRNPA2B1‐mediated m^6^A modification of FOXM1 promotes drug resistance by suppressing ferroptosis via LCN2 upregulation in EC cells.	[233]
Endometrial cancer	METTL14	—	YTHDF2	GPX4	↑	METTL14 overexpression, induced by PRMT3 inhibition, increases ferroptosis via YTHDF2‐mediated GPX4 mRNA destabilisation.	[234]
OC	—	—	IGF2BP1	FTH1	↓	LncRNA CACNA1G‐AS1 promotes ovarian cancer proliferation by inhibiting ferroptosis through IGF2BP1‐driven FTH1 expression.	[235]
PCa	—	—	YTHDF1	—	↓	YTHDF1 inhibits antitumor immunity and ferroptosis in prostate cancer through the m^6^A‐PD‐L1 axis.	[236]
GBM	METTL3	—	—	SLC7A11	↓	NKAP prevents ferroptosis by recruiting SFPQ for SLC7A11 mRNA splicing in an METTL3/m^6^A‐dependent manner.	[237]
GBM	—	ALKBH5	YTHDF2	GCLM	↓	Epidermal growth factor receptor (EGFR) promotes glioblastoma progression by retaining ALKBH5 in the nucleus, reducing ferroptosis via glutathione production.	[238]
GBM	METTL3	—	—	GPX4	↓	C5aR1 drives glioma progression by inhibiting ferroptosis through METTL3‐mediated GPX4 upregulation via ERK1/2.	[239]
Glioma	—	—	IGF2BP3	GPX4	↓	The loss of IGF2BP3 triggers ferroptosis in glioma through regulation of GPX4 expression.	[240]
Glioma	METTL3	—	—	Nrf2	↑	Silencing lncRNA SNAI3‐AS1 in glioma enhances ferroptosis by blocking SND1‐mediated Nrf2 m^6^A recognition.	[241]
NPC	—	FTO	—	OTUB1	↓	FTO promotes radioresistance by inhibiting ferroptosis via OTUB1 expression.	[242]
NPC	—	FTO	YTHDC1	SLC7A11	↓	LncRNA HOTAIRM1 promotes CD44 alternative splicing via FTO‐mediated demethylation, thereby NPC enhancing radioresistance by inhibiting irradiation‐induced ferroptosis.	[243]
HPSCC	—	ALKBH5	IGF2BP2	Nrf2	↓	ALKBH5 suppresses ferroptosis by activating Nrf2 in an m^6^A‐IGF2BP2‐dependent manner.	[244]
OSCC	—	FTO	—	ACSL3 and GPX4	↑	FTO promotes ferroptosis by reducing ACSL3 and GPX4 via m^6^A demethylation.	[245]
OSCC	KIAA1429	—	YTHDF1	PGK1	↓	KIAA1429 promotes the OSCC aerobic glycolysis and inhibits the ferroptosis of OSCC through YTHDF1‐mediated PGK1 mRNA stability.	[246]
LC	RBM15	—	IGF2BP3	GPX4; ACSL4	↓	RBM15 promotes DDP resistance by inhibiting ferroptosis through stabilising KDM5B in an IGF2BP3‐dependent manner, thereby downregulating lncRNA FER1L4 and upregulating lncRNA KCNQ1OT1, both of which bind to MYC to decreasing GPX4 and increasing ACSL4, respectively.	[247]
LC	ZC3H13	—	—	DUOX1	↓	ZC3H13 reduces DUOX1‐mediated ferroptosis in LSCC cells through m^6^A‐dependent modification.	[248]
GBC	—	—	IGF2BP2	SCD1	↓	IGF2BP2‐mediqated stability of circEZH2 promotes the proliferation by inhibiting ferroptosis through promoting SCD1 expression via sponging miR‐556‐5p in GBC cells.	[249]
Bladder cancer	—	—	YTHDF3	SLC7A11	↑	Decreased YTHDF3 prolongs SLC7A11 RNA stability, inhibiting ferroptosis in cisplatin‐resistant bladder cancer.	[250]
Bladder cancer	WTAP	—	YTHDF1	Nrf2	↓	WTAP promotes bladder cancer progression by suppressing ferroptosis via YTHDF1‐mediated Nrf2 upregulation.	[251]
ESCC	METTL14	—	—	ACSL4	↑	METTL14 enhances ACSL4 m^6^A modification, sensitising ESCC to irradiation by increasing ferroptosis.	[252]

Abbreviations: ACSL3, acyl‐CoA synthetase long chain family member 3; ALKBH5, AlkB homologue 5; BC, breast cancer; C5aR1, complement C5a receptor 1; CBS, cystathionine‐beta‐synthase; ccRCC, clear cell renal cell carcinoma; CRC, colorectal cancer; ESCC, oesophageal squamous cell carcinoma; FSP1, ferroptosis suppressor protein 1; FTH1, ferritin heavy chain 1; FTO, fat mass and obesity‐associated protein; GBC, gallbladder cancer; GBM, glioblastoma; GC, gastric cancer; GCLM, glutamate‐cysteine ligase modifier subunit; GPX4, glutathione peroxidase 4; HCC, hepatocellular carcinoma; HDLBP, high‐density lipoprotein‐binding protein; HPSCC, hypopharyngeal squamous cell carcinoma; LC, laryngeal carcinoma; NKAP, NF‐κB activating protein; NOA1, nitric oxide‐associated protein 1; NPC, nasopharyngeal carcinoma; NSCLC, non‐small cell lung carcinoma; OC, ovarian cancer; RCC, renal cell carcinoma; SFPQ, splicing factor proline and glutamine‐rich; SLC3A2, solute carrier family 3 member 2.

One major regulatory mode involves antioxidant defence systems, particularly SLC7A11, GPX4, and Nrf2. In multiple cancers including non‐small cell lung cancer (NSCLC), hepatocellular carcinoma (HCC), colorectal cancer (CRC), and gastric cancer (GC), m^6^A writers such as METTL3 and METTL14 stabilise SLC7A11 or GPX4 transcripts via IGF2BP family proteins, thereby suppressing ferroptosis and promoting tumour progression [2, 187, 200, 215, 218, 253]. Similarly, reader proteins including IGF2BP3 and YTHDF1 enhance antioxidant capacity by stabilising Nrf2 or ferritin‐related transcripts, further contributing to ferroptosis resistance [196, 200, 254]. In contrast, certain regulators exhibit tumour‐suppressive effects by destabilising these same pathways. For example, ALKBH5 reduces SLC7A11 expression and promotes ferroptosis in NSCLC and colorectal cancer [216, 255], while YTHDC2 destabilises SLC7A11 and induces ferroptosis in lung adenocarcinoma [196]. These findings demonstrate that identical ferroptosis‐related targets can be differentially regulated depending on the dominant m^6^A machinery in each tumour.

Iron metabolism and lipid peroxidation pathways represent another layer of divergence. In NSCLC and HCC, m^6^A‐dependent stabilisation of ferroptosis suppressors such as FSP1 and PROM2 reduces lipid peroxidation and enhances tumour survival [184, 187]. Conversely, in hepatocellular carcinoma, YTHDC2 promotes ferroptosis by enhancing ATG5 translation and triggering ferritinophagy, increasing intracellular iron levels [201]. In colorectal cancer, METTL3‐enriched exosomes derived from cancer‐associated fibroblasts stabilise ACSL3 and inhibit ferroptosis, highlighting a stromal‐dependent mechanism [213]. These observations indicate that m^6^A regulation of ferroptosis can either limit or enhance lipid peroxidation depending on whether iron release or lipid detoxification pathways are preferentially targeted.

Non‐coding RNA–mediated regulation further contributes to tumour‐specific ferroptosis control. In hepatocellular carcinoma and gastric cancer, lncRNAs such as LINC00942 and FAM230B are stabilised through m^6^A modification, leading to increased SLC7A11 expression or suppression of pro‐ferroptotic signalling, thereby enhancing ferroptosis resistance [206, 213, 219]. In colorectal cancer, METTL3‐dependent stabilisation of lncRNA ABHD11‐AS1 promotes FOXM1 expression and suppresses ferroptosis [256]. In contrast, certain non‐coding RNAs enhance ferroptosis, as observed in breast cancer where SH3BP5‐AS1 reduces VDAC2 expression and promotes ferroptotic cell death [228]. These findings highlight that m^6^A‐modified non‐coding RNAs form diverse regulatory networks that differ substantially across tumour types.

m^6^A‐dependent ferroptosis also plays a critical role in therapy response and tumour microenvironment interactions. In lung adenocarcinoma, FTO‐mediated suppression of ferroptosis contributes to gefitinib resistance, while its inhibition restores drug sensitivity [198]. In breast cancer, altered m^6^A regulation of FGFR4 and GPX4 underlies resistance to anti‐HER2 therapy [224]. In cervical and oesophageal cancers, increased ferroptosis via METTL14 enhances sensitivity to sorafenib or radiotherapy [230, 252]. In prostate cancer, YTHDF1 promotes immune evasion and ferroptosis resistance through PD‐L1 regulation, linking m^6^A‐dependent ferroptosis to antitumor immunity [236]. These findings indicate that ferroptosis regulation is tightly coupled to therapeutic outcomes, with distinct m^6^A pathways shaping treatment sensitivity in different cancers.

Overall, m^6^A‐mediated ferroptosis in cancer is highly context‐dependent. While common regulators such as METTL3, METTL14, FTO, and IGF2BP proteins are repeatedly involved, their functional outcomes vary depending on target selection, cellular metabolism, and tumour microenvironment. This heterogeneity underscores that ferroptosis is not governed by a universal pathway across cancers, but instead reflects tumour‐specific regulatory programs that may be selectively targeted for therapeutic intervention.

## 5 Modulation of m^6^A Regulating Ferroptosis for Disease Therapy

**FIGURE 4:**
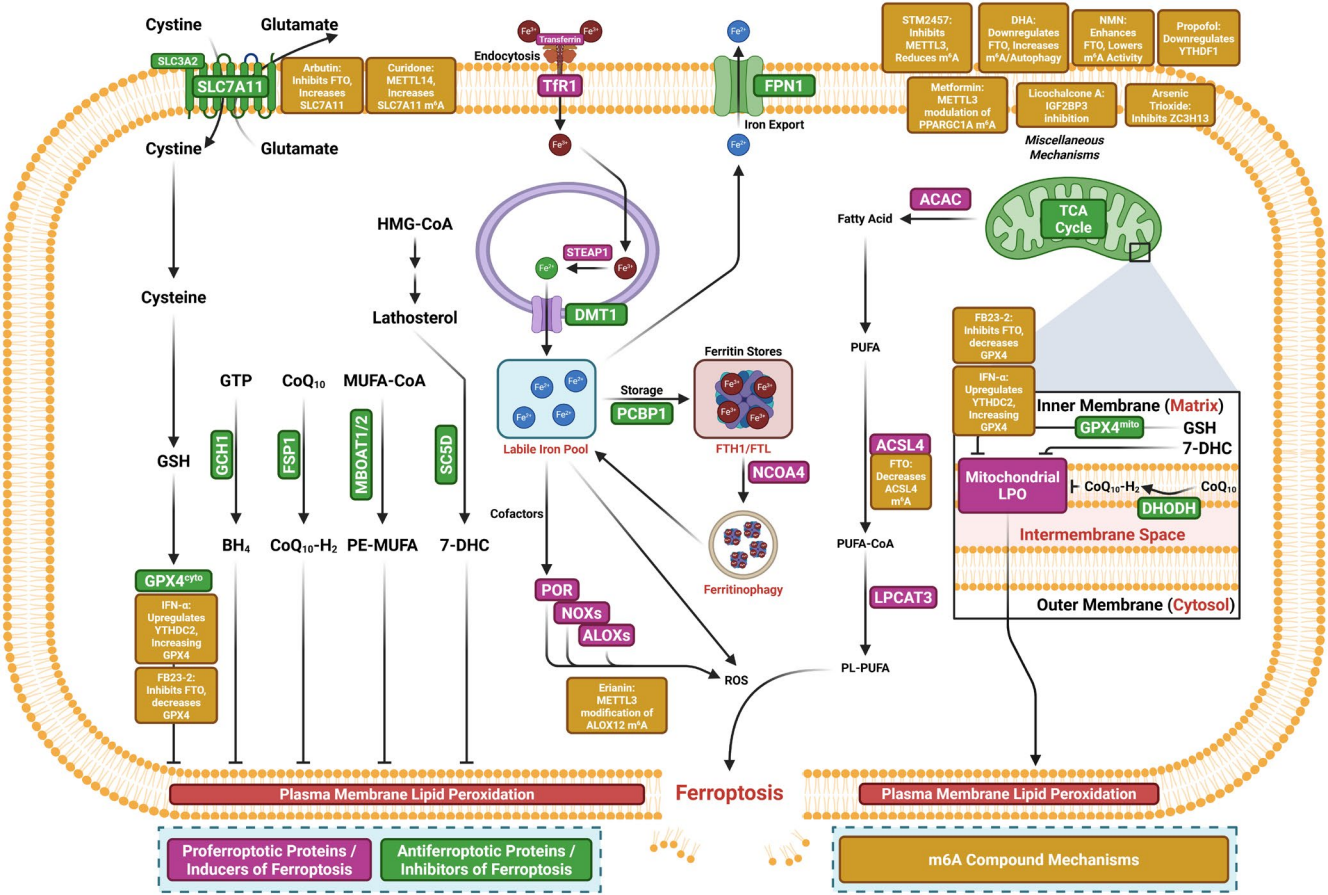
Mechanisms of compounds targeting m^6^A regulating ferroptosis in diseases. Antiferroptotic drugs prevent end‐stage plasma membrane peroxidation through several distinct mechanisms: Activation of the Nrf2 pathway, inhibition of lipid peroxidation, direct inhibition of ferroptosis, modulation of the antioxidative GPX4/SLC7A11 pathways, and other related mechanisms.

Therapeutic strategies targeting ferroptosis through modulation of m^6^A modification are being increasingly explored across multiple diseases. Pharmacological and biologically derived interventions that regulate m^6^A‐dependent ferroptosis have shown therapeutic potential in diverse pathological settings, as illustrated in Figure 4 and Table 8.

**TABLE 8:** Compounds targeting m^6^A regulating ferroptosis in diseases.

Diseases	Compounds	type	m^6^A targets	Ferroptosis targets	Biological functions	References
Ischemic stroke	Astragaloside IV	Saponin	FTO	ATF3; ACSL4	Astragaloside IV suppresses neuronal injury by inhibiting ferroptosis through promoting FTO transcriptionally via upregulating ATF3, decreasing ACSL4 m^6^A levels in ischemic stroke.	[257]
Ischemic stroke	Propofol	Anaesthetic	YTHDF1	BECN1	Propofol attenuates OGD/R‐induced ferroptosis in HT22 cells by down‐regulating HIF‐1α, thereby decreasing YTHDF1 and BECN1.	[112]
NAFLD	Arbutin	Natural antioxidant	FTO	SLC7A11	Arbutin attenuates NAFLD by inhibiting ferroptosis by increasing expression of SLC7A11 via through inhibiting FTO. This results in higher m^6^A methylation of SLC7A11, boosting its expression and ultimately preventing ferroptosis.	[258]
HIRI	Nicotinamide mononucleotide	—	FTO	ACSL4; TFRC	NMN ameliorate older HIRI by suppressing ferroptosis through increasing FTO demethylase activity.	[145]
HIRI	Interferon‐α	—	YTHDC2	GPX4	IFN‐α inhibits liver damage and hepatic ferroptosis by upregulates DHX58, leading to the recruitment of YTHDC2 to promote the translation of m^6^A‐modifed GPX4 mRNA.	[144]
HIRI	Dihydroartemisinin	Artemisinin derivative	FTO/YTHDF1	BECN1	DHA alleviates liver fibrosis by activating HSC ferroptosis through downregulating FTO and increasing m^6^A modifications in BECN1 mRNA in an YTHDF1‐mediated m^6^A‐dependent manner thus triggering autophagy activation.	[259]
Sepsis‐associated lung injury	STM2457	METTL3 inhibitor	METTL3	ACSL4	METTL3 can reduce ferroptosis and lung injury caused by sepsis.	[154]
Osteoarthritis	BMSC‐Exos	—	METTL3	ACSL4	BMSC‐Exos inhibits the IL‐1β‐induced chondrocyte ferroptosis by downregulating m^6^A modification of ACSL4 mRNA and ACSL4 stability via down‐regulating METTL3.	[260]
Endometrial injury	MB‐exos	—	METTL3/YTHDF2	HMOX1	MB‐exos attenuates injured endometrium by inhibiting ferroptosis through decreasing METTL3 mediated m^6^A modifcations‐induced degradationin of HMOX1 mRNA in YTHDF2‐dependant manner.	[261]
NSCLC	Arsenic trioxide	ZC3H13 inhibitor	ZC3H13	GPX4	ATO impairs the stemness of LUAD stem cells by enhancing ferroptosis through reducing ZC3H13 to suppress m^6^A medication.	[262]
NSCLC	CREB‐IN‐1 TFA	CREB inhibitor			CREB inhibitor reduces silencing ZDHHC16‐induced induction of ferroptosis, thereby decreasing cell proliferation and metastasis, promoted ferroptosis and mitochondrial damage.	[188]
CRC	Mupirocin	FTO inhibitor	FTO	SLC7A11; GPX4	A novel inhibitor of FTO mupirocin inhibits tumour growth through inducing induces ferroptosis in CRC.	[215]
CRC	Curdione	Sesquiterpenoids	METTL14	SLC7A11; SLC3A2	Curdione induces ferroptosis by boosting METTL14 expression, which enhances m^6^A modification of SLC7A11 and SLC3A2 mRNAs. Through a m^6^A‐dependent manner, the m^6^A reader YTHDF2 then accelerates the degradation of these mRNAs, decreasing their stability and promoting ferroptosis in CRC.	[263]
CRC	MK2206	AKT inhibitor	FTO	GPX4	MK2206 induces ferroptosis by promoting degradation of GPX4 mRNA to decrease expression level of GPX4 through downregulating FTO in YTHDF2 dependant manner.	[264]
RCC	Erianin	Bibenzyl compound	METTL3/FTO	ALOX12; SLC7A11	Erianin induces ferroptosis by upregulating METTL3 and downregulates FTO to promotes m^6^A modification of ALOX12 and p53, thereby decreasing SLC7A11 in HuRCSCs.	[265]
GBM	ALKBH5i1; ALKBH5i2	ALKBH5 inhibitors	ALKBH5	—	ALKBH5 inhibitors boosts the anti‐tumour effificacy of EGFR or glutathione inhibitors.	[238]
Endometrial Carcinoma	SGC707	PRMT3 inhibitor	—	—	The specific PRMT3 inhibitor SGC707 boosts anti‐PD‐1 therapy through inducing ferroptosis in a patient‐derived xenograft model. Inhibiting PRMT3 in particular enhances tumour suppression when treating with cisplatin and radiation therapy.	[234]
HCC	Metformin	AMPK agonist	METTL3	—	Metformin increases PPARGC1A expression by decreasing its m^6^A modification through the inhibition of METTL3, which could be beneficial for HCC patients with PPARGC1A dysregulation.	[209]
AML	Licochalcone A	Naturally flavonoid	IGF2BP3	—	Lico A promotes ferroptosis by suppressing the IGF2BP3/MDM2 cascade in AML.	[266]

Abbreviations: AML, acute myeloid leukaemia; CRC, colorectal cancer; GBM, glioblastoma; HCC, hepatocellular carcinoma; HIRI, hepatic ischaemia/reperfusion injury; IFN‐α, interferon‐α; NAFLD, non‐alcoholic fatty liver disease; NSCLC, non‐small cell lung cancer; RCC, renal cell carcinoma.

These therapeutic effects are not mediated through a single shared mechanism. Instead, different interventions act on distinct m^6^A regulators and ferroptosis‐related targets depending on disease context, including antioxidant defense, iron metabolism, lipid peroxidation, and autophagy‐associated pathways.

### 5.1 Ischemic Stroke

Astragaloside IV reduces neuronal injury by inhibiting ferroptosis through upregulation of ATF3, which promotes FTO transcription and decreases ACSL4 m^6^A levels during ischemic stroke [257]. Propofol alleviates OGD/R‐induced ferroptosis in HT22 cells by suppressing HIF‐1α, leading to decreased YTHDF1 and BECN1 expression. This effect is reversed by YTHDF1 overexpression, indicating that propofol suppresses ferroptosis by interfering with hypoxia‐responsive m^6^A regulation [112].

These findings suggest that therapeutic regulation in ischemic stroke primarily targets lipid peroxidation and autophagy‐related ferroptotic pathways through upstream modulation of FTO‐ and YTHDF1‐dependent signalling.

### 5.2 Non‐Alcoholic Fatty Liver Disease (NAFLD)

Arbutin, a natural antioxidant derived from *Arctostaphylos uva‐ursi* , combats NAFLD by inhibiting ferroptosis both in vivo and in vitro. It increases SLC7A11 expression through suppression of FTO, leading to enhanced m^6^A methylation and stabilisation of SLC7A11 mRNA. Because SLC7A11 is a critical regulator of the glutathione‐dependent anti‐ferroptotic pathway, its upregulation prevents lipid peroxidation and ferroptosis, thereby alleviating NAFLD‐associated damage [258].

Unlike ischemic stroke, where therapeutic benefit is linked to suppression of ACSL4‐ or BECN1‐associated pathways, the protective effect in NAFLD is centred on reinforcement of SLC7A11‐dependent antioxidant defence.

### 5.3 Hepatic Ischemia/Reperfusion Injury (HIRI)

Nicotinamide mononucleotide (NMN), a precursor of NAD+, alleviates hepatic injury in older livers during ischemia/reperfusion by enhancing the demethylase activity of FTO [145]. This decreases m^6^A modification and suppresses ferroptosis, as reflected by reduced serum hepatic enzyme levels, improved histological features, and decreased apoptosis in treated mice. These findings indicate that NMN protects aged livers partly by reducing m^6^A‐dependent ferroptotic sensitivity during reperfusion injury [145].

Additionally, interferon‐α (IFN‐α) reduces hepatic ferroptosis by upregulating DHX58, which recruits YTHDC2 to enhance translation of GPX4, a central anti‐ferroptotic enzyme [144].

Together, these interventions indicate that therapeutic regulation in HIRI mainly restores GPX4‐centred antioxidant capacity, although the upstream m^6^A regulators differ.

### 5.4 Liver Fibrosis

Dihydroartemisinin (DHA) combats liver fibrosis by inducing ferroptosis in hepatic stellate cells, which are key drivers of extracellular matrix deposition during fibrosis [267]. DHA promotes autophagy in hepatic stellate cells through downregulation of FTO, increasing m^6^A modification on BECN1 mRNA. Stabilised BECN1 mRNA promotes autophagy, which then triggers ferroptosis. Silencing the m^6^A reader YTHDF1 or inhibiting autophagy disrupts DHA‐induced ferroptosis, indicating a tightly regulated process. In vivo, DHA alleviates fibrosis by triggering ferroptosis in hepatic stellate cells through m^6^A‐dependent pathways [267].

This therapeutic pattern differs from NAFLD and HIRI because the beneficial effect relies on inducing, rather than suppressing, ferroptosis. It also highlights that autophagy‐dependent ferroptosis may be advantageous in fibrotic disease but detrimental in parenchymal injury.

### 5.5 Doxorubicin‐Induced Cardiotoxicity

The METTL3‐specific inhibitor STM2457 alleviates cardiac damage caused by doxorubicin, as evidenced by improved left ventricular ejection fraction, reduced fibrosis, lower cardiac troponin T levels, and normalised heart morphology in mouse models [132]. STM2457 suppresses ferroptosis by increasing the glutathione redox ratio, reducing lipid peroxidation, and limiting cardiac iron overload. These findings highlight STM2457's potential to protect against doxorubicin‐induced cardiotoxicity by targeting ferroptosis [132].

These results support METTL3 inhibition as a cardioprotective strategy in settings where ferroptosis contributes directly to treatment‐related tissue injury.

### 5.6 Sepsis‐Associated Lung Injury

Septic lung injury, characterised by ferroptosis‐induced alveolar epithelial cell death, can be alleviated through targeted METTL3 inhibition in mice [154]. STM2457, a specific METTL3 inhibitor, reduces ferroptosis in septic mouse models by mitigating inflammation, lung edema, and fibrosis and by improving survival. Mechanistically, STM2457 suppresses lactate‐driven METTL3‐mediated m^6^A modification that stabilises ACSL4 mRNA, a key ferroptosis regulator [154].

Compared with cardiotoxicity, in which METTL3 inhibition limits iron overload and oxidative damage in cardiomyocytes, the therapeutic effect in sepsis‐associated lung injury is more closely linked to suppression of inflammatory, lactate‐driven ACSL4 stabilisation.

### 5.7 Osteoarthritis

Bone marrow mesenchymal stem cell‐derived exosomes reduce chondrocyte ferroptosis in osteoarthritis by targeting the METTL3‐regulated ACSL4 pathway. These exosomes inhibit m^6^A modification on ACSL4 mRNA, decreasing its stability and expression and thereby suppressing ferroptosis. In vivo, they alleviate osteoarthritis symptoms by attenuating cartilage degradation and preserving joint structure [260].

This finding illustrates that biologically derived delivery systems may modulate m^6^A‐dependent ferroptosis without requiring direct small‐molecule inhibition of m^6^A enzymes.

### 5.8 Endometrial Injury

Modified bone marrow mesenchymal stem cell exosomes carrying miR‐340‐3p enhance endometrial recovery by inhibiting ferroptosis in endometrial stromal cells [268]. These exosomes downregulate METTL3 expression, reduce m^6^A modification on HMOX1 mRNA, and stabilise its expression in injured endometrium [268]. The m^6^A reader YTHDF2 normally facilitates HMOX1 mRNA degradation, but this activity is suppressed in the presence of these exosomes. This mechanism alleviates endometrial fibrosis and improves regenerative outcomes in rat models of endometrial injury [268].

Like the exosomes used in osteoarthritis, these modified exosomes act through a regenerative, RNA‐centred intervention, but the downstream target differs, shifting from ACSL4 to HMOX1.

### 5.9 NSCLC

Arsenic trioxide inhibits stemness and enhances ferroptosis in lung adenocarcinoma stem cells by reducing ZC3H13‐mediated m^6^A modification and destabilising SLC7A11 mRNA. Silencing ZC3H13 further promotes ferroptosis and diminishes tumorigenicity, whereas ZC3H13 overexpression reverses these effects. The ability of arsenic trioxide to reduce cancer stem cell traits and induce ferroptosis highlights its therapeutic potential in NSCLC [262].

This antitumor effect differs from the protective strategies described above, as the desired outcome in cancer is to enhance ferroptotic sensitivity rather than suppress it.

### 5.10 CRC

A novel FTO inhibitor, mupirocin, inhibits colorectal cancer growth by inducing ferroptosis through downregulation of SLC7A11 and GPX4, key regulators of glutathione‐dependent antioxidant defense. Because FTO normally demethylates m^6^A‐modified mRNAs, its inhibition increases m^6^A modification and destabilises these ferroptosis‐related transcripts [215].

Similarly, curdione enhances ferroptosis by increasing METTL14 expression. METTL14 introduces m^6^A modification on SLC7A11 and SLC3A2 mRNAs, reducing their stability through YTHDF2‐mediated degradation. This lowers protein expression and triggers ferroptosis in colorectal cancer cells [263].

MK2206, an AKT inhibitor, induces ferroptosis by downregulating FTO and promoting m^6^A‐dependent degradation of GPX4 mRNA. YTHDF2 is required for this effect, and silencing YTHDF2 reverses MK2206‐induced ferroptosis. In addition, FB23‐2, another FTO inhibitor, similarly decreases GPX4 expression and further supports the role of FTO in ferroptosis regulation [264].

Together, these compounds show that colorectal cancer therapy can exploit m^6^A‐dependent destabilisation of SLC7A11‐, SLC3A2‐, and GPX4‐centred defence pathways, with FTO emerging as a recurrent therapeutic target [215, 263, 264].

### 5.11 Renal Cell Carcinoma (RCC)

Erianin, a low‐molecular‐weight bibenzyl natural product from Dendrobium chrysotoxum, exerts significant antitumor effects in renal cell carcinoma by inducing ferroptosis. Erianin increases METTL3 expression and decreases FTO expression, thereby enhancing m^6^A modification on key ferroptosis‐related genes such as ALOX12 and p53. These changes stabilise ALOX12 and p53 mRNAs, increase their half‐life and translation efficiency, promote lipid peroxidation, suppress SLC7A11, and sensitise cells to ferroptosis [265]. Erianin inhibits renal cancer cell proliferation, invasion, angiogenesis, and tumorigenesis in both in vitro and in vivo models, highlighting its therapeutic potential for RCC, particularly in cancer stem cells with strong ferroptosis resistance [265].

This strategy differs from CRC, where therapy mainly destabilises antioxidant transcripts because RCC intervention simultaneously enhances pro‐ferroptotic signalling and suppresses anti‐ferroptotic defences.

### 5.12 HCC

Metformin, an anti‐diabetic drug, modulates m^6^A modification by inhibiting METTL3 activity. This decreases m^6^A levels on PPARGC1A mRNA and stabilises its expression. PPARGC1A suppresses tumour progression by inhibiting WNT/β‐catenin and TGF‐β/SMAD signalling [209].

Although this study is not centred on a classical ferroptosis effector such as GPX4 or SLC7A11, it suggests that therapeutic modulation of m^6^A may alter tumour susceptibility through broader metabolic and signalling programs linked to ferroptosis regulation.

### 5.13 Acute Myeloid Leukaemia (AML)

In AML, ferroptosis induction offers a potential strategy to target leukaemia stem cells and chemoresistant populations. Licochalcone A inhibits cell proliferation and induces ferroptosis by suppressing IGF2BP3, an m^6^A reader protein that stabilises MDM2 mRNA. IGF2BP3‐mediated MDM2 upregulation promotes p53 degradation and suppresses ferroptosis. By downregulating this IGF2BP3‐dependent MDM2 regulatory pathway, licochalcone A restores p53 activity and sensitises AML cells to ferroptosis [266]. IGF2BP3 is overexpressed in AML specimens and cell lines, correlates with poor prognosis and treatment resistance, and its silencing inhibits AML cell viability while triggering ferroptosis by destabilising ferroptosis‐suppressive genes [266].

This mechanism differs from solid tumours that mainly depend on membrane antioxidant systems, as AML therapy here is centred on restoring p53 activity through reader‐dependent transcript destabilisation.

Together, these cumulative therapeutic studies indicate that m^6^A‐dependent ferroptosis can be manipulated in two opposite but clinically meaningful directions: (1) suppressing ferroptosis to protect normal tissues from ischemic, inflammatory, fibrotic, or (2) treatment‐related injury, and enhancing ferroptosis to overcome tumour survival and treatment resistance. The underlying therapeutic targets differ across diseases. Protective strategies most often restore GPX4, SLC7A11, HMOX1, or related antioxidant programs, whereas anticancer strategies more commonly destabilise ferroptosis‐resistance factors or enhance pro‐ferroptotic signalling. These distinctions further support the view that m^6^A‐dependent ferroptosis is highly context‐dependent and should be therapeutically modulated according to disease‐specific regulatory mechanisms.

## 6 Conclusions and Future Perspectives

Methylation of RNA, particularly m^6^A modification, represents the most prevalent reversible epigenetic regulation and has emerged as a key modulator of ferroptosis through post‐transcriptional control of ferroptosis‐related genes. Current evidence indicates that m^6^A‐dependent regulation converges on several core processes. These processes include antioxidant defence, iron metabolism, lipid peroxidation, and autophagy, thereby shaping ferroptotic susceptibility across diverse diseases. Importantly, these effects are not mediated through a uniform mechanism, as different disease contexts preferentially engage distinct m^6^A regulators and downstream targets. Targeting m^6^A as an upstream regulator of ferroptosis, therefore, represents a promising but inherently context‐dependent therapeutic strategy.

Despite rapid progress, the study of m^6^A‐mediated ferroptosis remains at a relatively early stage and is subject to several important limitations. First, the functional roles of m^6^A regulators, including writers, erasers, and readers, are not consistent across diseases, and in some cases exert opposing effects on ferroptosis depending on cellular context and target selection. Second, most current studies are confined to single‐disease or single‐cell‐type models, limiting cross‐context comparison and hindering the establishment of a unified mechanistic framework. Third, existing work is heavily centred on a limited set of canonical ferroptosis pathways, particularly the SLC7A11–GPX4 axis and ACSL4‐mediated lipid peroxidation, while alternative regulatory routes remain insufficiently explored. Fourth, the interaction between m^6^A‐dependent ferroptosis and other forms of regulated cell death, such as apoptosis, necroptosis, and autophagy, is still poorly defined. Finally, although small‐molecule inhibitors and biologically derived interventions targeting m^6^A regulators have shown therapeutic potential, their specificity, systemic effects, and translational applicability across different diseases remain uncertain.

From a translational perspective, targeting m^6^A‐dependent ferroptosis presents additional challenges. Ferroptosis itself plays dual and context‐dependent roles, being protective in certain cancers but detrimental in conditions such as ischemia, inflammation, and organ injury. Moreover, m^6^A regulators control broad transcriptomic programs beyond ferroptosis, raising concerns about off‐target effects associated with systemic modulation. Achieving tissue‐specific and pathway‐specific regulation of m^6^A‐dependent ferroptosis, therefore, represents a critical barrier for clinical application.

In summary, m^6^A RNA modifications function as pivotal but context‐dependent regulators of ferroptosis, contributing to disease progression through diverse and disease‐specific mechanisms. Therapeutic modulation of this axis holds substantial promise, but its successful translation will depend on a deeper mechanistic understanding of disease‐specific regulatory networks, improved precision in targeting m^6^A machinery, and integration of ferroptosis‐based strategies into broader therapeutic frameworks.

## Author Contributions

Lida Du, Shuang Wu, Yumin Wang and Hongquan Wang researched data for the article and contributed substantially to discussion of the content. Lida Du, Shuang Wu, Yumin Wang and Haitong Wang wrote the article. Joshua S. Fleishman, Haitong Wang, Yuan Zhou, Clara X. Wang and Lida Du reviewed and/or edited the manuscript before submission. Yumin Wang, Joshua S. Fleishman, and Hongquan Wang prepared figures. Yumin Wang, Lida Du and Haitong Wang conceived of and designed the study. Yumin Wang and Hongquan Wang provided administrative support. All authors analysed and interpreted the data. All authors read and approved the final manuscript.

## Funding

This work was supported by Beijing Natural Science Foundation (7252174), National Natural Science Foundation of China (82505717; 82501516), Wu Jieping Medical Foundation (320.6750.2024‐13‐59), Science Foundation of ASCH (YN202305, YN202402, YN202423), Science Foundation of AMHT (2022YK01, 2022YK27, 2024YK04, 2024YK05) and Grant of Chinese Medicine Education Association (MBRC0012025029; MBRC0012025011).

## Conflicts of Interest

The authors declare no conflicts of interest.

## Data Availability

Data sharing not applicable to this article as no datasets were generated or analysed during the current study.

## References

[1] B. R. Stockwell , J. P. Friedmann Angeli , H. Bayir , et al., “Ferroptosis: A Regulated Cell Death Nexus Linking Metabolism, Redox Biology, and Disease,” Cell 171 (2017): 273–285. 28985560 10.1016/j.cell.2017.09.021 PMC5685180

[2] B. R. Stockwell , “Ferroptosis Turns 10: Emerging Mechanisms, Physiological Functions, and Therapeutic Applications,” Cell 185, no. 14 (2022): 2401–2421. 35803244 10.1016/j.cell.2022.06.003 PMC9273022

[3] Y. Wang , X. Wu , Z. Ren , et al., “Overcoming Cancer Chemotherapy Resistance by the Induction of Ferroptosis,” Drug Resistance Updates 66 (2023): 100916. 36610291 10.1016/j.drup.2022.100916

[4] S. J. Dixon , K. M. Lemberg , M. R. Lamprecht , et al., “Ferroptosis: An Iron‐Dependent Form of Nonapoptotic Cell Death,” Cell 149, no. 5 (2012): 1060–1072. 22632970 10.1016/j.cell.2012.03.042 PMC3367386

[5] L. Galluzzi , I. Vitale , S. A. Aaronson , et al., “Molecular Mechanisms of Cell Death: Recommendations of the Nomenclature Committee on Cell Death 2018,” Cell Death and Differentiation 25 (2018): 486–541. 29362479 10.1038/s41418-017-0012-4 PMC5864239

[6] G. Lei , L. Zhuang , and B. Gan , “Targeting Ferroptosis as a Vulnerability in Cancer,” Nature Reviews. Cancer 22, no. 7 (2022): 381–396. 35338310 10.1038/s41568-022-00459-0 PMC10243716

[7] X. Chen , J. Li , R. Kang , D. J. Klionsky , and D. Tang , “Ferroptosis: Machinery and Regulation,” Autophagy 17, no. 9 (2021): 2054–2081. 32804006 10.1080/15548627.2020.1810918 PMC8496712

[8] X. Chen , R. Kang , G. Kroemer , and D. Tang , “Organelle‐Specific Regulation of Ferroptosis,” Cell Death and Differentiation 28 (2021): 2843–2856. 34465893 10.1038/s41418-021-00859-z PMC8481335

[9] Y. Wang , S. Wu , Q. Li , H. Sun , and H. Wang , “Pharmacological Inhibition of Ferroptosis as a Therapeutic Target for Neurodegenerative Diseases and Strokes,” Advanced Science 10 (2023): e2300325. 37341302 10.1002/advs.202300325 PMC10460905

[10] M. Ou , Y. Jiang , Y. Ji , et al., “Role and Mechanism of Ferroptosis in Neurological Diseases,” Molecular Metabolism 61 (2022): 101502. 35447365 10.1016/j.molmet.2022.101502 PMC9170779

[11] J. Lei , Z. Chen , S. Song , C. Sheng , S. Song , and J. Zhu , “Insight Into the Role of Ferroptosis in Non‐Neoplastic Neurological Diseases,” Frontiers in Cellular Neuroscience 14 (2020): 231. 32848622 10.3389/fncel.2020.00231 PMC7424047

[12] J. X. Ren , X. Sun , X. L. Yan , Z. N. Guo , and Y. Yang , “Ferroptosis in Neurological Diseases,” Frontiers in Cellular Neuroscience 14 (2020): 218. 32754017 10.3389/fncel.2020.00218 PMC7370841

[13] M. Y. Yao , T. Liu , L. Zhang , M. J. Wang , Y. Yang , and J. Gao , “Role of Ferroptosis in Neurological Diseases,” Neuroscience Letters 747 (2021): 135614. 33485988 10.1016/j.neulet.2020.135614

[14] N. Li , W. Jiang , W. Wang , R. Xiong , X. Wu , and Q. Geng , “Ferroptosis and Its Emerging Roles in Cardiovascular Diseases,” Pharmacological Research 166 (2021): 105466. 33548489 10.1016/j.phrs.2021.105466

[15] X. Fang , H. Ardehali , J. Min , and F. Wang , “The Molecular and Metabolic Landscape of Iron and Ferroptosis in Cardiovascular Disease,” Nature Reviews. Cardiology 20 (2023): 7–23. 35788564 10.1038/s41569-022-00735-4 PMC9252571

[16] X. Wu , Y. Li , S. Zhang , and X. Zhou , “Ferroptosis as a Novel Therapeutic Target for Cardiovascular Disease,” Theranostics 11 (2021): 3052–3059. 33537073 10.7150/thno.54113 PMC7847684

[17] Y. Yu , Y. Yan , F. Niu , et al., “Ferroptosis: A Cell Death Connecting Oxidative Stress, Inflammation and Cardiovascular Diseases,” Cell Death Discovery 7 (2021): 193. 34312370 10.1038/s41420-021-00579-w PMC8313570

[18] B. Huang , H. Wang , S. Liu , et al., “Palmitoylation‐Dependent Regulation of GPX4 Suppresses Ferroptosis,” Nature Communications 16 (2025): 867. 10.1038/s41467-025-56344-5 PMC11746948 39833225

[19] L. Yang , L. M. Cao , X. J. Zhang , and B. Chu , “Targeting Ferroptosis as a Vulnerability in Pulmonary Diseases,” Cell Death & Disease 13 (2022): 649. 35882850 10.1038/s41419-022-05070-7 PMC9315842

[20] Y. Li , Y. Yang , and Y. Yang , “Multifaceted Roles of Ferroptosis in Lung Diseases,” Frontiers in Molecular Biosciences 9 (2022): 919187. 35813823 10.3389/fmolb.2022.919187 PMC9263225

[21] X. Wei , X. Li , S. Hu , J. Cheng , and R. Cai , “Regulation of Ferroptosis in Lung Adenocarcino,” International Journal of Molecular Sciences 24 (2023): 14614.37834062

[22] W. Xu , H. Deng , S. Hu , et al., “Role of Ferroptosis in Lung Diseases,” Journal of Inflammation Research 14 (2021): 2079–2090. 34045882 10.2147/JIR.S307081 PMC8144020

[23] G. Guo , W. Yang , C. Sun , and X. Wang , “Dissecting the Potential Role of Ferroptosis in Liver Diseases: An Updated Review,” Free Radical Research 57, no. 4 (2023): 282–293. 37401821 10.1080/10715762.2023.2232941

[24] X. Wang , Y. Zhou , J. Min , and F. Wang , “Zooming in and Out of Ferroptosis in Human Disease,” Frontiers in Medicine 17 (2023): 173–206. 10.1007/s11684-023-0992-z 37121959

[25] R. Guo , J. Duan , S. Pan , et al., “The Road From AKI to CKD: Molecular Mechanisms and Therapeutic Targets of Ferroptosis,” Cell Death & Disease 14 (2023): 426. 37443140 10.1038/s41419-023-05969-9 PMC10344918

[26] J. Guo , B. Xu , Q. Han , et al., “Ferroptosis: A Novel Anti‐Tumor Action for Cisplatin,” Cancer Research and Treatment 50 (2018): 445–460. 28494534 10.4143/crt.2016.572 PMC5912137

[27] X. Chen , R. Kang , G. Kroemer , and D. Tang , “Broadening Horizons: The Role of Ferroptosis in Cancer,” Nature Reviews. Clinical Oncology 18, no. 5 (2021): 280–296. 10.1038/s41571-020-00462-0 33514910

[28] C. Zhang , X. Liu , S. Jin , Y. Chen , and R. Guo , “Ferroptosis in Cancer Therapy: A Novel Approach to Reversing Drug Resistance,” Molecular Cancer 21 (2022): 47. 35151318 10.1186/s12943-022-01530-y PMC8840702

[29] Y. Wang , J. Hu , J. S. Fleishman , et al., “Inducing Ferroptosis by Traditional Medicines: A Novel Approach to Reverse Chemoresistance in Lung Cancer,” Frontiers in Pharmacology 15 (2024): 1290183. 38855750 10.3389/fphar.2024.1290183 PMC11158628

[30] M. Gao , P. Monian , N. Quadri , R. Ramasamy , and X. Jiang , “Glutaminolysis and Transferrin Regulate Ferroptosis,” Molecular Cell 59, no. 2 (2015): 298–308. 26166707 10.1016/j.molcel.2015.06.011 PMC4506736

[31] M. Gao , P. Monian , Q. Pan , W. Zhang , J. Xiang , and X. Jiang , “Ferroptosis Is an Autophagic Cell Death Process,” Cell Research 26, no. 9 (2016): 1021–1032. 27514700 10.1038/cr.2016.95 PMC5034113

[32] W. Hou , Y. Xie , X. Song , et al., “Autophagy Promotes Ferroptosis by Degradation of Ferritin,” Autophagy 12, no. 8 (2016): 1425–1428. 27245739 10.1080/15548627.2016.1187366 PMC4968231

[33] V. E. Kagan , G. Mao , F. Qu , et al., “Oxidized Arachidonic and Adrenic PEs Navigate Cells to Ferroptosis,” Nature Chemical Biology 13, no. 1 (2017): 81–90. 27842066 10.1038/nchembio.2238 PMC5506843

[34] S. Doll , B. Proneth , Y. Y. Tyurina , et al., “ACSL4 Dictates Ferroptosis Sensitivity by Shaping Cellular Lipid Composition,” Nature Chemical Biology 13 (2017): 91–98. 27842070 10.1038/nchembio.2239 PMC5610546

[35] R. Shah , M. S. Shchepinov , and D. A. Pratt , “Resolving the Role of Lipoxygenases in the Initiation and Execution of Ferroptosis,” ACS Central Science 4, no. 3 (2018): 387–396. 29632885 10.1021/acscentsci.7b00589 PMC5879472

[36] W. S. Yang , K. J. Kim , M. M. Gaschler , M. Patel , M. S. Shchepinov , and B. R. Stockwell , “Peroxidation of Polyunsaturated Fatty Acids by Lipoxygenases Drives Ferroptosis,” Proceedings. National Academy of Sciences. United States of America 113, no. 34 (2016): E4966–E4975. 10.1073/pnas.1603244113 PMC5003261 27506793

[37] Y. Zou , H. Li , E. T. Graham , et al., “Cytochrome P450 Oxidoreductase Contributes to Phospholipid Peroxidation in Ferroptosis,” Nature Chemical Biology 16 (2020): 302–309. 32080622 10.1038/s41589-020-0472-6 PMC7353921

[38] B. Yan , Y. Ai , Q. Sun , et al., “Membrane Damage During Ferroptosis Is Caused by Oxidation of Phospholipids Catalyzed by the Oxidoreductases POR and CYB5R1,” Molecular Cell 81 (2021): 355–369.e10. 33321093 10.1016/j.molcel.2020.11.024

[39] J. P. Friedmann Angeli , M. Schneider , B. Proneth , et al., “Inactivation of the Ferroptosis Regulator Gpx4 Triggers Acute Renal Failure in Mice,” Nature Cell Biology 16 (2014): 1180–1191. 25402683 10.1038/ncb3064 PMC4894846

[40] I. Ingold , C. Berndt , S. Schmitt , et al., “Selenium Utilization by GPX4 is Required to Prevent Hydroperoxide‐Induced Ferroptosis,” Cell 172 (2018): 409. 29290465 10.1016/j.cell.2017.11.048

[41] W. S. Yang , R. SriRamaratnam , M. E. Welsch , et al., “524 BR Stockwell, Regulation of Ferroptotic Cancer Cell Death by GPX4,” Cell 156 (2014): 317. 24439385 10.1016/j.cell.2013.12.010 PMC4076414

[42] H. Sato , M. Tamba , T. Ishii , and S. Bannai , “Cloning and Expression of a Plasma Membrane Cystine/Glutamate Exchange Transporter Composed of Two Distinct Proteins,” Journal of Biological Chemistry 274 (1999): 11455. 10206947 10.1074/jbc.274.17.11455

[43] K. Bersuker , J. M. Hendricks , Z. Li , et al., “The CoQ Oxidoreductase FSP1 Acts Parallel to GPX4 to Inhibit Ferroptosis,” Nature 575 (2019): 688–692. 31634900 10.1038/s41586-019-1705-2 PMC6883167

[44] S. Doll , F. P. Freitas , R. Shah , et al., “FSP1 Is a Glutathione‐Independent Ferroptosis Suppressor,” Nature 575 (2019): 693–698. 31634899 10.1038/s41586-019-1707-0

[45] V. Kraft , C. T. Bezjian , S. Pfeiffer , et al., “GTP Cyclohydrolase 1/Tetrahydrobiopterin Counteract Ferroptosis Through Lipid Remodeling,” ACS Central Science 6, no. 1 (2020): 41–53. 31989025 10.1021/acscentsci.9b01063 PMC6978838

[46] C. Mao , X. Liu , Y. Zhang , et al., “DHODH‐Mediated Ferroptosis Defence Is a Targetable Vulnerability in Cancer,” Nature 593 (2021): 586–590. 33981038 10.1038/s41586-021-03539-7 PMC8895686

[47] D. Liang , Y. Feng , F. Zandkarimi , et al., “Ferroptosis Surveillance Independent of GPX4 and Differentially Regulated by Sex Hormones,” Cell 186, no. 13 (2023): 2748–2764.e22. 37267948 10.1016/j.cell.2023.05.003 PMC10330611

[48] F. P. Freitas , H. Alborzinia , A. F. Dos Santos , et al., “7‐Dehydrocholesterol Is an Endogenous Suppressor of Ferroptosis,” Nature 626 (2024): 401–410, 10.1038/s41586-023-06878-9. 38297129

[49] Y. Li , Q. Ran , Q. Duan , et al., “7‐Dehydrocholesterol Dictates Ferroptosis Sensitivity,” Nature 626 (2024): 411–418, 10.1038/s41586-023-06983-9. 38297130 PMC11298758

[50] P. Wang , K. A. Doxtader , and Y. Nam , “Structural Basis for Cooperative Function of Mettl3 and Mettl14 Methyltransferases,” Molecular Cell 63, no. 2 (2016): 306–317. 27373337 10.1016/j.molcel.2016.05.041 PMC4958592

[51] X. Wang , J. Feng , Y. Xue , et al., “Structural Basis of N(6)‐Adenosine Methylation by the METTL3‐METTL14 Complex,” Nature 534 (2016): 575–578. 27281194 10.1038/nature18298

[52] R. Wu , A. Li , B. Sun , et al., “A Novel m(6)A Reader Prrc2a Controls Oligodendroglial Specification and Myelination,” Cell Research 29 (2019): 23–41, 10.1038/s41422-018-0113-8. 30514900 PMC6318280

[53] I. A. Roundtree , G. Z. Luo , Z. Zhang , et al., “YTHDC1 Mediates Nuclear Export of N(6)‐Methyladenosine Methylated mRNAs,” eLife 6 (2017): e31311. 28984244 10.7554/eLife.31311 PMC5648532

[54] J. A. Bokar , M. E. Shambaugh , D. Polayes , A. G. Matera , and F. M. Rottman , “Purification and cDNA Cloning of the AdoMet‐Binding Subunit of the Human mRNA (N6‐Adenosine)‐Methyltransferase,” RNA 3, no. 11 (1997): 1233–1247. 9409616 PMC1369564

[55] D. P. Patil , C. K. Chen , B. F. Pickering , et al., “M(6)A RNA Methylation Promotes XIST‐Mediated Transcriptional Repression,” Nature 537, no. 7620 (2016): 369–373, 10.1038/nature19342. 27602518 PMC5509218

[56] K. E. Pendleton , B. Chen , K. Liu , et al., “The U6 snRNA m6A Methyltransferase METTL16 Regulates SAM Synthetase Intron Retention,” Cell 169, no. 5 (2017): 824–835.e14. 28525753 10.1016/j.cell.2017.05.003 PMC5502809

[57] E. R. Satterwhite and K. D. Mansfield , “RNA Methyltransferase METTL16: Targets and Function,” Wiley Interdiscip Rev RNA 13, no. 2 (2022): e1681. 34227247 10.1002/wrna.1681 PMC9286414

[58] G. Jia , Y. Fu , X. Zhao , et al., “N6‐Methyladenosine in Nuclear RNA Is a Major Substrate of the Obesity‐Associated FTO,” Nature Chemical Biology 7, no. 12 (2011): 885–887. 22002720 10.1038/nchembio.687 PMC3218240

[59] G. Zheng , J. A. Dahl , Y. Niu , et al., “ALKBH5 Is a Mammalian RNA Demethylase That Impacts RNA Metabolism and Mouse Fertility,” Molecular Cell 49, no. 1 (2013): 18–29. 23177736 10.1016/j.molcel.2012.10.015 PMC3646334

[60] Y. Ueda , I. Ooshio , Y. Fusamae , et al., “AlkB Homolog 3‐Mediated tRNA Demethylation Promotes Protein Synthesis in Cancer Cells,” Scientific Reports 7 (2017): 42271. 28205560 10.1038/srep42271 PMC5304225

[61] J. Mauer , X. Luo , A. Blanjoie , et al., “Reversible Methylation of m6Am in the 5′ Cap Controls mRNA Stability,” Nature 541, no. 7637 (2017): 371–375. 28002401 10.1038/nature21022 PMC5513158

[62] W. Xiao , S. Adhikari , U. Dahal , et al., “Nuclear m(6)A Reader YTHDC1 Regulates mRNA Splicing,” Molecular Cell 61 (2016): 507–519. 26876937 10.1016/j.molcel.2016.01.012

[63] F. Zhang , Y. Kang , M. Wang , et al., “Fragile X Mental Retardation Protein Modulates the Stability of Its m6A‐Marked Messenger RNA Targets,” Human Molecular Genetics 27 (2018): 3936–3950. 30107516 10.1093/hmg/ddy292 PMC6216232

[64] X. Jiang , B. Liu , Z. Nie , et al., “The Role of m6A Modification in the Biological Functions and Diseases,” Signal Transduction and Targeted Therapy 6 (2021): 74, 10.1038/s41392-020-00450-x. 33611339 PMC7897327

[65] N. Liu , K. I. Zhou , M. Parisien , Q. Dai , L. Diatchenko , and T. Pan , “N6‐Methyladenosine Alters RNA Structure to Regulate Binding of a Low‐Complexity Protein,” Nucleic Acids Research 45 (2017): 6051–6063, 10.1093/nar/gkx141. 28334903 PMC5449601

[66] J. Choe , S. Lin , W. Zhang , et al., “mRNA Circularization by METTL3‐eIF3h Enhances Translation and Promotes Oncogenesis,” Nature 561 (2018): 556–560, 10.1038/s41586-018-0538-8. 30232453 PMC6234840

[67] C. J. David , M. Chen , M. Assanah , P. Canoll , and J. L. Manley , “HnRNP Proteins Controlled by c‐Myc Deregulate Pyruvate Kinase mRNA Splicing in Cancer,” Nature 463 (2010): 364–368, 10.1038/nature08697. 20010808 PMC2950088

[68] J. König , K. Zarnack , G. Rot , et al., “iCLIP Reveals the Function of hnRNP Particles in Splicing at Individual Nucleotide Resolution,” Nature Structural & Molecular Biology 17 (2010): 909–915, 10.1038/nsmb.1838. PMC3000544 20601959

[69] J. Liu , Y. Yue , D. Han , et al., “A METTL3‐METTL14 Complex Mediates Mammalian Nuclear RNA N6‐Adenosine Methylation,” Nature Chemical Biology 10, no. 2 (2014): 93–95. 24316715 10.1038/nchembio.1432 PMC3911877

[70] X. L. Ping , B. F. Sun , L. Wang , et al., “Mammalian WTAP Is a Regulatory Subunit of the RNA N6‐Methyladenosine Methyltransferase,” Cell Research 24, no. 2 (2014): 177–189. 24407421 10.1038/cr.2014.3 PMC3915904

[71] S. Schwartz , M. R. Mumbach , M. Jovanovic , et al., “Perturbation of m6A Writers Reveals Two Distinct Classes of mRNA Methylation at Internal and 5′ Sites,” Cell Reports 8, no. 1 (2014): 284–296, 10.1016/j.celrep.2014.05.048. 24981863 PMC4142486

[72] Y. Yue , J. Liu , X. Cui , et al., “VIRMA Mediates Preferential m6A mRNA Methylation in 3'UTR and Near Stop Codon and Associates With Alternative Polyadenylation,” Cell Discovery 410 (2018): 10, 10.1038/s41421-018-0019-0. PMC5826926 29507755

[73] K. E. Pendleton , B. Chen , K. Liu , et al., “The U6 snRNA m(6)A Methyltransferase METTL16 Regulates SAM Synthetase Intron Retention,” Cell 169 (2017): 824–835.e14, 10.1016/j.cell.2017.05.003. 28525753 PMC5502809

[74] A. S. Warda , J. Kretschmer , P. Hackert , et al., “Human METTL16 Is a N(6)‐Methyladenosine (m(6)A) Methyltransferase That Targets Pre‐mRNAs and Various Non‐Coding RNAs,” EMBO Reports 18 (2017): 2004–2014, 10.15252/embr.201744940. 29051200 PMC5666602

[75] P. Knuckles , T. Lence , I. U. Haussmann , et al., “Zc3h13/Flacc Is Required for Adenosine Methylation by Bridging the mRNA‐Binding Factor Rbm15/Spenito to the m(6)A Machinery Component Wtap/Fl(2)d,” Genes & Development 32, no. 5–6 (2018): 415–429, 10.1101/gad.309146.117. 29535189 PMC5900714

[76] J. Wen , R. Lv , H. Ma , et al., “Zc3h13 Regulates Nuclear RNA m(6)A Methylation and Mouse Embryonic Stem Cell Self‐Renewal,” Molecular Cell 69, no. 6 (2018): 1028–1038.e6, 10.1016/j.molcel.2018.02.015. 29547716 PMC5858226

[77] N. van Tran , F. Ernst , B. R. Hawley , et al., “The Human 18S rRNA m6A Methyltransferase METTL5 Is Stabilized by TRMT112,” Nucleic Acids Research 47 (2019): 7719–7733, 10.1093/nar/gkz619. 31328227 PMC6735865

[78] H. Ma , X. Wang , J. Cai , et al., “N(6‐)Methyladenosine Methyltransferase ZCCHC4 Mediates Ribosomal RNA Methylation,” Nature Chemical Biology 15 (2019): 88–94, 10.1038/s41589-018-0184-3. 30531910 PMC6463480

[79] R. Pinto , C. B. Vågbø , M. E. Jakobsson , et al., “The Human Methyltransferase ZCCHC4 Catalyses N6‐Methyladenosine Modification of 28S Ribosomal RNA,” Nucleic Acids Research 48 (2020): 830–846, 10.1093/nar/gkz1147. 31799605 PMC6954407

[80] W. Ren , J. Lu , M. Huang , et al., “Structure and Regulation of ZCCHC4 in m(6)A‐Methylation of 28S rRNA,” Nature Communications 10 (2019): 5042, 10.1038/s41467-019-12923-x. PMC6834594 31695039

[81] H. Chen , L. Gu , E. A. Orellana , et al., “METTL4 Is an snRNA m(6)Am Methyltransferase That Regulates RNA Splicing,” Cell Research 30 (2020): 544–547. 31913360 10.1038/s41422-019-0270-4 PMC7264358

[82] H. Du , Y. Zhao , J. He , et al., “YTHDF2 Destabilizes m(6)A‐Containing RNA Through Direct Recruitment of the CCR4‐NOT Deadenylase Complex,” Nature Communications 7 (2016): 12626. 10.1038/ncomms12626 PMC5007331 27558897

[83] X. Wang , Z. Lu , A. Gomez , et al., “N6‐Methyladenosine‐Dependent Regulation of Messenger RNA Stability,” Nature 505 (2014): 117–120, 10.1038/nature12730. 24284625 PMC3877715

[84] X. Wang , B. S. Zhao , I. A. Roundtree , et al., “N(6)‐Methyladenosine Modulates Messenger RNA Translation Efficiency,” Cell 161 (2015): 1388–1399, 10.1016/j.cell.2015.05.014. 26046440 PMC4825696

[85] K. D. Meyer , D. P. Patil , J. Zhou , et al., “5' UTR m(6)A Promotes Cap‐Independent Translation,” Cell 163 (2015): 999–1010, 10.1016/j.cell.2015.10.012. 26593424 PMC4695625

[86] C. R. Alarcón , H. Goodarzi , H. Lee , X. Liu , S. Tavazoie , and S. F. Tavazoie , “HNRNPA2B1 Is a Mediator of m(6)A‐Dependent Nuclear RNA Processing Events,” Cell 162 (2015): 1299–1308, 10.1016/j.cell.2015.08.011. 26321680 PMC4673968

[87] N. Liu , Q. Dai , G. Zheng , C. He , M. Parisien , and T. Pan , “N(6)‐Methyladenosine‐Dependent RNA Structural Switches Regulate RNA‐Protein Interactions,” Nature 518 (2015): 560–564, 10.1038/nature14234. 25719671 PMC4355918

[88] A. Li , Y. S. Chen , X. L. Ping , et al., “Cytoplasmic m(6)A Reader YTHDF3 Promotes mRNA Translation,” Cell Research 27, no. 3 (2017): 444–447. 28106076 10.1038/cr.2017.10 PMC5339832

[89] H. Shi , X. Wang , Z. Lu , et al., “YTHDF3 Facilitates Translation and Decay of N(6)‐Methyladenosine‐Modified RNA,” Cell Research 27 (2017): 315–328, 10.1038/cr.2017.15. 28106072 PMC5339834

[90] P. J. Hsu , Y. Zhu , H. Ma , et al., “Ythdc2 Is an N(6)‐Methyladenosine Binding Protein That Regulates Mammalian Spermatogenesis,” Cell Research 27 (2017): 1115–1127. 28809393 10.1038/cr.2017.99 PMC5587856

[91] H. Huang , H. Weng , W. Sun , et al., “Recognition of RNA N(6)‐Methyladenosine by IGF2BP Proteins Enhances mRNA Stability and Translation,” Nature Cell Biology 20 (2018): 285–295. 29476152 10.1038/s41556-018-0045-z PMC5826585

[92] B. M. Edens , C. Vissers , J. Su , et al., “FMRP Modulates Neural Differentiation Through m(6)A‐Dependent mRNA Nuclear Export,” Cell Reports 28 (2019): 845–854.e5. 31340148 10.1016/j.celrep.2019.06.072 PMC6687293

[93] F. Yu , A. C. Zhu , S. Liu , et al., “RBM33 Is a Unique m(6)A RNA‐Binding Protein That Regulates ALKBH5 Demethylase Activity and Substrate Selectivity,” Molecular Cell 83 (2023): 2003–2019. 37257451 10.1016/j.molcel.2023.05.010 PMC10330838

[94] N. Wang , T. Ma , and B. Yu , “Targeting Epigenetic Regulators to Overcome Drug Resistance in Cancers,” Signal Transduction and Targeted Therapy 8, no. 1 (2023): 69. 36797239 10.1038/s41392-023-01341-7 PMC9935618

[95] S. Zhou , J. Liu , A. Wan , Y. Zhang , and X. Qi , “Epigenetic Regulation of Diverse Cell Death Modalities in Cancer: A Focus on Pyroptosis, Ferroptosis, Cuproptosis, and Disulfidptosis,” Journal of Hematology & Oncology 17 (2024): 22. 38654314 10.1186/s13045-024-01545-6 PMC11040947

[96] L. Garcia‐Martinez , Y. Zhang , Y. Nakata , H. L. Chan , and L. Morey , “Epigenetic Mechanisms in Breast Cancer Therapy and Resistance,” Nature Communications 12 (2021): 1786. 10.1038/s41467-021-22024-3 PMC7979820 33741974

[97] C. Ling and T. Rönn , “Epigenetics in Human Obesity and Type 2 Diabetes,” Cell Metabolism 29 (2019): 1028–1044. 30982733 10.1016/j.cmet.2019.03.009 PMC6509280

[98] F. Shu , H. Xiao , Q. N. Li , et al., “Epigenetic and Post‐Translational Modifications in Autophagy: Biological Functions and Therapeutic Targets,” Signal Transduction and Targeted Therapy 8 (2023): 32. 36646695 10.1038/s41392-022-01300-8 PMC9842768

[99] G. Cavalli and E. Heard , “Advances in Epigenetics Link Genetics to the Environment and Disease,” Nature 571, no. 7766 (2019): 489–499. 31341302 10.1038/s41586-019-1411-0

[100] J. Cao and Q. Yan , “Cancer Epigenetics, Tumor Immunity, and Immunotherapy,” Trends Cancer 6, no. 7 (2020): 580–592. 32610068 10.1016/j.trecan.2020.02.003 PMC7330177

[101] X. Tang , M. Guo , Y. Zhang , J. Lv , C. Gu , and Y. Yang , “Examining the Evidence for Mutual Modulation Between m6A Modification and Circular RNAs: Current Knowledge and Future Prospects,” Journal of Experimental & Clinical CANCER Research 43 (2024): 216. 39095902 10.1186/s13046-024-03136-2 PMC11297759

[102] R. Mehmood , “Ramifications of m6A Modification on ncRNAs in Cancer,” Current Genomics 25 (2024): 158–170. 39087001 10.2174/0113892029296712240405053201 PMC11288162

[103] W. Li , Y. Liu , R. Xu , et al., “ m6A Modification in Cardiovascular Disease: With a Focus on Programmed Cell Death,” Genes & Diseases 11, no. 5 (2024): 101039. 38988324 10.1016/j.gendis.2023.05.023 PMC11233881

[104] W. W. Liu , S. Q. Zheng , T. Li , et al., “RNA Modifications in Cellular Metabolism: Implications for Metabolism‐Targeted Therapy and Immunotherapy,” Signal Transduction and Targeted Therapy 9 (2024): 70. 38531882 10.1038/s41392-024-01777-5 PMC10966055

[105] Q. Liu , L. Lv , X. Cai , et al., “Correlation Between RNA N6‐Methyladenosine and Ferroptosis in Cancer: Current Status and Prospects,” Frontiers in Cell and Development Biology 12 (2024): 1252064. 10.3389/fcell.2024.1252064 PMC10976581 38550378

[106] Q. Su , L. Wu , C. Zheng , et al., “ALKBH5‐Mediated N6‐Methyladenosine Modification of HO‐1 mRNA Regulates Ferroptosis in Cobalt‐Induced Neurodegenerative Damage,” Environment International 190 (2024): 108897. 39047545 10.1016/j.envint.2024.108897

[107] X. Gu , Y. Song , X. Liu , Z. Cheng , J. Min , and Y. Zhang , “METTL14‐Mediated m6A Modification of TUG1 Represses Ferroptosis in Alzheimer's Disease via Inhibiting GDF15 Ubiquitination,” Frontiers in Bioscience‐Landmark 29 (2024): 298. 10.31083/j.fbl2908298 39206905

[108] P. Pang , S. Zhang , X. Fan , and S. Zhang , “Knockdown of Fat Mass and Obesity Alleviates the Ferroptosis in Parkinson's Disease Through m6A‐NRF2‐Dependent Manner,” Cell Biology International 48 (2024): 431–439. 38180302 10.1002/cbin.12118

[109] J. Feng , P. Zhang , K. Chen , et al., “Soot Nanoparticles Promote Ferroptosis in Dopaminergic Neurons via Alteration of m6A RNA Methylation in Parkinson's Disease,” Journal of Hazardous Materials 473 (2024): 134691. 38788584 10.1016/j.jhazmat.2024.134691

[110] W. Su , X. Yu , S. Wang , X. Wang , Z. Dai , and Y. Li , “METTL3 Regulates TFRC Ubiquitination and Ferroptosis Through Stabilizing NEDD4L mRNA to Impact Stroke,” Cell Biology and Toxicology 40 (2024): 8. 38302612 10.1007/s10565-024-09844-x PMC10834616

[111] Y. Zhang and X. Gong , “Fat Mass and Obesity Associated Protein Inhibits Neuronal Ferroptosis via the FYN/Drp1 Axis and Alleviate Cerebral Ischemia/Reperfusion Injury,” CNS Neuroscience & Therapeutics 30, no. 3 (2024): e14636. 38430221 10.1111/cns.14636 PMC10908355

[112] H. Ma , D. Ye , Y. Liu , et al., “Propofol Suppresses OGD/R‐Induced Ferroptosis in Neurons by Inhibiting the HIF‐1α/YTHDF1/BECN1 Axis,” Brain Injury 37 (2023): 1285–1293. 37614036 10.1080/02699052.2023.2237881

[113] Z. Zhang , Z. Zheng , and Y. Chen , “Mechanism of USP18‐Mediated NCOA4 m6A Modification via Maintaining FTO Stability in Regulating Ferritinophagy‐Mediated Ferroptosis in Cerebral Ischemia‐Reperfusion Injury,” Molecular Neurobiology 62 (2024): 3848–3862. 39331352 10.1007/s12035-024-04494-w

[114] Q. Peng , Y. Deng , Z. Xu , et al., “Fat Mass and Obesity‐Associated Protein Alleviates Cerebral Ischemia/Reperfusion Injury by Inhibiting Ferroptosis via miR‐320‐3p/SLC7A11 Axis,” Biochemical Pharmacology 230, no. Pt 2 (2024): 116603. 39486461 10.1016/j.bcp.2024.116603

[115] J. Li , C. Zou , Z. Zhang , and F. Xue , “N(6)‐Methyladenosine (m(6)A) Reader YTHDF2 Accelerates Endothelial Cells Ferroptosis in Cerebrovascular Atherosclerosis,” Molecular and Cellular Biochemistry 479 (2024): 1853–1861. 37792239 10.1007/s11010-023-04858-1

[116] L. Zhang , X. Wang , W. Che , S. Zhou , and Y. Feng , “METTL3 Silenced Inhibited the Ferroptosis Development via Regulating the TFRC Levels in the Intracerebral Hemorrhage Progression,” Brain Research 1811 (2023): 148373. 37105375 10.1016/j.brainres.2023.148373

[117] L. Mao , J. You , M. Xie , Y. Hu , and Q. Zhou , “Arginine Methylation of β‐Catenin Induced by PRMT2 Aggravates LPS‐Induced Cognitive Dysfunction and Depression‐Like Behaviors by Promoting Ferroptosis,” Molecular Neurobiology 61, no. 10 (2024): 7796–7813. 38430350 10.1007/s12035-024-04019-5

[118] Y. Chen and J. Huang , “FTO‐Mediated m6A Modification of FTH1 Inhibits Ferroptosis of Neurons in Neonatal Cerebral Hypoxic Ischemia,” Critical Reviews in Eukaryotic Gene Expression 34 (2024): 47–57. 39180207 10.1615/CritRevEukaryotGeneExpr.2024054011

[119] S. An , J. Shi , J. Huang , Z. Li , M. Feng , and G. Cao , “HIF‐1α‐Induced Upregulation of m6A Reader IGF2BP1 Facilitates Peripheral Nerve Injury Recovery by Enhancing SLC7A11 mRNA Stabilization,” In Vitro Cellular & Developmental Biology. Animal 59, no. 8 (2023): 596–605. 37783915 10.1007/s11626-023-00812-z

[120] J. Zhou , J. Tang , C. Zhang , et al., “ALKBH5 Targets ACSL4 mRNA Stability to Modulate Ferroptosis in Hyperbilirubinemia‐Induced Brain Damage,” Free Radical Biology & Medicine 220 (2024): 271–287. 38734267 10.1016/j.freeradbiomed.2024.05.014

[121] Q. Z. Tuo , S. T. Zhang , and P. Lei , “Mechanisms of Neuronal Cell Death in Ischemic Stroke and Their Therapeutic Implications,” Medicinal Research Reviews 42 (2022): 259–305. 33957000 10.1002/med.21817

[122] J. Guo , Q. Z. Tuo , and P. Lei , “Iron, Ferroptosis, and Ischemic Stroke,” Journal of Neurochemistry 165 (2023): 487–520. 36908209 10.1111/jnc.15807

[123] S. Y. Xu , S. M. Ni , C. L. Zeng , and Y. J. Peng , “Role of Ferroptosis in Glial Cells After Ischemic Stroke,” Front Biosci (Landmark Ed) 28 (2023): 208. 37796699 10.31083/j.fbl2809208

[124] Z. Zhang , Z. Zheng , Y. Chen , X. Niu , T. Ouyang , and D. Wang , “Mechanism of USP18‐Mediated NCOA4 m6A Modification via Maintaining FTO Stability in Regulating Ferritinophagy‐Mediated Ferroptosis in Cerebral Ischemia‐Reperfusion Injury,” Molecular Neurobiology 62, no. 3 (2025): 3848–3862. 39331352 10.1007/s12035-024-04494-w

[125] J. Zhou , S. Liao , C. Zhang , J. Luo , G. Li , and H. Li , “Expression Profiling of N6‐Methyladenosine‐Modified mRNA in PC12 Cells in Response to Unconjugated Bilirubin,” Molecular Biology Reports 50 (2023): 6703–6715. 37378749 10.1007/s11033-023-08576-1 PMC10374823

[126] J. Cai , X. Wang , Z. Wang , S. Sheng , F. Tang , and Z. Zhang , “ZC3H13‐Mediated m6A Modification Ameliorates Acute Myocardial Infarction Through Preventing Inflammation, Oxidative Stress and Ferroptosis by Targeting lncRNA93358,” Inflammation 48 (2025): 1270–1284. 39107569 10.1007/s10753-024-02116-0

[127] M. Fang , T. Li , and Z. Wu , “WTAP‐Mediated m6A Modification of KLF6 Aggravates Hypoxia/REOXYGENATION‐Induced Human CARDIOMYOCYTE Injury,” Shock 62 (2024): 201–207. 38662610 10.1097/SHK.0000000000002373

[128] C. Zhao and J. Li , “METTL14‐Mediated N6‐Methyladenosine Modification Induces the Ferroptosis of Hypoxia/Reoxygenation‐Induced Cardiomyocytes,” Journal of Cardiothoracic Surgery 19 (2024): 265. 38664788 10.1186/s13019-024-02711-0 PMC11044313

[129] M. L. Qiu , W. Yan , and M. M. Liu , “Klf6 Aggravates Myocardial Ischemia/Reperfusion Injury by Activating Acsl4‐Mediated Ferroptosis,” Kaohsiung Journal of Medical Sciences 39 (2023): 989–1001. 37530646 10.1002/kjm2.12733 PMC11895878

[130] J. Zhen , X. Sheng , T. Chen , and H. Yu , “Histone Acetyltransferase Kat2a Regulates Ferroptosis via Enhancing Tfrc and Hmox1 Expression in Diabetic Cardiomyopathy,” Cell Death & Disease 15 (2024): 406. 38858351 10.1038/s41419-024-06771-x PMC11164963

[131] Y. Yang , J. Ren , J. Zhang , H. Shi , J. Wang , and Y. Yan , “FTO Ameliorates Doxorubicin‐Induced Cardiotoxicity by Inhibiting Ferroptosis via P53‐P21/Nrf2 Activation in a HuR‐Dependent m6A Manner,” Redox Biology 70 (2024): 103067. 38316068 10.1016/j.redox.2024.103067 PMC10862061

[132] L. Wu , Y. Du , L. Wang , Y. Zhang , and J. Ren , “Inhibition of METTL3 Ameliorates Doxorubicin‐Induced Cardiotoxicity Through Suppression of TFRC‐Mediated Ferroptosis,” Redox Biology 72 (2024): 103157. 38631119 10.1016/j.redox.2024.103157 PMC11033199

[133] S. Zhuang , Y. Ma , Y. Zeng , et al., “METTL14 Promotes Doxorubicin‐Induced Cardiomyocyte Ferroptosis by Regulating the KCNQ1OT1‐miR‐7‐5p‐TFRC Axis,” Cell Biology and Toxicology 39 (2023): 1015–1035. 34648132 10.1007/s10565-021-09660-7

[134] W. Lin , H. Li , J. Chang , and Y. Huang , “ZC3H13 May Participate in the Ferroptosis Process of Sepsis‐Induced Cardiomyopathy by Regulating the Expression of Pnn and Rbm25,” Gene 933 (2025): 148944. 39284557 10.1016/j.gene.2024.148944

[135] H. Zeng , J. Xu , R. Wu , et al., “FTO Alleviated Ferroptosis in Septic Cardiomyopathy via Mediating the m6A Modification of BACH1,” Biochimica et Biophysica Acta‐Molecular Basis of Disease 1870 (2024): 167307. 38897256 10.1016/j.bbadis.2024.167307

[136] H. Shen , K. Xie , Y. Tian , and X. Wang , “N6‐Methyladenosine Writer METTL3 Accelerates the Sepsis‐Induced Myocardial Injury by Regulating m6A‐Dependent Ferroptosis,” Apoptosis 28 (2023): 514–524. 36645573 10.1007/s10495-022-01808-y

[137] X. Wu , P. Huang , Y. Xiao , L. Zha , J. Ma , and H. Xiao , “METTL14 Promotes Lipopolysaccharide‐Induced Myocardial Damage via m6A‐Dependent Stabilization of TRPM7 mRNA,” International Heart Journal 65 (2024): 1118–1127, 10.1536/ihj.24-162. 39617501

[138] M. Liao , S. Zou , J. Wu , et al., “METTL3‐Mediated m6A Modification of NORAD Inhibits the Ferroptosis of Vascular Smooth Muscle Cells to Attenuate the Aortic Dissection Progression in an YTHDF2‐Dependent Manner,” Molecular and Cellular Biochemistry 479, no. 12 (2024): 3471–3487. 38383916 10.1007/s11010-024-04930-4

[139] N. Li , X. Yi , Y. He , et al., “Targeting Ferroptosis as a Novel Approach to Alleviate Aortic Dissection,” International Journal of Biological Sciences 18 (2022): 4118–4134. 35844806 10.7150/ijbs.72528 PMC9274489

[140] W. Wang , J. Chen , S. Lai , et al., “METTL14 Promotes Ferroptosis in Smooth Muscle Cells During Thoracic Aortic Aneurysm by Stabilizing the m(6)A Modification of ACSL4,” American Journal of Physiology‐Cell Physiology 328 (2024): C387–C399, 10.1152/ajpcell.00577.2024. 39672203

[141] J. Lin , G. Zhan , J. Liu , et al., “YTHDF2‐Mediated Regulations Bifurcate BHPF‐Induced Programmed Cell Deaths,” National Science Review 10 (2023): nwad227. 38152479 10.1093/nsr/nwad227 PMC10751878

[142] J. Cai , X. Wang , Z. Wang , S. Sheng , F. Tang , and Z. Zhang , “ZC3H13‐Mediated m6A Modification Ameliorates Acute Myocardial Infarction Through Preventing Inflammation, Oxidative Stress and Ferroptosis by Targeting lncRNA93358,” Inflammation 48, no. 3 (2025): 1270–1284. 39107569 10.1007/s10753-024-02116-0

[143] S. Zhuang , Y. Ma , Y. Zeng , et al., “METTL14 Promotes Doxorubicin‐Induced Cardiomyocyte Ferroptosis by Regulating the KCNQ1OT1‐miR‐7‐5p‐TFRC Axis,” Cell Biology and Toxicology 39, no. 3 (2023): 1015–1035. 34648132 10.1007/s10565-021-09660-7

[144] K. W. Jia , R. Q. Yao , Y. W. Fan , et al., “Interferon‐α Stimulates DExH‐Box Helicase 58 to Prevent Hepatocyte Ferroptosis,” Military Medical Research 11 (2024): 22. 38622688 10.1186/s40779-024-00524-9 PMC11017495

[145] R. Li , X. Yan , C. Xiao , et al., “FTO Deficiency in Older Livers Exacerbates Ferroptosis During Ischaemia/Reperfusion Injury by Upregulating ACSL4 and TFRC,” Nature Communications 15 (2024): 4760. 10.1038/s41467-024-49202-3 PMC11150474 38834654

[146] M. Shen , Y. Li , Y. Wang , et al., “N(6)‐Methyladenosine Modification Regulates Ferroptosis Through Autophagy Signaling Pathway in Hepatic Stellate Cells,” Redox Biology 47 (2021): 102151. 34607160 10.1016/j.redox.2021.102151 PMC8495178

[147] X. Li , Y. Li , W. Zhang , et al., “The IGF2BP3/Notch/Jag1 Pathway: A Key Regulator of Hepatic Stellate Cell Ferroptosis in Liver Fibrosis,” Clinical and Translational Medicine 14 (2024): e1793. 39113232 10.1002/ctm2.1793 PMC11306284

[148] W. Liu , Y. He , K. Chen , et al., “YTHDF2 Influences Hepatic Fibrosis by Regulating Ferroptosis in Hepatic Stellate Cells by Mediating the Expression of ACSL4 in an m(6)A‐Dependent Manner,” Acta Biochimica et Biophysica Sinica Shanghai 57, no. 4 (2024): 521–528. 10.3724/abbs.2024162 PMC12040596 39716886

[149] F. Chen , M. Su , D. Han , Y. Wang , and M. Song , “METTL14 Depletion Ameliorates Ferroptosis in Severe Acute Pancreatitis by Increasing the N6‐Methyladenosine Modification of ACSL4 and STA1,” International Immunopharmacology 128 (2024): 111495. 38237228 10.1016/j.intimp.2024.111495

[150] F. Zhou , D. Li , C. Liu , et al., “m6A‐Activated BACH1 Exacerbates Ferroptosis by Epigenetic Suppression HSPB1 in Severe Acute Pancreatitis,” Drug Development Research 85 (2024): e22256. 39285641 10.1002/ddr.22256

[151] W. Liu and H. Zeng , “IGF2BP2 Attenuates Intestinal Epithelial Cell Ferroptosis in Colitis by Stabilizing m(6)A‐Modified GPX4 mRNA,” Cytokine 173 (2024): 156388. 38039694 10.1016/j.cyto.2023.156388

[152] M. Shen , Y. Li , Y. Wang , et al., “N6‐Methyladenosine Modification Regulates Ferroptosis Through Autophagy Signaling Pathway in Hepatic Stellate Cells,” Redox Biology 47 (2021): 102151. 34607160 10.1016/j.redox.2021.102151 PMC8495178

[153] L. Li and Z. Zhu , “Pharmacological Modulation of Ferroptosis as a Therapeutic Target for Liver Fibrosis,” Frontiers in Pharmacology 13 (2022): 1071844. 36703745 10.3389/fphar.2022.1071844 PMC9871257

[154] D. Wu , C. B. Spencer , L. Ortoga , H. Zhang , and C. Miao , “Histone Lactylation‐Regulated METTL3 Promotes Ferroptosis via m6A‐Modification on ACSL4 in Sepsis‐Associated Lung Injury,” Redox Biology 74 (2024): 103194. 38852200 10.1016/j.redox.2024.103194 PMC11219935

[155] H. Zhang , D. Wu , Y. Wang , et al., “METTL3‐Mediated N6‐Methyladenosine Exacerbates Ferroptosis via m6A‐IGF2BP2‐Dependent Mitochondrial Metabolic Reprogramming in Sepsis‐Induced Acute Lung Injury,” Clinical and Translational Medicine 13 (2023): e1389. 37715457 10.1002/ctm2.1389 PMC10504453

[156] A. Sang , J. Zhang , M. Zhang , et al., “METTL4 Mediated‐N6‐Methyladenosine Promotes Acute Lung Injury by Activating Ferroptosis in Alveolar Epithelial Cells,” Free Radical Biology & Medicine 213 (2024): 90–101. 38224757 10.1016/j.freeradbiomed.2024.01.013

[157] H. Zhang , J. Liu , Y. Zhou , et al., “Neutrophil Extracellular Traps Mediate m(6)A Modification and Regulates Sepsis‐Associated Acute Lung Injury by Activating Ferroptosis in Alveolar Epithelial Cells,” International Journal of Biological Sciences 18, no. 8 (2022): 3337–3357. 35637949 10.7150/ijbs.69141 PMC9134924

[158] Y. Zhao , W. Ding , Y. Cai , et al., “The m(6)A Eraser FTO Suppresses Ferroptosis via Mediating ACSL4 in LPS‐Induced Macrophage Inflammation,” Biochimica et Biophysica Acta‐Molecular Basis of Disease 1870 (2024): 167354. 39004378 10.1016/j.bbadis.2024.167354

[159] W. Deng , L. Zhong , S. Ye , et al., “Mir22hg Facilitates Ferritinophagy‐Mediated Ferroptosis in Sepsis by Recruiting the m6A Reader YTHDC1 and Enhancing Angptl4 mRNA Stability,” Journal of Bioenergetics and Biomembranes 56 (2024): 405–418. 38842666 10.1007/s10863-024-10022-1 PMC11217081

[160] J. Lai , S. Yu , X. Li , Q. Wei , and J. Qin , “METTL14/IGF2BP2‐Mediated m6A Modification of STEAP1 Aggravates Acute Lung Injury Induced by Sepsis,” Shock 63 (2025): 217–225. 39193903 10.1097/SHK.0000000000002456

[161] X. Fan , C. Wei , and Y. Han , “ALKBH5 Modulates Asthma Progression by Downregulating N6‐Methyladenosine Methylation,” Iranian Journal of Allergy, Asthma, and Immunology 23 (2024): 211–219. 38822515 10.18502/ijaai.v23i2.15326

[162] L. Lin , X. Hu , Q. Li , and L. Huang , “Methyltransferase‐Like 3 (METTL3) Epigenetically Modulates Glutathione Peroxidase 4 (GPX4) Expression to Affect Asthma,” Iranian Journal of Allergy, Asthma, and Immunology 22 (2023): 551–560. 38477952 10.18502/ijaai.v22i6.14644

[163] H. Xia , Y. Wu , J. Zhao , et al., “N6‐Methyladenosine‐Modified circSAV1 Triggers Ferroptosis in COPD Through Recruiting YTHDF1 to Facilitate the Translation of IREB2,” Cell Death and Differentiation 30, no. 5 (2023): 1293–1304. 36828914 10.1038/s41418-023-01138-9 PMC10154389

[164] Y. Yang , W. Shen , Z. Zhang , et al., “FSP1 Acts in Parallel With GPX4 to Inhibit Ferroptosis in COPD,” American Journal of Respiratory Cell and Molecular Biology 72 (2025): 551–562. 39453438 10.1165/rcmb.2023-0467OC PMC12051924

[165] H. Zhang , J. Liu , Y. Zhou , et al., “Neutrophil Extracellular Traps Mediate m6A Modification and Regulates Sepsis‐Associated Acute Lung Injury by Activating Ferroptosis in Alveolar Epithelial Cells,” International Journal of Biological Sciences 18, no. 8 (2022): 3337–3357. 35637949 10.7150/ijbs.69141 PMC9134924

[166] J. Lai , S. Yu , X. Li , Q. Wei , and J. Qin , “METTL14/IGF2BP2‐Mediated m6A Modification of STEAP1 Aggravates Acute Lung Injury Induced by Sepsis,” Shock 63, no. 2 (2025): 217–225. 39193903 10.1097/SHK.0000000000002456

[167] C. Hu , B. Zhang , and S. Zhao , “METTL3‐Mediated N6‐Methyladenosine Modification Stimulates Mitochondrial Damage and Ferroptosis of Kidney Tubular Epithelial Cells Following Acute Kidney Injury by Modulating the Stabilization of MDM2‐p53‐LMNB1 Axis,” European Journal of Medicinal Chemistry 259 (2023): 115677. 37542992 10.1016/j.ejmech.2023.115677

[168] F. Huang , Y. Wang , X. Lv , and C. Huang , “WTAP‐Mediated N6‐Methyladenosine Modification Promotes the Inflammation, Mitochondrial Damage and Ferroptosis of Kidney Tubular Epithelial Cells in Acute Kidney Injury by Regulating LMNB1 Expression and Activating NF‐κB and JAK2/STAT3 Pathways,” Journal of Bioenergetics and Biomembranes 56 (2024): 285–296. 38517565 10.1007/s10863-024-10015-0

[169] Z. Zhao , S. Dong , Y. Yang , et al., “IGF2BP1 Bolsters the Chondrocytes Ferroptosis of Osteoarthritis by Targeting m(6)A/MMP3 Axis,” International Journal of General Medicine 17 (2024): 2433–2443. 38826510 10.2147/IJGM.S463734 PMC11141773

[170] K. Habaxi , W. Wang , M. Taximaimaiti , and L. Wang , “Methylation Regulation of LPCAT3 Improves Osteoarthritis by Regulating ACSL4 to Inhibit Chondrocyte Ferroptosis,” Critical Reviews in Eukaryotic Gene Expression 34, no. 2 (2024): 77–86. 38073444 10.1615/CritRevEukaryotGeneExpr.2023049244

[171] T. Bao , T. Liao , X. Cai , et al., “METTL3 Mediated Ferroptosis in Chondrocytes and Promoted Pain in KOA via HMGB1 m6A Modification,” Cell Biology International 48, no. 11 (2024): 1755–1765. 39129231 10.1002/cbin.12229

[172] D. Liu , L. Ren , and J. Liu , “METTL14 Promotes Chondrocyte Ferroptosis in Osteoarthritis via m6A Modification of GPX4,” International Journal of Rheumatic Diseases 27 (2024): e15297. 39175261 10.1111/1756-185X.15297

[173] M. Song , K. Lv , Z. Xu , et al., “N6 Methyladenosine Eraser FTO Suppresses *Staphylococcus aureus* ‐Induced Ferroptosis of Bone Marrow Mesenchymal Stem Cells to Ameliorate Osteomyelitis Through Regulating the MDM2/TLR4/SLC7A11 Signaling Pathway,” Cell Biology International 48 (2024): 450–460. 38165230 10.1002/cbin.12115

[174] X. Liu , N. Wang , S. Gu , and Z. He , “Changes of RNA m(6)A/m(5)C Modification Regulatory Molecules in Ferroptosis of T2DM Rat Pancreas,” Cell Biochemistry and Biophysics 82 (2024): 1279–1289. 38709441 10.1007/s12013-024-01282-0

[175] Y. Lin , X. Shen , Y. Ke , et al., “Activation of Osteoblast Ferroptosis via the METTL3/ASK1‐p38 Signaling Pathway in High Glucose and High Fat (HGHF)‐Induced Diabetic Bone Loss,” FASEB Journal 36 (2022): e22147. 35104016 10.1096/fj.202101610R

[176] Y. Shi , L. Yin , J. Li , et al., “FTO Mediates Bisphenol F‐Induced Blood‐Testis Barrier Impairment Through Regulating Ferroptosis via YTHDF1/TfRc and YTHDF2/SLC7A11 Signal Axis,” Environmental Pollution (Barking, Essex: 1987) 359 (2024): 124531. 38996995 10.1016/j.envpol.2024.124531

[177] X. D. Zhang , J. Sun , X. M. Zheng , et al., “Plin4 Exacerbates Cadmium‐Decreased Testosterone Level via Inducing Ferroptosis in Testicular Leydig Cells,” Redox Biology 76 (2024): 103312. 39173539 10.1016/j.redox.2024.103312 PMC11387904

[178] Y. Wang , G. Zhang , Y. Gao , X. Zhang , and H. Qi , “METTL3 Promotes Trophoblast Ferroptosis in Preeclampsia by Stabilizing the ACSL4 m(6)A Modification,” Experimental Cell Research 437 (2024): 113990. 38462207 10.1016/j.yexcr.2024.113990

[179] X. Lu , D. Li , Z. Lin , et al., “HIF‐1α‐Induced Expression of the m6A Reader YTHDF1 Inhibits the Ferroptosis of Nucleus Pulposus Cells by Promoting SLC7A11 Translation,” Aging Cell 23, no. 9 (2024): e14210. 38783692 10.1111/acel.14210 PMC11488328

[180] H. Wang , C. Wang , Z. Zhu , and R. Li , “The Role of N6‐Methyladenosine(m(6)A) Mediated LncRNA DDIT3 Upregulation in Chondrocyte Ferroptosis in Ankylosing Spondylitis,” Asian Journal of Surgery 48, no. 24 (2024): 1377. 10.1016/j.asjsur.2024.08.172 39237414

[181] Z. Zhao , S. Dong , Y. Yang , H. Yin , G. Xiong , and J. Ma , “IGF2BP1 Bolsters the Chondrocytes Ferroptosis of Osteoarthritis by Targeting m6A/MMP3 Axis,” International Journal of General Medicine 17 (2024): 2433–2443. 38826510 10.2147/IJGM.S463734 PMC11141773

[182] J. Zhang , J. Song , S. Liu , et al., “m6A Methylation‐Mediated PGC‐1α Contributes to Ferroptosis via Regulating GSTK1 in Arsenic‐Induced Hepatic Insulin Resistance,” Science of the Total Environment 905 (2023): 167202. 37730054 10.1016/j.scitotenv.2023.167202

[183] Y. Wang , G. Zhang , Y. Gao , X. Zhang , and H. Qi , “METTL3 Promotes Trophoblast Ferroptosis in Preeclampsia by Stabilizing the ACSL4 m6A Modification,” Experimental Cell Research 437, no. 1 (2024): 113990. 38462207 10.1016/j.yexcr.2024.113990

[184] Z. Song , G. Jia , P. Ma , et al., “Exosomal miR‐4443 Promotes Cisplatin Resistance in Non‐Small Cell Lung Carcinoma by Regulating FSP1 m6A Modification‐Mediated Ferroptosis,” Life Sciences 276 (2021): 119399. 33781830 10.1016/j.lfs.2021.119399

[185] X. Xu , J. Cui , H. Wang , et al., “IGF2BP3 Is an Essential N(6)‐Methyladenosine Biotarget for Suppressing Ferroptosis in Lung Adenocarcinoma Cells,” Materials Today Bio 17 (2022): 100503. 10.1016/j.mtbio.2022.100503 PMC9707255 36457846

[186] Y. Xu , D. Lv , C. Yan , et al., “METTL3 Promotes Lung Adenocarcinoma Tumor Growth and Inhibits Ferroptosis by Stabilizing SLC7A11 m(6)A Modification,” Cancer Cell International 22, no. 1 (2022): 11. 34996469 10.1186/s12935-021-02433-6 PMC8742440

[187] M. Hu , J. Yang , and Z. Tan , “ATF1 Promotes Ferroptosis Resistance in Lung Cancer Through Enhancing mRNA Stability of PROM2,” Experimental Cell Research 442 (2024): 114190. 39098467 10.1016/j.yexcr.2024.114190

[188] Z. Liu , C. Jing , and W. Zhang , “METTL3‐Mediated m6A Modification Enhances ZDHHC16 Expression in Nonsmall‐ Cell Lung Cancer Patients, Attenuating Ferroptosis by Suppressing CREB Ubiquitination,” Cellular and Molecular Biology 70 (2024): 30–37. 10.14715/cmb/2024.70.2.5 38430044

[189] Y. Lou , K. Huang , B. Xu , and X. Chen , “METTL14 Plays an Oncogenic Role in NSCLC by Modulating Ferroptosis and the m6A Modification of GPX4,” Archives of Physiology and Biochemistry 130 (2024): 962–973. 38993012 10.1080/13813455.2024.2376813

[190] Y. Wu , H. Li , Y. Huang , and Q. Chen , “Silencing of m(6)A Methyltransferase KIAA1429 Suppresses the Progression of Non‐Small Cell Lung Cancer by Promoting the p53 Signaling Pathway and Ferroptosis,” American Journal of Cancer Research 13 (2023): 5320–5333. 38058803 PMC10695787

[191] Z. Huang , G. Lin , Y. Hong , L. Weng , K. Zhu , and W. Zhuang , “High Expression of AlkB Homolog 5 Suppresses the Progression of Non‐Small Cell Lung Cancer by Facilitating Ferroptosis Through m6A Demethylation of SLC7A11,” Environmental Toxicology 39 (2024): 4035–4046. 38642004 10.1002/tox.24272

[192] P. Li , D. Chu , G. Ding , et al., “IGF2BP3 Suppresses Ferroptosis in Lung Adenocarcinoma by m6A‐Dependent Regulation of TFAP2A to Transcriptionally Activate SLC7A11/GPX4,” Molecular and Cellular Biochemistry 480 (2025): 2361–2375. 39026029 10.1007/s11010-024-05068-z

[193] S. Yuan , S. Xi , H. Weng , et al., “YTHDC1 as a Tumor Progression Suppressor Through Modulating FSP1‐Dependent Ferroptosis Suppression in Lung Cancer,” Cell Death and Differentiation 30 (2023): 2477–2490. 37903990 10.1038/s41418-023-01234-w PMC10733405

[194] S. Xi , D. J. Ming , J. H. Zhang , et al., “Downregulation of N6‐Methyladenosine‐Modified LINC00641 Promotes EMT, but Provides a Ferroptotic Vulnerability in Lung Cancer,” Cell Death & Disease 14 (2023): 359. 37311754 10.1038/s41419-023-05880-3 PMC10264399

[195] H. Diao , H. Tan , Y. Hu , et al., “The m(6)A Reader YTHDF1 Promotes Lung Carcinoma Progression via Regulating Ferritin Mediate Ferroptosis in an m(6)A‐Dependent Manner,” Pharmaceuticals (Basel, Switzerland) 16 (2023): 185. 37259333 10.3390/ph16020185 PMC9966794

[196] L. Ma , T. Chen , X. Zhang , et al., “The m(6)A Reader YTHDC2 Inhibits Lung Adenocarcinoma Tumorigenesis by Suppressing SLC7A11‐Dependent Antioxidant Function,” Redox Biology 38 (2021): 101801. 33232910 10.1016/j.redox.2020.101801 PMC7691619

[197] L. Ma , X. Zhang , K. Yu , et al., “Targeting SLC3A2 Subunit of System X(C)(−) Is Essential for m(6)A Reader YTHDC2 To Be an Endogenous Ferroptosis Inducer in Lung Adenocarcinoma,” Free Radical Biology & Medicine 168 (2021): 25–43. 33785413 10.1016/j.freeradbiomed.2021.03.023

[198] D. Ding , W. Shang , K. Shi , et al., “FTO/m6A Mediates miR‐138‐5p Maturation and Regulates Gefitinib Resistance of Lung Adenocarcinoma Cells by miR‐138‐5p/LCN2 Axis,” BMC Cancer 24 (2024): 1270. 39394098 10.1186/s12885-024-13036-5 PMC11470737

[199] L. Liu , J. He , G. Sun , et al., “The N6‐Methyladenosine Modification Enhances Ferroptosis Resistance Through Inhibiting SLC7A11 mRNA Deadenylation in Hepatoblastoma,” Clinical and Translational Medicine 12, no. 5 (2022): e778. 35522946 10.1002/ctm2.778 PMC9076012

[200] J. Wang , M. Xiu , J. Wang , Y. Gao , and Y. Li , “METTL16‐SENP3‐LTF Axis Confers Ferroptosis Resistance and Facilitates Tumorigenesis in Hepatocellular Carcinoma,” Journal of Hematology & Oncology 17 (2024): 78. 39218945 10.1186/s13045-024-01599-6 PMC11367782

[201] Y. Li , M. Guo , Y. Qiu , et al., “Autophagy Activation Is Required for N6‐Methyladenosine Modification to Regulate Ferroptosis in Hepatocellular Carcinoma,” Redox Biology 69 (2024): 102971. 38056309 10.1016/j.redox.2023.102971 PMC10749285

[202] H. Gao , L. Shi , J. Liu , et al., “FOXM1‐Activated IGF2BP3 Promotes Cell Malignant Phenotypes and M2 Macrophage Polarization in Hepatocellular Carcinoma by Inhibiting Ferroptosis via Stabilizing RRM2 mRNA in an m6A‐Dependent Manner,” Molecular and Cellular Biochemistry 480 (2025): 3051–3066. 39630361 10.1007/s11010-024-05170-2

[203] H. Wang , W. Chen , Y. Cui , H. Gong , and H. Li , “KIAA1429 Protects Hepatocellular Carcinoma Cells From Ferroptotic Cell Death With a m(6) A‐Dependent Posttranscriptional Modification of SLC7A11,” Journal of Cellular and Molecular Medicine 27 (2023): 4118–4132. 37830241 10.1111/jcmm.17997 PMC10746954

[204] S. Chen , H. Xia , and L. Sheng , “WTAP‐Mediated m6A Modification on circCMTM3 Inhibits Hepatocellular Carcinoma Ferroptosis by Recruiting IGF2BP1 to Increase PARK7 Stability,” Digestive and Liver Disease 55 (2023): 967–981. 36586770 10.1016/j.dld.2022.12.005

[205] S. Liu , M. Shang , J. Gong , H. Sun , and B. Hu , “WTAP Regulates Mitochondrial Damage and Lipid Oxidation in HCC by NOA1 Mediated m6A Modification,” Journal of Cancer 16 (2025): 315–330, 10.7150/jca.102618. 39744575 PMC11660133

[206] D. Jin , Y. Hui , D. Liu , et al., “LINC00942 Inhibits Ferroptosis and Induces the Immunosuppression of Regulatory T Cells by Recruiting IGF2BP3/SLC7A11 in Hepatocellular Carcinoma,” Functional & Integrative Genomics 24 (2024): 29. 38353724 10.1007/s10142-024-01292-4 PMC10867055

[207] J. Yuan , T. Lv , J. Yang , et al., “HDLBP‐Stabilized lncFAL Inhibits Ferroptosis Vulnerability by Diminishing Trim69‐Dependent FSP1 Degradation in Hepatocellular Carcinoma,” Redox Biology 58 (2022): 102546. 36423520 10.1016/j.redox.2022.102546 PMC9692041

[208] Z. Lu , H. Yang , Y. Shao , et al., “IGF2BP3‐NRF2 Axis Regulates Ferroptosis in Hepatocellular Carcinoma,” Biochemical and Biophysical Research Communications 627 (2022): 103–110. 36030651 10.1016/j.bbrc.2022.08.040

[209] Q. Zhang , L. Xiong , T. Wei , et al., “Hypoxia‐Responsive PPARGC1A/BAMBI/ACSL5 Axis Promotes Progression and Resistance to Lenvatinib in Hepatocellular Carcinoma,” Oncogene 42 (2023): 1509–1523. 36932115 10.1038/s41388-023-02665-y

[210] Y. Zhao , S. Han , Z. Zeng , et al., “Decreased lncRNA HNF4A‐AS1 Facilitates Resistance to Sorafenib‐Induced Ferroptosis of Hepatocellular Carcinoma by Reprogramming Lipid Metabolism,” Theranostics 14 (2024): 7088–7110, 10.7150/thno.99197. 39629121 PMC11610135

[211] S. Zhao , J. Cao , R. Liang , et al., “METTL16 Suppresses Ferroptosis in Cholangiocarcinoma by Promoting ATF4 via m(6)A Modification,” International Journal of Biological Sciences 21 (2025): 189–203, 10.7150/ijbs.97886. 39744432 PMC11667817

[212] G. Zhu , Y. Xie , Z. Bian , et al., “N6‐Methyladenosine Modification of LATS2 Promotes Hepatoblastoma Progression by Inhibiting Ferroptosis Through the YAP1/ATF4/PSAT1 Axis,” International Journal of Biological Sciences 20, no. 11 (2024): 4146–4161. 39247829 10.7150/ijbs.92413 PMC11379071

[213] H. Ren , M. Wang , X. Ma , L. An , Y. Guo , and H. Ma , “METTL3 in Cancer‐Associated Fibroblasts‐Derived Exosomes Promotes the Proliferation and Metastasis and Suppresses Ferroptosis in Colorectal Cancer by Eliciting ACSL3 m6A Modification,” Biology Direct 19 (2024): 68. 39160584 10.1186/s13062-024-00511-z PMC11331890

[214] Y. Bian , S. Xu , Z. Gao , et al., “M(6)A Modification of lncRNA ABHD11‐AS1 Promotes Colorectal Cancer Progression and Inhibits Ferroptosis Through TRIM21/IGF2BP2/FOXM1 Positive Feedback Loop,” Cancer Letters 596 (2024): 217004. 38838765 10.1016/j.canlet.2024.217004

[215] Y. Qiao , M. Su , H. Zhao , et al., “Targeting FTO Induces Colorectal Cancer Ferroptotic Cell Death by Decreasing SLC7A11/GPX4 Expression,” Journal of Experimental & Clinical Cancer Research 43 (2024): 108. 38600610 10.1186/s13046-024-03032-9 PMC11005233

[216] J. Luo , H. Yu , Z. Yuan , T. Ye , and B. Hu , “ALKBH5 Decreases SLC7A11 Expression by Erasing m6A Modification and Promotes the Ferroptosis of Colorectal Cancer Cells,” Clinical & Translational Oncology 25 (2023): 2265–2276. 36820954 10.1007/s12094-023-03116-6

[217] H. Yang , Y. Hu , M. Weng , et al., “Hypoxia Inducible lncRNA‐CBSLR Modulates Ferroptosis Through m6A‐YTHDF2‐Dependent Mo Dulation of CBS in Gastric Cancer,” Journal of Advanced Research 37 (2022): 91–106. 35499052 10.1016/j.jare.2021.10.001 PMC9039740

[218] L. Niu , Y. Li , G. Huang , W. Huang , J. Fu , and L. Feng , “FAM120A Deficiency Improves Resistance to Cisplatin in Gastric Cancer by Promoting Ferroptosis,” Communications Biology 7 (2024): 399. 38565940 10.1038/s42003-024-06097-6 PMC10987584

[219] Y. Cui , M. Pu , Y. Gong , et al., “METTL3‐Driven m6A Modification of lncRNA FAM230B Suppresses Ferroptosis by Modulating miR‐27a‐5p/BTF3 Axis in Gastric Cancer,” Biochimica et Biophysica Acta, General Subjects 1868 (2024): 130714. 39278369 10.1016/j.bbagen.2024.130714

[220] X. Li , G. Yang , L. Ma , B. Tang , and T. Tao , “N(6)‐Methyladenosine (m(6)A) Writer METTL5 Represses the Ferroptosis and Antitumor Immunity of Gastric Cancer,” Cell Death Discov 10 (2024): 402. 39261486 10.1038/s41420-024-02166-1 PMC11390903

[221] F. H. Ji , X. H. Fu , G. Q. Li , et al., “FTO Prevents Thyroid Cancer Progression by SLC7A11 m6A Methylation in a Ferroptosis‐Dependent Manner,” Frontiers in Endocrinology 13 (2022): 857765. 35721711 10.3389/fendo.2022.857765 PMC9205202

[222] L. Luo , Y. Sun , and Z. Cao , “METTL3‐Induced m6A Modification Enhances Hsa_Circ_0136959 Expression to Impair the Tumor Characteristics of Papillary Thyroid Carcinoma via Accelerating Ferroptosis,” DNA and Cell Biology 44 (2024): 99–108, 10.1089/dna.2024.0197. 39623910

[223] F. Ye , J. Wu , and F. Zhang , “METTL16 Epigenetically Enhances GPX4 Expression via m6A Modification to Promote Breast Cancer Progression by Inhibiting Ferroptosis,” Biochemical and Biophysical Research Communications 638 (2023): 1–6. 36434904 10.1016/j.bbrc.2022.10.065

[224] Y. Zou , S. Zheng , X. Xie , et al., “N6‐Methyladenosine Regulated FGFR4 Attenuates Ferroptotic Cell Death in Recalcitrant HER2‐Positive Breast Cancer,” Nature Communications 13, no. 1 (2022): 2672. 10.1038/s41467-022-30217-7 PMC9106694 35562334

[225] Z. Yan , Z. Liang , K. Luo , et al., “METTL3‐Modified lncRNA DSCAM‐AS1 Promotes Breast Cancer Progression Through Inhibiting Ferroptosis,” Journal of Bioenergetics and Biomembranes 56 (2024): 451–459. 38833042 10.1007/s10863-024-10024-z

[226] M. Tan , Y. He , J. Yi , et al., “WTAP Mediates NUPR1 Regulation of LCN2 Through m(6)A Modification to Influence Ferroptosis, Thereby Promoting Breast Cancer Proliferation, Migration and Invasion,” Biochemical Genetics 62 (2024): 876–891. 37477758 10.1007/s10528-023-10423-8

[227] S. Wang , Y. Wang , Q. Li , K. Zeng , X. Li , and X. Feng , “RUNX1‐IT1 Favors Breast Cancer Carcinogenesis Through Regulation of IGF2BP1/GPX4 Axis,” Discover Oncology 14 (2023): 42. 37036576 10.1007/s12672-023-00652-z PMC10086083

[228] Y. Shao , Y. Chan , and R. Zhao , “SH3BP5‐AS1/IGF2BP2/VDAC2 Axis Promotes the Apoptosis and Ferroptosis of Bladder Cancer Cells,” Bladder Cancer 9 (2023): 29–40. 38994477 10.3233/BLC-211629 PMC11181683

[229] N. Wang , B. Shi , L. Ding , et al., “FMRP Protects Breast Cancer Cells From Ferroptosis by Promoting SLC7A11 Alternative Splicing Through Interacting With hnRNPM,” Redox Biology 77 (2024): 103382. 39388855 10.1016/j.redox.2024.103382 PMC11497378

[230] L. Li , J. Zeng , S. He , Y. Yang , and C. Wang , “METTL14 Decreases FTH1 mRNA Stability via m6A Methylation to Promote Sorafenib‐Induced Ferroptosis of Cervical Cancer,” Cancer Biology & Therapy 25 (2024): 2349429. 38738555 10.1080/15384047.2024.2349429 PMC11093024

[231] Y. Gong , G. Luo , S. Zhang , Y. Chen , and Y. Hu , “Transcriptome Sequencing Analysis Reveals miR‐30c‐5p Promotes Ferroptosis in Cervical Cancer and Inhibits Growth and Metastasis of Cervical Cancer Xenografts by Targeting the METTL3/KRAS Axis,” Cellular Signalling 117 (2024): 111068. 38286198 10.1016/j.cellsig.2024.111068

[232] Y. Liu , J. Li , J. Xu , et al., “M(6)A‐Driven NAT10 Translation Facilitates Fatty Acid Metabolic Rewiring to Suppress Ferroptosis and Promote Ovarian Tumorigenesis Through Enhancing ACOT7 mRNAc Acetylation,” Oncogene 43 (2024): 3498–3516. 39390256 10.1038/s41388-024-03185-z

[233] J. Jiang , J. Zhu , P. Qiu , J. Ni , W. Zhu , and X. Wang , “HNRNPA2B1‐Mediated m6A Modification of FOXM1 Promotes Drug Resistance and Inhibits Ferroptosis in Endometrial Cancer via Regulation of LCN2,” Functional & Integrative Genomics 24 (2023): 3. 38091112 10.1007/s10142-023-01279-7

[234] Y. Wang , C. Wang , X. Guan , et al., “PRMT3‐Mediated Arginine Methylation of METTL14 Promotes Malignant Progression and Treatment Resistance in Endometrial Carcinoma,” Advanced Science 10 (2023): e2303812. 37973560 10.1002/advs.202303812 PMC10754120

[235] Y. Jin , J. Qiu , X. Lu , Y. Ma , and G. Li , “LncRNA CACNA1G‐AS1 Up‐Regulates FTH1 to Inhibit Ferroptosis and Promote Malignant Phenotypes in Ovarian Cancer Cells,” Oncology Research 31 (2023): 169–179. 37304234 10.32604/or.2023.027815 PMC10208029

[236] Y. Wang , P. Jin , and X. Wang , “N(6)‐Methyladenosine Regulator YTHDF1 Represses the CD8 + T Cell‐Mediated Antitumor Immunity and Ferroptosis in Prostate Cancer via m(6)A/PD‐L1 Manner,” Apoptosis 29 (2024): 142–153. 37698736 10.1007/s10495-023-01885-7

[237] S. Sun , T. Gao , B. Pang , et al., “RNA Binding Protein NKAP Protects Gliob Lastoma Cells From Ferroptosis by Promoting SLC7A11 mRNA Splicing in an m(6)A‐Dependent Manner,” Cell Death & Disease 13, no. 1 (2022): 73. 35064112 10.1038/s41419-022-04524-2 PMC8783023

[238] D. Lv , C. Zhong , D. Dixit , et al., “EGFR Promotes ALKBH5 Nuclear Retention to Attenuate N6‐Methyladenosine and Protect Against Ferroptosis in Glioblastoma,” Molecular Cell 83 (2023): 4334–4351.e7. 37979586 10.1016/j.molcel.2023.10.025 PMC10842222

[239] X. Meng , Z. Wang , Q. Yang , et al., “Intracellular C5aR1 Inhibits Ferroptosis in Glioblastoma Through METTL3‐Dependent m6A Methylation of GPX4,” Cell Death & Disease 15 (2024): 729. 39368999 10.1038/s41419-024-06963-5 PMC11455874

[240] L. Deng , Y. Di , C. Chen , et al., “Depletion of the N(6)‐Methyladenosine (m6A) Reader Protein IGF2BP3 Induces Ferroptosis in Glioma by Modulating the Expression of GPX4,” Cell Death & Disease 15 (2024): 181. 38429265 10.1038/s41419-024-06486-z PMC10907351

[241] J. Zheng , Q. Zhang , Z. Zhao , et al., “Epigenetically Silenced lncRNA SNAI3‐AS1 Promotes Ferroptosis in Glioma via Perturbing the m(6)A‐Dependent Recognition of Nrf2 mRNA Mediated by SND1,” Journal of Experimental & Clinical Cancer Research 42 (2023): 127. 37202791 10.1186/s13046-023-02684-3 PMC10197824

[242] W. M. Huang , Z. X. Li , Y. H. Wu , et al., “m6A Demethylase FTO Renders Radioresistance of Nasopharyngeal Carcinoma via Promoting OTUB1‐Mediated Anti‐Ferroptosis,” Translational Oncology 27 (2023): 101576. 36343416 10.1016/j.tranon.2022.101576 PMC9646990

[243] J. Mi , Y. Wang , S. He , et al., “LncRNA HOTAIRM1 Promotes Radioresistance in Nasopharyngeal Carcinoma by Modulating FTO Acetylation‐Dependent Alternative Splicing of CD44,” Neoplasia 56 (2024): 101034. 39128424 10.1016/j.neo.2024.101034 PMC11367117

[244] J. Ye , X. Chen , X. Jiang , et al., “RNA Demethylase ALKBH5 Regulates Hypopharyngeal Squamous Cell Carcinoma Ferroptosis by Posttranscriptionally Activating NFE2L2/NRF2 in an m(6) A‐IGF2BP2‐Dependent Manner,” Journal of Clinical Laboratory Analysis 36, no. 7 (2022): e24514. 35689537 10.1002/jcla.24514 PMC9279968

[245] Z. Wang , H. Li , H. Cai , et al., “FTO Sensitizes Oral Squamous Cell Carcinoma to Ferroptosis via Suppressing ACSL3 and GPX4,” International Journal of Molecular Sciences 24 (2023): 16339. 38003537 10.3390/ijms242216339 PMC10671523

[246] K. Xu , X. Dai , and J. Yue , “M(6)A Methyltransferase KIAA1429 Accelerates Oral Squamous Cell Carcinoma via Regulating Glycolysis and Ferroptosis,” Translational Oncology 36 (2023): 101745. 37517144 10.1016/j.tranon.2023.101745 PMC10407427

[247] Y. Liang , H. Zhong , Y. Zhao , et al., “Epigenetic Mechanism of RBM15 in Affecting Cisplatin Resistance in Laryngeal Carcinoma Cells by Regulating Ferroptosis,” Biology Direct 19, no. 1 (2024): 57. 39039611 10.1186/s13062-024-00499-6 PMC11264397

[248] L. Huang , G. Chen , J. He , and P. Wang , “ZC3H13 Reduced DUOX1‐Mediated Ferroptosis in Laryngeal Squamous Cell Carcinoma Cells Through m6A‐Dependent Modification,” Tissue & Cell 84 (2023): 102187. 37536262 10.1016/j.tice.2023.102187

[249] H. Tong , X. Yu , D. Zhou , et al., “CircEZH2 Promotes Gallbladder Cancer Progression and Lipid Metabolism Reprogramming Through the miR‐556‐5p/SCD1 Axis,” iScience 27 (2024): 110428. 39129828 10.1016/j.isci.2024.110428 PMC11315105

[250] E. Hodara , A. Mades , L. Swartz , et al., “M(6)A Epitranscriptome Analysis Reveals Differentially Methylated Transcripts That Drive Early Chemoresistance in Bladder Cancer,” NAR Cancer 5 (2023): zcad054. 38023731 10.1093/narcan/zcad054 PMC10653028

[251] K. Wang , G. Wang , G. Li , et al., “m6A Writer WTAP Targets NRF2 to Accelerate Bladder Cancer Malignancy via m6A‐Dependent Ferroptosis Regulation,” Apoptosis 28 (2023): 627–638. 36719469 10.1007/s10495-023-01817-5

[252] Y. Jin , S. Pan , M. Wang , et al., “The m(6)A Modification of ACSL4 mRNA Sensitized Esophageal Squamous Cell Carcinoma to Irradiation via Accelerating Ferroptosis,” Cell Biology International 48, no. 12 (2024): 1877–1890. 39285560 10.1002/cbin.12245

[253] Y. Wu , H. Li , Y. Huang , and Q. Chen , “Silencing of m6A Methyltransferase KIAA1429 Suppresses the Progression of Non‐Small Cell Lung Cancer by Promoting the p53 Signaling Pathway and Ferroptosis,” American Journal of Cancer Research 13, no. 11 (2023): 5320–5333. 38058803 PMC10695787

[254] Y. Xu , D. Lv , C. Yan , et al., “METTL3 Promotes Lung Adenocarcinoma Tumor Growth and Inhibits Ferroptosis by Stabilizing SLC7A11 m6A Modification,” Cancer Cell International 22, no. 1 (2022): 11. 34996469 10.1186/s12935-021-02433-6 PMC8742440

[255] P. Li , D. Chu , G. Ding , D. Qin , Y. Bu , and B. Tian , “IGF2BP3 Suppresses Ferroptosis in Lung Adenocarcinoma by m6A‐Dependent Regulation of TFAP2A to Transcriptionally Activate SLC7A11/GPX4,” Molecular and Cellular Biochemistry 480, no. 4 (2025): 2361–2375. 39026029 10.1007/s11010-024-05068-z

[256] Y. Bian , S. Xu , Z. Gao , et al., “m6A Modification of lncRNA ABHD11‐AS1 Promotes Colorectal Cancer Progression and Inhibits Ferroptosis Through TRIM21/IGF2BP2/FOXM1 Positive Feedback Loop,” Cancer Letters 596 (2024): 217004. 38838765 10.1016/j.canlet.2024.217004

[257] Z. Jin , W. Gao , F. Guo , et al., “Astragaloside IV Alleviates Neuronal Ferroptosis in Ischemic Stroke by Regulating Fat Mass and Obesity‐Associated‐N6‐Methyladenosine‐Acyl‐CoA Synthetase Long‐Chain Family Member 4 Axis,” Journal of Neurochemistry 166 (2023): 328–345. 37300304 10.1111/jnc.15871

[258] T. Jiang , Y. Xiao , J. Zhou , et al., “Arbutin Alleviates Fatty Liver by Inhibiting Ferroptosis via FTO/SLC7A11 Pathway,” Redox Biology 68 (2023): 102963. 37984229 10.1016/j.redox.2023.102963 PMC10694775

[259] M. Shen , M. Guo , Y. Li , et al., “M(6)A Methylation Is Required for Dihydroartemisinin to Alleviate Liver Fibrosis by Inducing Ferroptosis in Hepatic Stellate Cells,” Free Radical Biology & Medicine 182 (2022): 246–259. 35248719 10.1016/j.freeradbiomed.2022.02.028

[260] S. Cheng , X. Xu , R. Wang , W. Chen , K. Qin , and J. Yan , “Chondroprotective Effects of Bone Marrow Mesenchymal Stem Cell‐Derived Exosomes in Osteoarthritis,” Journal of Bioenergetics and Biomembranes 56, no. 1 (2024): 31–44. 38012335 10.1007/s10863-023-09991-6

[261] B. Xiao , Y. Zhu , M. Liu , et al., “miR‐340‐3p‐Modified Bone Marrow Mesenchymal Stem Cell‐Derived Exosomes Inhibit Ferroptosis Through METTL3‐Mediated m(6)A Modification of HMOX1 to Promote Recovery of Injured Rat Uterus,” Stem Cell Research & Therapy 15 (2024): 224. 39075530 10.1186/s13287-024-03846-6 PMC11287883

[262] W. Jin , Y. Sun , J. Wang , et al., “Arsenic Trioxide Suppresses Lung Adenocarcinoma Stem Cell Stemness by Inhibiting m6A Modification to Promote Ferroptosis,” American Journal of Cancer Research 14 (2024): 507–525. 38455419 10.62347/KFAX9239 PMC10915325

[263] F. Wang , Z. Sun , Q. Zhang , et al., “Curdione Induces Ferroptosis Mediated by m6A Methylation via METTL14 and YTHDF2 in Colorectal Cancer,” Chinese Medicine 18 (2023): 122. 37735401 10.1186/s13020-023-00820-x PMC10512537

[264] G. Zhang , W. Mi , C. Wang , et al., “Targeting AKT Induced Ferroptosis Through FTO/YTHDF2‐Dependent GPX4 m6A Methylation Up‐Regulating and Degradating in Colorectal Cancer,” Cell Death Discov 9 (2023): 457. 38102129 10.1038/s41420-023-01746-x PMC10724184

[265] H. Shen , Z. Geng , X. Nie , and T. Liu , “Erianin Induces Ferroptosis of Renal Cancer Stem Cells via Promoting ALOX12/P53 mRNA N6‐Methyladenosine Modification,” Journal of Cancer 14 (2023): 367–378. 36860916 10.7150/jca.81027 PMC9969579

[266] P. Han , S. Wei , H. Wang , and Y. Cai , “Licochalcone A Decreases Cancer Cell Proliferation and Enhances Ferroptosis in Acute Myeloid Leukemia Through Suppressing the IGF2BP3/MDM2 Cascade,” Annals of Hematology 103, no. 11 (2024): 4511–4524. 39264435 10.1007/s00277-024-06003-4

[267] M. Shen , M. Guo , Y. Li , et al., “m6A Methylation Is Required for Dihydroartemisinin to Alleviate Liver Fibrosis by Inducing Ferroptosis in Hepatic Stellate Cells,” Free Radical Biology & Medicine 182 (2022): 246–259. 35248719 10.1016/j.freeradbiomed.2022.02.028

[268] B. Xiao , Y. Zhu , M. Liu , et al., “miR‐340‐3p‐Modified Bone Marrow Mesenchymal Stem Cell‐Derived Exosomes Inhibit Ferroptosis Through METTL3‐Mediated m6A Modification of HMOX1 to Promote Recovery of Injured Rat Uterus,” Stem Cell Research & Therapy 15, no. 1 (2024): 224. 39075530 10.1186/s13287-024-03846-6 PMC11287883

